# Network Meta‐Analysis: Efficacy of Biological Therapies and Small Molecules as Maintenance Therapy in Ulcerative Colitis

**DOI:** 10.1111/apt.70209

**Published:** 2025-05-23

**Authors:** Brigida Barberio, David J. Gracie, Christopher J. Black, Alexander C. Ford

**Affiliations:** ^1^ Department of Surgery, Oncology and Gastroenterology (DISCOG), Gastroenterology Unit University of Padova‐Azienda Ospedaliera di Padova Padova Italy; ^2^ Leeds Gastroenterology Institute St. James's University Hospital Leeds UK; ^3^ Leeds Institute of Medical Research St. James's, University of Leeds Leeds UK

**Keywords:** drugs, efficacy, RCT comparison, ulcerative colitis

## Abstract

**Background:**

Numerous biologics and small molecules are licensed as maintenance therapy for ulcerative colitis (UC). Differences in the design of randomised controlled trials (RCTs) have not been considered when comparing efficacy between them.

**Aims:**

To examine the relative efficacy of biologics and small molecules by network meta‐analysis according to trial design.

**Methods:**

We searched the literature to 27 February 2025 for RCTs. We judged efficacy using clinical remission, endoscopic improvement, endoscopic remission, or corticosteroid‐free remission and according to previous exposure or non‐exposure to advanced therapies. Random effects model with data reported as pooled relative risks (RR) with 95% confidence intervals (CI); drugs ranked by *p*‐score.

**Results:**

We identified 28 RCTs, 16 re‐randomising 6568 patients and 12 treating through 3771 patients. In re‐randomised studies, upadacitinib 30 mg o.d. ranked first for clinical remission (RR of failure to achieve clinical remission = 0.52; 95% CI 0.44–0.61, *p*‐score 0.99) and endoscopic improvement (RR = 0.43; 95% CI 0.35–0.52, *p*‐score 0.99). Vedolizumab 300 mg 4‐weekly ranked first for endoscopic remission (RR = 0.73; 95% CI 0.64–0.84, *p*‐score 0.92) and guselkumab 200 mg 4‐weekly first for corticosteroid‐free remission (RR = 0.40; 95% CI 0.28–0.55, *p*‐score 0.95). In treat‐through studies, etrasimod 2 mg o.d. ranked first for clinical remission (RR = 0.73; 95% CI 0.64–0.83, *p*‐score 0.88) and infliximab 10 mg/kg 8‐weekly first for endoscopic improvement (RR = 0.64; 95% CI 0.56–0.74, *p*‐score 0.94).

**Conclusion:**

In network meta‐analysis, upadacitinib and etrasimod were consistently efficacious as maintenance therapy in UC.

## Graphical abstract

This systematic review and network meta‐analysis compares biological therapies and small molecules as maintenance treatments for ulcerative colitis, assessing failure rates across clinical, endoscopic and histological outcomes. Findings reveal differential efficacy in patients naive to or previously exposed to advanced therapies, underscoring the importance of personalised maintenance strategies.

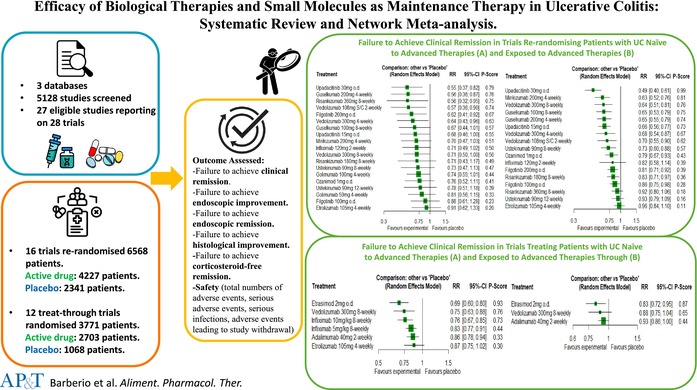

## 1 Introduction

Ulcerative colitis (UC) is a chronic, immune‐mediated inflammatory disease of the colon, characterised by periods of remission and relapse, leading to significant morbidity and impaired quality of life [1]. Effective long‐term management of UC relies on maintaining remission to prevent complications such as colectomy [2], hospitalisation [3], or corticosteroid dependence [4], which are associated with increased risks of infections, osteoporosis and metabolic disorders. Conventional therapies, including 5‐aminosalicylates and immunomodulators, often fail to sustain remission in patients with moderate to severe disease, necessitating the use of advanced therapies to re‐induce remission, including biological therapies targeting tumour necrosis factor (TNF)‐α, integrins, or interleukins, as well as small molecules such as Janus kinase (JAK) inhibitors and sphingosine‐1‐phosphate (S1P) receptor modulators [5, 6, 7, 8, 9].

Despite their efficacy in the induction phase, maintaining remission even with these therapies remains a major challenge, as response rates often decline over time due to primary or secondary loss of response [10]. The selection of the most appropriate maintenance therapy for UC is particularly complex due to heterogeneity in patient characteristics, previous exposure to other advanced therapies, and variability in clinical, endoscopic, and histological endpoints used in different randomised controlled trials (RCTs). Although individual trials have demonstrated the efficacy of these therapies in maintaining remission, direct head‐to‐head comparisons are scarce [11], making it difficult to determine the relative positioning of available drugs.

In recent years, network meta‐analyses have provided a comprehensive framework for indirect comparisons, allowing the evidence for efficacy to be compared across multiple interventions in numerous conditions [12]. However, most previous networks studying maintenance therapy in UC have focused primarily on clinical remission and endoscopic improvement, without incorporating a broader range of outcomes, such as corticosteroid‐free remission and histological response [13, 14, 15]. Additionally, existing evidence syntheses have not considered variations in study design adequately, such as whether trials used a re‐randomisation strategy or a treat‐through approach, and some have methodological issues.

To address these current gaps in the evidence, this systematic review and network meta‐analysis aimed to evaluate the comparative efficacy and safety of biological therapies and small molecules as maintenance therapy in UC. We compared the efficacy of different therapies across multiple outcomes, including clinical remission as our primary endpoint of interest, and endoscopic improvement or remission, histologic improvement, corticosteroid‐free remission and safety as secondary endpoints. Additionally, we assessed the impact of trial design in terms of re‐randomisation vs. treat‐through and prior exposure to advanced therapies on treatment rankings, to provide a more comprehensive understanding of optimal maintenance therapies for UC.

## 2 Methods

### 2.1 Search Strategy and Selection Criteria

We searched MEDLINE (1 January 1946–27 February 2025), EMBASE and EMBASE Classic (1 January 1947–27 February 2025), and the Cochrane Central Register of Controlled Trials. In addition, we searched clinicaltrials.gov for recently completed trials or Supporting Infromation S1 for potentially eligible RCTs. Conference proceedings (Digestive Diseases Week, American College of Gastroenterology, United European Gastroenterology Week and the Asian Pacific Digestive Week) between 2001 and 2024 were obtained as part of the electronic search and were used to identify trials published only in abstract form. Finally, we used bibliographies of all obtained articles to perform a recursive search.

Eligible RCTs examined the efficacy of biological therapies (anti‐TNFα antibodies [infliximab, adalimumab, or golimumab], anti‐integrin antibodies [vedolizumab or etrolizumab], anti‐interleukin‐12/23 antibodies [ustekinumab], or anti‐interleukin‐23 antibodies [mirikizumab, risankizumab, or guselkumab]) or small molecules (JAK inhibitors [tofacitinib, filgotinib, or upadacitinib] or S1P receptor modulators [ozanimod or etrasimod]), as maintenance therapy, at the doses taken through into testing in phase III clinical trials. Trials had to either randomise patients to active drug or placebo at baseline, or administer open‐label drug at baseline, with patients receiving active drug assessed for response subsequently and then responders being re‐randomised to active drug or placebo as maintenance therapy (trials re‐randomising patients), or be randomised to active drug or placebo at baseline, with treatment continued as maintenance through to the final point of follow‐up without re‐randomisation (trials treating patients through). Studies had to recruit ambulatory adults (≥ 18 years) with UC (Table S1), and compared biological therapies or small molecules with placebo, or with each other. We required a minimum follow‐up duration of 26 weeks, with endpoints of interest reported at completion of therapy.

Two investigators (BB and ACF) conducted independent literature searches. We identified maintenance studies on UC with: *colitis* or UC (both as medical subject headings and free text terms). We combined these using the set operator AND with studies identified with: *infliximab*, *remicade*, *adalimumab*, *humira*, *golimumab*, *simponi*, *vedolizumab*, *entyvio*, *etrolizumab*, *ustekinumab*, *stelara*, *mirikizumab*, *risankizumab*, *guselkumab*, *tofacitinib*, *xeljanz*, *filgotinib*, *upadacitinib*, *ozanimod*, or *etrasimod* and applied the clinical trials filter in OVID. There were no language restrictions. Two investigators (BB and ACF) assessed all identified abstracts independently. We obtained potentially relevant articles and evaluated them with pre‐designed forms, assessing eligibility independently according to our pre‐defined criteria. We translated foreign language papers if required. We resolved disagreements between investigators by discussion. As the study involved results of RCTs rather than human subjects, ethical approval was not required.

### 2.2 Outcome Assessment

We assessed the efficacy of biological therapies or small molecules, compared with placebo or each other, in terms of failure to achieve clinical remission as our primary endpoint of interest. We also assessed failure to achieve endoscopic improvement, failure to achieve endoscopic remission, failure to achieve histological improvement, failure to achieve histological remission, or failure to achieve corticosteroid‐free remission (only in those receiving corticosteroids at baseline) at the last point of follow‐up of the trial as secondary endpoints. Other secondary outcomes assessed included safety (total numbers of treatment‐emergent adverse events, as well as serious adverse events, serious infections and adverse events leading to study withdrawal), if reported.

### 2.3 Data Extraction

Two investigators (BB and ACF) extracted efficacy and safety data from all eligible studies independently from each other onto a Microsoft Excel spreadsheet (Microsoft 365; Microsoft Corp, Redmond, WA, USA) as dichotomous outcomes (clinical remission or no clinical remission, endoscopic improvement or no endoscopic improvement, etc.) at the last point of follow‐up as treatment was completed. We assessed efficacy according to the proportion of patients failing to achieve each of the endpoints of interest and safety as the proportion of patients experiencing each of the adverse events of interest. We also extracted the following data for each trial, where available: design, country of origin, number of centres, disease extent, proportion of patients who were naïve to advanced therapies, dose and treatment schedule of active therapy and placebo, and duration of follow‐up. We extracted efficacy data as intention‐to‐treat analyses, with dropouts assumed to be treatment failures (i.e., no clinical remission, no endoscopic improvement, etc. with biological therapy, small molecule, or placebo), wherever trial reporting allowed. If this was unclear in the original article, we performed an analysis on all evaluable patients. We compared the results of the two investigators' data extraction, with all discrepancies highlighted and resolved by discussion between the two investigators.

### 2.4 Assessment of Risk of Bias

We used the Cochrane risk of bias tool to assess this at the study level [16]. Two investigators (BB and ACF) performed this independently, resolving any disagreements by discussion. We recorded the method used to generate the randomisation schedule and conceal treatment allocation, as well as whether blinding was implemented for participants, personnel and outcomes assessment, whether there was evidence of incomplete outcomes data, and whether there was evidence of selective reporting of outcomes.

### 2.5 Data Synthesis and Statistical Analysis

Given it is not appropriate to compare the results of trials re‐randomising patients with those treating patients through, or to pool them together in a network meta‐analysis, we analysed trials separately, according to their design, for each endpoint. In each case, we performed a network meta‐analysis using the frequentist model, with the statistical package ‘netmeta’ (version 0.9‐0, https://cran.r‐project.org/web/packages/netmeta/index.html) in R (version 4.0.2), reporting this according to the PRISMA extension statement for network meta‐analyses [17]. We explored direct and indirect treatment comparisons of the efficacy and safety of each intervention. Network meta‐analysis results can give a more precise estimate, compared with those from standard, pairwise analyses [12, 18], and can rank interventions to inform clinical decisions [19].

We examined the symmetry and geometry of the evidence by producing a network plot with node size corresponding to the number of study subjects and connection size corresponding to the number of studies. We produced comparison adjusted funnel plots to assess for publication bias or other small study effects for all available comparisons. This is a scatterplot of effect size versus precision, measured via the inverse of the standard error, with symmetry around the effect estimate line indicating the absence of publication bias or small study effects [20], as judged by two reviewers (BB and ACF). Network and funnel plots were created using R (version 4.0.2). We used a pooled relative risk (RR) with 95% confidence intervals (CIs) to judge the efficacy of each comparison tested with a random effects model used as a conservative estimate. We used a RR of failure to achieve clinical remission or failure to achieve endoscopic improvement, etc. This approach is likely to be the most consistent across individual trials compared with a RR of cure or improvement or using the odds ratio (OR) for some meta‐analyses [21].

Many meta‐analyses use the *I* ^2^ statistic to measure heterogeneity, which ranges between 0% and 100% [22]. This statistic is easy to interpret and does not vary with the number of studies. However, its value can increase with the number of patients included in a meta‐analysis [23]. We, therefore, assessed global statistical heterogeneity across all comparisons using the τ^2^ measure from the ‘netmeta’ statistical package. Estimates of τ^2^ of 0.04, 0.16 and 0.36 are considered to represent low, moderate and high levels of heterogeneity, respectively [24]. We checked the correlation between direct and indirect evidence across the network via consistency modelling [25], generating network heat plots. These have grey squares representing the size of the contribution of the direct estimate of one study design in columns, compared with the network estimate in rows [26]. The coloured squares around these represent the change in inconsistency between direct and indirect evidence in a network estimate in the row after relaxing the consistency assumption for the effect of one design in the column. Blue squares indicate direct evidence in the column supports the indirect evidence in the row, yellow squares indicate no major inconsistency but some degree of disparity between direct and indirect evidence, and red squares are ‘hotspots’ of inconsistency between direct and indirect evidence.

We ranked all biological therapies and small molecules, versus placebo or each other, according to their *p*‐score, which is a value between 0 and 1, and based solely on point estimates and standard errors from the network estimates. It measures the mean extent of certainty that one intervention is better than another, averaged over all competing interventions [27], with higher scores indicating a greater probability of the intervention being ranked as best [27]. However, the magnitude of the *p*‐score should be considered, as well as the rank. As the mean value of the *p*‐score is always 0.5 if individual interventions cluster around this value, they are likely to be of similar efficacy. Of note, when interpreting the results, it is also important to take the RR and corresponding 95% CI for each comparison into account, rather than only relying on rankings [28]. In our primary analyses, we pooled data for all patients, but we also performed a priori subgroup analyses for each efficacy endpoint according to whether or not patients had been exposed to advanced therapies previously. Some RCTs only recruited patients that were naïve to advanced therapies, but among trials that did not restrict recruitment to this population, this information was reported in different ways. Most RCTs reported efficacy according to any prior exposure to advanced therapies, but a smaller number reported efficacy specifically according to previous failure of advanced therapies. As denominators for prior exposure and failure were similar, wherever trials reported both, and as the number of trials reporting prior exposure was higher, we extracted efficacy according to prior exposure, wherever possible.

For our primary analysis of failure to achieve clinical remission, we used the Confidence in Network Meta‐Analysis (CINeMA) framework to evaluate confidence in the indirect and direct treatment estimates from the network [29, 30], which is endorsed by the Cochrane Collaboration. This includes the Risk of Bias from Missing Evidence in Network Meta‐Analysis tool for evaluation of reporting bias [31].

## 3 Results

The search strategy generated 5128 citations, 92 of which appeared relevant and were retrieved for further assessment. Of these, we excluded 65 that did not fulfil eligibility criteria, with reasons provided in Figure S1, leaving 27 eligible articles [5, 6, 7, 8, 9, 11, 32, 33, 34, 35, 36, 37, 38, 39, 40, 41, 42, 43, 44, 45, 46, 47, 48, 49, 50, 51, 52], reporting on 28 trials. Sixteen re‐randomised 6568 patients responding to active drug administered at baseline to either active drug or placebo [6, 7, 9, 40, 41, 42, 43, 44, 45, 46, 47, 48, 49, 50, 51, 52], and 12 were treat‐through trials randomising 3771 patients to active drug or placebo at baseline, summarised in 11 articles [5, 8, 11, 32, 33, 34, 35, 36, 37, 38, 39]. Agreement between investigators for trial eligibility was excellent (kappa statistic = 0.86). Patients were allocated to active therapy or placebo as described in Tables S2 and S3, according to trial design. Two treat‐through trials were head‐to‐head studies, one of vedolizumab versus adalimumab [11], and the other of etrolizumab versus infliximab [37].

Detailed characteristics of individual RCTs, according to design, are provided in Tables S4 and S5. Among the 16 re‐randomised trials, seven reported the proportion of patients with previous exposure to advanced therapies [9, 40, 43, 44, 45, 47, 49], five the proportion with previous failure of advanced therapies [6, 7, 48, 50, 51], three recruited only patients naïve to advanced therapies [41, 42, 46], and one did not report this information at all [52]. Among the 12 treat‐through trials, eight recruited only patients naïve to advanced therapies [5, 8, 32, 33, 34, 35, 36, 37], three reported the proportion of patients with previous exposure to advanced therapies [8, 11, 38], and one did not report this information at all [39]. Risk of bias items for all included trials, according to design, are reported in Tables S6 and S7. Five of 12 treat‐through trials were at low risk of bias across all domains [8, 34, 36, 37, 38], and six of 16 re‐randomised trials [6, 40, 47, 48, 49, 51]. Endpoints reported in each of the trials, according to design, are reported in Tables S8 and S9. Two re‐randomised trials reported corticosteroid‐free remission data in separate articles [53, 54]. All endpoints for treat‐through trials, and safety endpoints for re‐randomised RCTs, are provided in the Supporting Infromation S1. Assessment of endoscopic remission or histological endpoints was not feasible in treat‐through trials, as only two RCTs, with no common comparator, reported endoscopic remission data [8, 37], and only three trials reported histological endpoints [8, 11, 38], but these were not comparable.

### 3.1 Failure to Achieve Clinical Remission in Trials Re‐Randomising Patients

**FIGURE 1:**
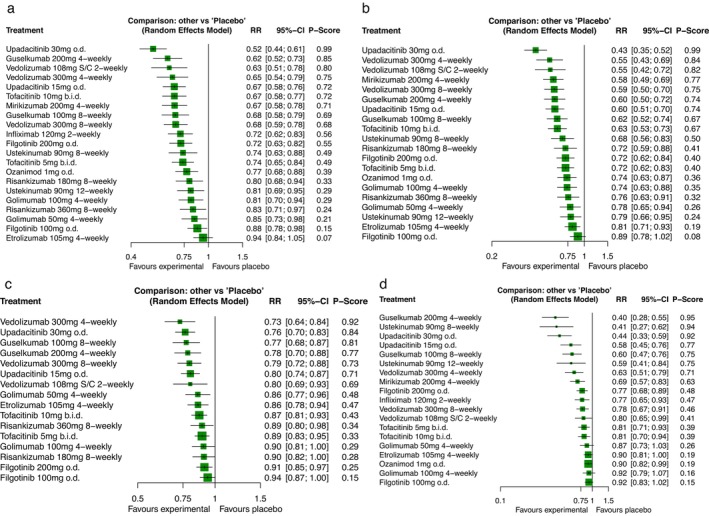
(a) Forest plot for failure to achieve (a) clinical remission, (b) endoscopic improvement, (c) endoscopic remission and (d) corticosteroid‐free remission in trials re‐randomising patients with UC.The *p*‐score is the probability of each intervention being ranked as best in the network.

The network plot for all 16 trials re‐randomising patients [6, 7, 9, 40, 41, 42, 43, 44, 45, 46, 47, 48, 49, 50, 51, 52], containing 6568 patients, for clinical remission is provided in Figure S2. There was low heterogeneity in this analysis (τ^2^ = 0.0011), but the funnel plot appeared asymmetrical (Figure S3), suggesting publication bias or other small study effects. All drugs and dosages, other than etrolizumab 105 mg 4‐weekly, were superior to placebo, but upadacitinib 30 mg o.d. ranked first (RR of failure to achieve clinical remission = 0.52; 95% CI 0.44–0.61, *p*‐score 0.99) (Figure 1a), meaning that the probability that upadacitinib was the most effective drug for clinical remission was 99%. Guselkumab 200 mg 4‐weekly ranked second (RR = 0.62; 95% CI 0.52–0.73, *p*‐score 0.85) and vedolizumab 108 mg subcutaneously 2‐weekly ranked third (RR = 0.63; 95% CI 0.51–0.78, *p*‐score 0.80). After direct and indirect comparison, upadacitinib 30 mg o.d. was superior to all drugs other than guselkumab 200 mg 4‐weekly, vedolizumab 108 mg subcutaneously 2‐weekly, and vedolizumab 300 mg 4‐weekly (Table 1), with moderate to high confidence in the results of all analyses according to the CINeMA framework (Table S10). Guselkumab 200 mg 4‐weekly was superior to ozanimod 1 mg o.d. (moderate confidence according to the CINeMA framework), risankizumab 360 mg or 180 mg 8‐weekly (moderate confidence for both), ustekinumab 90 mg 12‐weekly (moderate confidence), golimumab 100 mg or 50 mg 4‐weekly (moderate confidence for both), filgotinib 100 mg o.d. (high confidence), and etrolizumab 105 mg 4‐weekly (high confidence). Vedolizumab 108 mg subcutaneously 2‐weekly was superior to risankizumab 360 mg 8‐weekly (moderate confidence), golimumab 50 mg 4‐weekly (moderate confidence), filgotinib 100 mg o.d. (moderate confidence), and etrolizumab 105 mg 4‐weekly (moderate confidence).

**TABLE 1:** League table for failure to achieve clinical remission in trials re‐randomising patients with UC.

**UPA 30 mg o.d**.				0.78 [0.65; 0.94]																	0.52 [0.44; 0.61]
0.84 [0.67; 1.06]	**GUS 200 mg 4‐weekly**						0.91 [0.74; 1.12]														0.62 [0.52; 0.73]
0.83 [0.63; 1.08]	0.98 [0.75; 1.29]	**VED 108 mg S/C 2‐weekly**						0.94 [0.70; 1.26]													0.63 [0.51; 0.78]
0.80 [0.62; 1.01]	0.94 [0.73; 1.21]	0.96 [0.73; 1.27]	**VED 300 mg 4‐weekly**					0.95 [0.76; 1.19]													0.66 [0.54; 0.79]
0.78 [0.65; 0.94]	0.92 [0.74; 1.15]	0.94 [0.73; 1.21]	0.98 [0.78; 1.23]	**UPA 15 mg o.d**.																	0.67 [0.58; 0.76]
0.78 [0.63; 0.96]	0.92 [0.74; 1.15]	0.94 [0.73; 1.21]	0.98 [0.77; 1.23]	1.00 [0.82; 1.21]	**TOF 10 mg b.i.d**.							0.90 [0.77; 1.07]									0.67 [0.58; 0.77]
0.78 [0.62; 0.96]	0.92 [0.74; 1.15]	0.94 [0.73; 1.22]	0.98 [0.77; 1.24]	1.00 [0.82; 1.22]	1.00 [0.81; 1.22]	**MIR 200 mg 4‐weekly**															0.67 [0.58; 0.78]
0.77 [0.61; 0.96]	0.91 [0.74; 1.12]	0.93 [0.71; 1.21]	0.97 [0.76; 1.23]	0.99 [0.80; 1.22]	0.99 [0.80; 1.22]	0.99 [0.80; 1.23]	**GUS 100 mg 8‐weekly**														0.68 [0.58; 0.79]
0.77 [0.62; 0.95]	0.91 [0.73; 1.13]	0.93 [0.73; 1.18]	0.96 [0.78; 1.19]	0.98 [0.81; 1.19]	0.98 [0.81; 1.20]	0.99 [0.81; 1.21]	1.00 [0.81; 1.23]	**VED 300 mg 8‐weekly**													0.68 [0.59; 0.78]
0.72 [0.58; 0.90]	0.86 [0.69; 1.08]	0.88 [0.68; 1.13]	0.91 [0.72; 1.15]	0.93 [0.76; 1.13]	0.93 [0.76; 1.14]	0.93 [0.76; 1.15]	0.94 [0.76; 1.17]	0.95 [0.77; 1.16]	**IFX 120 mg 2‐weekly**												0.72 [0.62; 0.83]
0.72 [0.59; 0.89]	0.86 [0.69; 1.06]	0.87 [0.68; 1.12]	0.91 [0.72; 1.14]	0.93 [0.77; 1.12]	0.93 [0.76; 1.13]	0.93 [0.76; 1.13]	0.94 [0.76; 1.16]	0.94 [0.78; 1.14]	1.00 [0.82; 1.21]	**FIL 200 mg o.d**.									0.82 [0.71; 0.95]		0.72 [0.63; 0.82]
0.70 [0.56; 0.88]	0.83 [0.66; 1.06]	0.85 [0.65; 1.11]	0.88 [0.69; 1.13]	0.90 [0.73; 1.12]	0.90 [0.73; 1.12]	0.90 [0.72; 1.13]	0.91 [0.72; 1.15]	0.92 [0.74; 1.14]	0.97 [0.78; 1.21]	0.97 [0.79; 1.21]	**UST 90 mg 8‐weekly**				0.91 [0.76; 1.10]						0.74 [0.63; 0.88]
0.70 [0.57; 0.86]	0.84 [0.67; 1.03]	0.85 [0.66; 1.09]	0.88 [0.71; 1.11]	0.90 [0.75; 1.09]	0.90 [0.77; 1.07]	0.91 [0.75; 1.10]	0.92 [0.74; 1.12]	0.92 [0.76; 1.11]	0.97 [0.80; 1.18]	0.98 [0.81; 1.18]	1.00 [0.81; 1.24]	**TOF 5 mg b.i.d**.									0.74 [0.65; 0.84]
0.67 [0.55; 0.83]	0.80 [0.64; 0.99]	0.81 [0.63; 1.04]	0.84 [0.67; 1.06]	0.86 [0.71; 1.04]	0.86 [0.71; 1.05]	0.87 [0.71; 1.06]	0.87 [0.71; 1.08]	0.88 [0.72; 1.06]	0.93 [0.76; 1.13]	0.93 [0.77; 1.13]	0.96 [0.77; 1.19]	0.95 [0.79; 1.15]	**OZA 1 mg o.d**.								0.77 [0.68; 0.88]
0.65 [0.52; 0.82]	0.77 [0.61; 0.98]	0.79 [0.60; 1.03]	0.82 [0.64; 1.04]	0.84 [0.68; 1.03]	0.84 [0.68; 1.04]	0.84 [0.67; 1.04]	0.85 [0.67; 1.06]	0.85 [0.69; 1.05]	0.90 [0.72; 1.12]	0.90 [0.73; 1.11]	0.93 [0.74; 1.17]	0.93 [0.75; 1.14]	0.97 [0.79; 1.19]	**RIS 180 mg 8‐weekly**			0.96 [0.80; 1.14]				0.80 [0.68; 0.94]
0.64 [0.51; 0.80]	0.76 [0.60; 0.96]	0.78 [0.60; 1.01]	0.81 [0.63; 1.03]	0.82 [0.67; 1.01]	0.82 [0.67; 1.02]	0.83 [0.67; 1.03]	0.83 [0.67; 1.04]	0.84 [0.68; 1.03]	0.88 [0.71; 1.10]	0.89 [0.72; 1.09]	0.91 [0.76; 1.10]	0.91 [0.74; 1.12]	0.95 [0.78; 1.17]	0.98 [0.79; 1.23]	**UST 90 mg 12‐weekly**						0.81 [0.69; 0.95]
0.64 [0.52; 0.79]	0.76 [0.61; 0.95]	0.77 [0.60; 1.00]	0.80 [0.64; 1.01]	0.82 [0.67; 1.00]	0.82 [0.67; 1.00]	0.82 [0.67; 1.01]	0.83 [0.67; 1.03]	0.83 [0.68; 1.02]	0.88 [0.72; 1.08]	0.89 [0.73; 1.08]	0.91 [0.73; 1.13]	0.91 [0.75; 1.10]	0.95 [0.78; 1.16]	0.98 [0.79; 1.22]	1.00 [0.81; 1.23]	**GOL 100 mg 4‐weekly**		0.99 [0.84; 1.17]			0.81 [0.70; 0.94]
0.62 [0.50; 0.78]	0.74 [0.59; 0.93]	0.76 [0.58; 0.98]	0.78 [0.62; 1.00]	0.80 [0.65; 0.98]	0.80 [0.65; 0.99]	0.80 [0.65; 0.99]	0.81 [0.65; 1.01]	0.81 [0.66; 1.00]	0.86 [0.70; 1.06]	0.87 [0.71; 1.06]	0.89 [0.71; 1.12]	0.89 [0.73; 1.08]	0.93 [0.76; 1.14]	0.96 [0.80; 1.14]	0.97 [0.78; 1.21]	0.98 [0.79; 1.21]	**RIS 360 mg 8‐weekly**				0.83 [0.71; 0.97]
0.61 [0.49; 0.76]	0.73 [0.58; 0.91]	0.74 [0.57; 0.96]	0.77 [0.61; 0.98]	0.79 [0.64; 0.96]	0.79 [0.64; 0.97]	0.79 [0.64; 0.98]	0.80 [0.64; 0.99]	0.80 [0.65; 0.98]	0.85 [0.69; 1.04]	0.85 [0.70; 1.04]	0.87 [0.70; 1.09]	0.87 [0.72; 1.06]	0.91 [0.75; 1.12]	0.94 [0.76; 1.17]	0.96 [0.77; 1.19]	0.96 [0.81; 1.13]	0.98 [0.79; 1.22]	**GOL 50 mg 4‐weekly**			0.86 [0.74; 1.00]
0.59 [0.49; 0.72]	0.70 [0.57; 0.86]	0.72 [0.56; 0.91]	0.74 [0.60; 0.93]	0.76 [0.64; 0.91]	0.76 [0.63; 0.91]	0.76 [0.63; 0.92]	0.77 [0.63; 0.94]	0.77 [0.65; 0.93]	0.82 [0.68; 0.98]	0.82 [0.71; 0.95]	0.84 [0.69; 1.03]	0.84 [0.71; 1.00]	0.88 [0.74; 1.05]	0.91 [0.75; 1.11]	0.92 [0.76; 1.12]	0.93 [0.77; 1.12]	0.95 [0.78; 1.15]	0.97 [0.80; 1.17]	**FIL 100 mg o.d**.		0.88 [0.78; 0.98]
0.55 [0.45; 0.67]	0.66 [0.54; 0.81]	0.67 [0.53; 0.85]	0.70 [0.56; 0.86]	0.71 [0.60; 0.85]	0.71 [0.59; 0.85]	0.71 [0.59; 0.86]	0.72 [0.59; 0.88]	0.72 [0.60; 0.87]	0.76 [0.64; 0.92]	0.77 [0.64; 0.92]	0.79 [0.64; 0.97]	0.79 [0.66; 0.93]	0.82 [0.69; 0.98]	0.85 [0.70; 1.03]	0.86 [0.71; 1.05]	0.87 [0.72; 1.04]	0.89 [0.73; 1.07]	0.90 [0.75; 1.09]	0.93 [0.79; 1.10]	**ETRO 105 mg 4‐weekly**	0.94 [0.84; 1.05]
0.52 [0.44; 0.61]	0.62 [0.52; 0.73]	0.63 [0.51; 0.78]	0.65 [0.54; 0.79]	0.67 [0.58; 0.76]	0.67 [0.58; 0.77]	0.67 [0.58; 0.78]	0.68 [0.58; 0.79]	0.68 [0.59; 0.78]	0.72 [0.62; 0.83]	0.72 [0.63; 0.82]	0.74 [0.63; 0.88]	0.74 [0.65; 0.84]	0.77 [0.68; 0.88]	0.80 [0.68; 0.94]	0.81 [0.69; 0.95]	0.81 [0.70; 0.94]	0.83 [0.71; 0.97]	0.85 [0.73; 0.98]	0.88 [0.78; 0.98]	0.94 [0.84; 1.05]	**PLA**

*Note:* Relative risk with 95% confidence intervals in parentheses. Comparisons, column versus row, should be read from left to right and are ordered relative to their overall efficacy. The intervention in the top left position is ranked as best after the network meta‐analysis of direct and indirect effects. Direct comparisons are provided above the drug labels, and indirect comparisons are below. Boxes shaded green denote a statistically significant difference.

Abbreviations: ETRO, etrolizumab; FIL, filgotinib; GOL, golimumab; GUS, guselkumab; IFX, infliximab; MIR, mirikizumab; OZA, ozanimod; PLA, placebo; RIS, risankizumab; TOF, tofacitinib; UPA, upadacitinib; UST, ustekinumab; VED, vedolizumab.

In 14 RCTs reporting clinical remission data in 3411 patients naïve to advanced therapies [6, 7, 40, 41, 42, 43, 44, 46, 47, 48, 49, 50, 51, 52], again upadacitinib 30 mg o.d. ranked first and guselkumab 200 mg 4‐weekly second, with risankizumab 360 mg 8‐weekly third (Figure S4), but with no significant differences between active drugs after direct and indirect comparison (Table S11). In 12 trials reporting clinical remission data in 2091 patients previously exposed to advanced therapies [6, 7, 40, 43, 44, 45, 47, 48, 49, 50, 51, 52], again upadacitinib 30 mg o.d. ranked first, but mirikizumab 200 mg 4‐weekly ranked second, and vedolizumab 300 mg 8‐weekly third (Figure S5). After direct and indirect comparison upadacitinib 30 mg o.d. was superior to all drugs other than mirikizumab 200 mg 4‐weekly, vedolizumab 300 mg 8‐weekly and guselkumab 100 mg 8‐weekly (Table S12). Mirikizumab 200 mg 4‐weekly was superior to filgotinib 200 mg or 100 mg o.d., risankizumab 360 mg or 180 mg 8‐weekly, ustekinumab 90 mg 12‐weekly and etrolizumab 105 mg 4‐weekly. Vedolizumab 300 mg 8‐weekly was superior to filgotinib 100 mg o.d., risankizumab 360 mg 8‐weekly, ustekinumab 90 mg 12‐weekly and etrolizumab 105 mg 4‐weekly.

### 3.2 Failure to Achieve Endoscopic Improvement in Trials re‐Randomising Patients

The network plot for 15 trials re‐randomising patients [6, 7, 9, 41, 42, 43, 44, 45, 46, 47, 48, 49, 50, 51, 52], containing 6130 patients, for endoscopic improvement is provided in Figure S6. There was low heterogeneity (τ^2^ = 0.0017), but the funnel plot appeared asymmetrical (Figure S7), suggesting publication bias or other small study effects. All drugs and dosages, other than filgotinib 100 mg o.d., were superior to placebo, but upadacitinib 30 mg o.d. ranked first (RR of failure to achieve endoscopic improvement = 0.43; 95% CI 0.35–0.52, *p*‐score 0.99) (Figure 1b). Vedolizumab 300 mg 4‐weekly ranked second (RR = 0.55; 95% CI 0.43–0.69, *p*‐score 0.84) and vedolizumab 108 mg subcutaneously 2‐weekly third (RR = 0.55; 95% CI 0.42–0.72, *p*‐score 0.82). After direct and indirect comparison, upadacitinib 30 mg o.d. was superior to all drugs other than vedolizumab 300 mg 4‐weekly and vedolizumab 108 mg subcutaneously 2‐weekly (Table 2). Vedolizumab 300 mg 4‐weekly was superior to tofacitinib 5 mg b.i.d., ozanimod 1 mg o.d., golimumab 100 mg or 50 mg 4‐weekly, risankizumab 360 mg 8‐weekly, ustekinumab 90 mg 12‐weekly, etrolizumab 105 mg 4‐weekly and filgotinib 100 mg o.d. Vedolizumab 108 mg subcutaneously 2‐weekly was superior to golimumab 50 mg 4‐weekly, ustekinumab 90 mg 12‐weekly, etrolizumab 105 mg 4‐weekly and filgotinib 100 mg o.d.

**TABLE 2:** League table for failure to achieve endoscopic improvement in trials re‐randomising patients with UC.

**UPA 30 mg o.d**.						0.72 [0.57; 0.90]														0.43 [0.35; 0.52]
0.78 [0.58; 1.06]	**VED 300 mg 4‐weekly**			0.91 [0.69; 1.21]																0.55 [0.44; 0.69]
0.78 [0.56; 1.08]	0.99 [0.70; 1.40]	**VED 108 mg S/C 2‐weekly**		0.94 [0.65; 1.36]																0.55 [0.42; 0.72]
0.74 [0.57; 0.95]	0.94 [0.70; 1.25]	0.95 [0.69; 1.30]	**MIR** **200 mg 4‐weekly**																	0.58 [0.49; 0.69]
0.72 [0.56; 0.94]	0.92 [0.71; 1.20]	0.93 [0.70; 1.25]	0.98 [0.77; 1.26]	**VED 300 mg 8‐weekly**																0.59 [0.50; 0.70]
0.72 [0.55; 0.94]	0.91 [0.68; 1.23]	0.93 [0.67; 1.27]	0.98 [0.76; 1.26]	0.99 [0.77; 1.27]	**GUS 200 mg 4‐weekly**		0.96 [0.77; 1.19]													0.60 [0.50; 0.72]
0.72 [0.57; 0.90]	0.91 [0.69; 1.21]	0.92 [0.68; 1.26]	0.97 [0.77; 1.23]	0.99 [0.78; 1.25]	1.00 [0.78; 1.27]	**UPA 15 mg o.d**.														0.60 [0.51; 0.70]
0.69 [0.53; 0.89]	0.88 [0.66; 1.17]	0.89 [0.65; 1.22]	0.94 [0.73; 1.20]	0.95 [0.74; 1.21]	0.96 [0.77; 1.19]	0.96 [0.76; 1.22]	**GUS 100 mg 8‐weekly**													0.62 [0.52; 0.74]
0.69 [0.53; 0.88]	0.87 [0.66; 1.16]	0.88 [0.65; 1.20]	0.93 [0.74; 1.18]	0.95 [0.75; 1.20]	0.96 [0.75; 1.22]	0.96 [0.76; 1.20]	1.00 [0.78; 1.27]	**TOF 10 mg b.i.d**.				0.87 [0.72; 1.04]								0.63 [0.53; 0.73]
0.63 [0.48; 0.83]	0.80 [0.59; 1.08]	0.81 [0.58; 1.12]	0.85 [0.66; 1.11]	0.87 [0.67; 1.12]	0.87 [0.67; 1.14]	0.88 [0.68; 1.13]	0.91 [0.70; 1.19]	0.91 [0.71; 1.18]	**UST 90 mg 8‐weekly**								0.87 [0.70; 1.08]			0.68 [0.56; 0.83]
0.60 [0.45; 0.78]	0.76 [0.56; 1.03]	0.77 [0.55; 1.07]	0.81 [0.62; 1.05]	0.82 [0.63; 1.07]	0.83 [0.64; 1.08]	0.83 [0.65; 1.07]	0.87 [0.66; 1.13]	0.87 [0.67; 1.12]	0.95 [0.72; 1.25]	**RIS 180 mg 8‐weekly**					0.95 [0.76; 1.19]					0.72 [0.59; 0.88]
0.60 [0.46; 0.76]	0.76 [0.58; 1.00]	0.77 [0.57; 1.04]	0.81 [0.64; 1.02]	0.82 [0.65; 1.03]	0.83 [0.65; 1.05]	0.83 [0.67; 1.04]	0.87 [0.68; 1.09]	0.87 [0.69; 1.08]	0.95 [0.74; 1.22]	1.00 [0.78; 1.28]	**FIL 200 mg o.d**.								0.81 [0.68; 0.95]	0.72 [0.62; 0.84]
0.59 [0.47; 0.76]	0.76 [0.58; 0.99]	0.77 [0.57; 1.04]	0.81 [0.64; 1.01]	0.82 [0.66; 1.03]	0.83 [0.66; 1.05]	0.83 [0.67; 1.03]	0.86 [0.69; 1.09]	0.87 [0.72; 1.04]	0.95 [0.74; 1.21]	1.00 [0.78; 1.27]	1.00 [0.81; 1.23]	**TOF 5 mg b.i.d**.								0.72 [0.62; 0.83]
0.58 [0.45; 0.75]	0.74 [0.56; 0.98]	0.75 [0.55; 1.02]	0.79 [0.62; 1.00]	0.80 [0.63; 1.01]	0.81 [0.63; 1.03]	0.81 [0.64; 1.02]	0.84 [0.66; 1.07]	0.85 [0.67; 1.06]	0.93 [0.72; 1.19]	0.97 [0.75; 1.26]	0.98 [0.78; 1.22]	0.98 [0.78; 1.21]	**OZA 1 mg o.d**.							0.74 [0.63; 0.87]
0.58 [0.45; 0.75]	0.74 [0.56; 0.98]	0.75 [0.55; 1.02]	0.79 [0.62; 1.00]	0.80 [0.63; 1.02]	0.81 [0.63; 1.03]	0.81 [0.64; 1.02]	0.84 [0.66; 1.08]	0.84 [0.67; 1.07]	0.92 [0.71; 1.20]	0.97 [0.75; 1.26]	0.97 [0.77; 1.22]	0.97 [0.78; 1.22]	1.00 [0.79; 1.26]	**GOL 100 mg 4‐weekly**		0.99 [0.81; 1.21]				0.74 [0.63; 0.88]
0.57 [0.43; 0.74]	0.72 [0.54; 0.97]	0.73 [0.53; 1.01]	0.77 [0.60; 1.00]	0.78 [0.61; 1.01]	0.79 [0.61; 1.03]	0.79 [0.62; 1.01]	0.83 [0.64; 1.07]	0.83 [0.65; 1.06]	0.91 [0.69; 1.19]	0.95 [0.76; 1.19]	0.95 [0.75; 1.22]	0.95 [0.75; 1.21]	0.98 [0.76; 1.26]	0.98 [0.76; 1.26]	**RIS 360 mg 8‐weekly**					0.76 [0.63; 0.91]
0.55 [0.42; 0.71]	0.70 [0.52; 0.93]	0.71 [0.51; 0.97]	0.75 [0.58; 0.96]	0.76 [0.59; 0.97]	0.76 [0.59; 0.99]	0.77 [0.60; 0.97]	0.80 [0.62; 1.03]	0.80 [0.63; 1.02]	0.87 [0.67; 1.14]	0.92 [0.71; 1.20]	0.92 [0.73; 1.17]	0.92 [0.73; 1.16]	0.94 [0.74; 1.20]	0.95 [0.78; 1.16]	0.97 [0.74; 1.25]	**GOL 50 mg 4‐weekly**				0.80 [0.67; 0.96]
0.54 [0.42; 0.71]	0.69 [0.52; 0.93]	0.70 [0.51; 0.96]	0.74 [0.57; 0.95]	0.75 [0.59; 0.96]	0.76 [0.59; 0.98]	0.76 [0.60; 0.97]	0.79 [0.61; 1.02]	0.79 [0.62; 1.01]	0.87 [0.70; 1.08]	0.91 [0.70; 1.19]	0.91 [0.72; 1.16]	0.91 [0.72; 1.15]	0.94 [0.73; 1.19]	0.94 [0.73; 1.20]	0.96 [0.74; 1.24]	0.99 [0.77; 1.28]	**UST 90 mg 12‐weekly**			0.79 [0.66; 0.95]
0.53 [0.42; 0.67]	0.67 [0.52; 0.88]	0.68 [0.51; 0.91]	0.72 [0.58; 0.89]	0.73 [0.59; 0.90]	0.73 [0.59; 0.92]	0.74 [0.60; 0.91]	0.77 [0.61; 0.96]	0.77 [0.62; 0.95]	0.84 [0.66; 1.07]	0.88 [0.70; 1.12]	0.89 [0.72; 1.08]	0.89 [0.73; 1.08]	0.91 [0.74; 1.12]	0.91 [0.73; 1.13]	0.93 [0.74; 1.17]	0.96 [0.77; 1.20]	0.97 [0.78; 1.21]	**ETRO 105 mg 4‐weekly**		0.81 [0.71; 0.93]
0.48 [0.38; 0.61]	0.61 [0.47; 0.80]	0.62 [0.46; 0.83]	0.65 [0.52; 0.81]	0.66 [0.53; 0.82]	0.67 [0.53; 0.84]	0.67 [0.54; 0.83]	0.70 [0.56; 0.87]	0.70 [0.57; 0.86]	0.77 [0.60; 0.97]	0.81 [0.63; 1.02]	0.81 [0.68; 0.95]	0.81 [0.66; 0.98]	0.83 [0.67; 1.02]	0.83 [0.67; 1.03]	0.85 [0.67; 1.07]	0.88 [0.70; 1.09]	0.88 [0.71; 1.11]	0.91 [0.75; 1.10]	**FIL 100 mg o.d**.	0.89 [0.78; 1.02]
0.43 [0.35; 0.52]	0.55 [0.43; 0.69]	0.55 [0.42; 0.72]	0.58 [0.49; 0.69]	0.59 [0.50; 0.70]	0.60 [0.50; 0.72]	0.60 [0.51; 0.70]	0.62 [0.52; 0.74]	0.63 [0.53; 0.73]	0.68 [0.56; 0.83]	0.72 [0.59; 0.88]	0.72 [0.62; 0.84]	0.72 [0.62; 0.83]	0.74 [0.63; 0.87]	0.74 [0.63; 0.88]	0.76 [0.63; 0.91]	0.78 [0.65; 0.94]	0.79 [0.66; 0.95]	0.81 [0.71; 0.93]	0.89 [0.78; 1.02]	**PLA**

*Note:* Relative risk with 95% confidence intervals in parentheses. Comparisons, column versus row, should be read from left to right and are ordered relative to their overall efficacy. The intervention in the top left position is ranked as best after the network meta‐analysis of direct and indirect effects. Direct comparisons are provided above the drug labels, and indirect comparisons are below. Boxes shaded green denote a statistically significant difference.

Abbreviations: ETRO, etrolizumab; FIL, filgotinib; GOL, golimumab; GUS, guselkumab; IFX, infliximab; MIR, mirikizumab; OZA, ozanimod; PLA, placebo; RIS, risankizumab; TOF, tofacitinib; UPA, upadacitinib; UST, ustekinumab; VED, vedolizumab.

In 10 RCTs reporting endoscopic improvement data in 2505 patients naïve to advanced therapies [7, 41, 42, 43, 46, 47, 48, 49, 50, 51], risankizumab 360 mg 8‐weekly ranked first, upadacitinib 30 mg o.d. second and guselkumab 200 mg 4‐weekly third (Figure S8), but with no significant differences between active drugs after direct and indirect comparison (Table S13). In eight trials reporting endoscopic improvement data in 1929 patients previously exposed to advanced therapies [7, 43, 45, 47, 48, 49, 50, 51], upadacitinib 30 mg o.d. ranked first, with vedolizumab 300 mg 8‐weekly second and guselkumab 100 mg 8‐weekly third (Figure S9), although vedolizumab 300 mg 8‐weekly was not superior to placebo. After direct and indirect comparison upadacitinib 30 mg o.d. was superior to all drugs other than vedolizumab 300 mg 8‐weekly and guselkumab 100 mg 8‐weekly. Guselkumab 100 mg 8‐weekly was superior to etrolizumab 105 mg 4‐weekly, filgotinib 200 mg or 100 mg o.d., risankizumab 360 mg 8‐weekly and ustekinumab 90 mg 12‐weekly (Table S14).

### 3.3 Failure to Achieve Endoscopic Remission in Trials re‐Randomising Patients

The network plot for 10 trials re‐randomising patients [6, 9, 41, 44, 45, 46, 48, 49, 50, 51], containing 4460 patients, for endoscopic remission is provided in Figure S10. There was no heterogeneity (τ^2^ = 0), and the funnel plot appeared symmetrical (Figure S11). All drugs and dosages, other than golimumab 100 mg 4‐weekly, risankizumab 180 mg 8‐weekly and filgotinib 100 mg o.d., were superior to placebo, but vedolizumab 300 mg 4‐weekly ranked first (RR of failure to achieve endoscopic remission = 0.73; 95% CI 0.64–0.84, *p*‐score 0.92) (Figure 1c). Upadacitinib 30 mg o.d. ranked second (RR = 0.76; 95% CI 0.70–0.83, *p*‐score 0.84) and guselkumab 100 mg 8‐weekly third (RR = 0.77; 95% CI 0.68–0.87, *p*‐score 0.81). After direct and indirect comparison, both vedolizumab 300 mg 4‐weekly and upadacitinib 30 mg o.d. were superior to tofacitinib 10 mg or 5 mg b.i.d., risankizumab 360 mg or 180 mg 8‐weekly, golimumab 100 mg 4‐weekly and filgotinib 200 mg or 100 mg o.d. (Table 3). Guselkumab 100 mg 8‐weekly was superior to tofacitinib 5 mg b.i.d. and filgotinib 200 mg or 100 mg o.d.

**TABLE 3:** League table for failure to achieve endoscopic remission in trials re‐randomising patients with UC.

**VED 300 mg 4‐weekly**				0.93 [0.79; 1.10]												0.73 [0.64; 0.83]
0.95 [0.81; 1.12]	**UPA 30 mg o.d**.				0.95 [0.85; 1.07]											0.76 [0.70; 0.83]
0.95 [0.79; 1.13]	0.99 [0.85; 1.15]	**GUS 100 mg 8‐weekly**	0.99 [0.85; 1.14]													0.77 [0.68; 0.87]
0.93 [0.78; 1.12]	0.98 [0.84; 1.13]	0.99 [0.85; 1.14]	**GUS 200 mg 4‐weekly**													0.78 [0.70; 0.88]
0.92 [0.78; 1.08]	0.96 [0.84; 1.10]	0.97 [0.83; 1.14]	0.99 [0.84; 1.15]	**VED 300 mg 8‐weekly**		1.02 [0.83; 1.25]										0.79 [0.72; 0.88]
0.91 [0.78; 1.07]	0.95 [0.85; 1.07]	0.96 [0.83; 1.11]	0.98 [0.85; 1.13]	0.99 [0.87; 1.13]	**UPA 15 mg o.d**.											0.80 [0.74; 0.87]
0.91 [0.75; 1.11]	0.96 [0.80; 1.14]	0.96 [0.80; 1.17]	0.98 [0.81; 1.18]	0.99 [0.84; 1.17]	1.00 [0.84; 1.19]	**VED 108 mg S/C 2‐weekly**										0.81 [0.69; 0.95]
0.85 [0.72; 1.01]	0.89 [0.78; 1.03]	0.90 [0.77; 1.06]	0.91 [0.78; 1.07]	0.93 [0.80; 1.08]	0.94 [0.82; 1.07]	0.93 [0.77; 1.13]	**GOL 50 mg 4‐weekly**					0.95 [0.84; 1.08]				0.86 [0.77; 0.96]
0.85 [0.72; 1.00]	0.89 [0.79; 1.01]	0.90 [0.77; 1.05]	0.91 [0.79; 1.06]	0.93 [0.81; 1.06]	0.93 [0.83; 1.06]	0.93 [0.78; 1.11]	1.00 [0.87; 1.15]	**ETRO 105 mg 4‐weekly**								0.86 [0.78; 0.94]
0.84 [0.72; 0.98]	0.88 [0.79; 0.99]	0.89 [0.77; 1.02]	0.90 [0.79; 1.03]	0.92 [0.81; 1.04]	0.92 [0.83; 1.03]	0.92 [0.78; 1.09]	0.99 [0.87; 1.12]	0.99 [0.88; 1.11]	**TOF 10 mg b.i.d**.		0.98 [0.90; 1.06]					0.87 [0.81; 0.93]
0.82 [0.69; 0.97]	0.86 [0.75; 0.98]	0.87 [0.74; 1.02]	0.88 [0.75; 1.03]	0.89 [0.77; 1.03]	0.90 [0.79; 1.03]	0.90 [0.75; 1.08]	0.96 [0.83; 1.12]	0.96 [0.84; 1.11]	0.98 [0.86; 1.10]	**RIS 360 mg 8‐weekly**			0.98 [0.88; 1.10]			0.89 [0.80; 0.98]
0.82 [0.71; 0.95]	0.86 [0.77; 0.96]	0.87 [0.76; 0.99]	0.88 [0.77; 1.01]	0.89 [0.79; 1.01]	0.90 [0.81; 1.00]	0.90 [0.76; 1.06]	0.96 [0.85; 1.09]	0.96 [0.86; 1.08]	0.98 [0.90; 1.06]	1.00 [0.89; 1.13]	**TOF 5 mg b.i.d**.					0.89 [0.83; 0.95]
0.81 [0.68; 0.96]	0.85 [0.74; 0.97]	0.86 [0.73; 1.00]	0.87 [0.74; 1.01]	0.88 [0.76; 1.02]	0.89 [0.78; 1.01]	0.89 [0.74; 1.07]	0.95 [0.84; 1.08]	0.95 [0.83; 1.09]	0.96 [0.85; 1.09]	0.99 [0.85; 1.14]	0.99 [0.87; 1.11]	**GOL 100 mg 4‐weekly**				0.90 [0.81; 1.00]
0.81 [0.68; 0.95]	0.85 [0.74; 0.97]	0.85 [0.73; 1.00]	0.87 [0.74; 1.01]	0.88 [0.76; 1.01]	0.89 [0.78; 1.01]	0.89 [0.74; 1.06]	0.95 [0.82; 1.10]	0.95 [0.83; 1.09]	0.96 [0.85; 1.08]	0.98 [0.88; 1.10]	0.98 [0.87; 1.11]	1.00 [0.86; 1.15]	**RIS 180 mg 8‐weekly**			0.90 [0.82; 1.00]
0.80 [0.69; 0.94]	0.84 [0.75; 0.94]	0.85 [0.74; 0.98]	0.86 [0.75; 0.99]	0.87 [0.77; 0.99]	0.88 [0.79; 0.98]	0.88 [0.75; 1.04]	0.94 [0.83; 1.07]	0.94 [0.84; 1.06]	0.95 [0.87; 1.05]	0.98 [0.87; 1.11]	0.98 [0.89; 1.08]	0.99 [0.88; 1.12]	1.00 [0.88; 1.12]	**FIL 200 mg o.d**.	0.97 [0.90; 1.05]	0.91 [0.85; 0.97]
0.78 [0.67; 0.91]	0.82 [0.73; 0.91]	0.83 [0.72; 0.95]	0.84 [0.73; 0.96]	0.85 [0.75; 0.96]	0.86 [0.77; 0.95]	0.86 [0.72; 1.01]	0.92 [0.80; 1.04]	0.92 [0.82; 1.03]	0.93 [0.84; 1.02]	0.95 [0.84; 1.07]	0.95 [0.87; 1.04]	0.96 [0.85; 1.09]	0.97 [0.86; 1.09]	0.97 [0.90; 1.05]	**FIL 100 mg o.d**.	0.94 [0.87; 1.00]
0.73 [0.64; 0.84]	0.76 [0.70; 0.83]	0.77 [0.68; 0.87]	0.78 [0.70; 0.88]	0.79 [0.72; 0.88]	0.80 [0.74; 0.87]	0.80 [0.69; 0.93]	0.86 [0.77; 0.96]	0.86 [0.78; 0.94]	0.87 [0.81; 0.93]	0.89 [0.80; 0.98]	0.89 [0.83; 0.95]	0.90 [0.81; 1.00]	0.90 [0.82; 1.00]	0.91 [0.85; 0.97]	0.94 [0.87; 1.00]	**PLA**

*Note:* Relative risk with 95% confidence intervals in parentheses. Comparisons, column versus row, should be read from left to right and are ordered relative to their overall efficacy. The intervention in the top left position is ranked as best after the network meta‐analysis of direct and indirect effects. Direct comparisons are provided above the drug labels, and indirect comparisons are below. Boxes shaded green denote a statistically significant difference.

Abbreviations: ETRO, etrolizumab; FIL, filgotinib; GOL, golimumab; GUS, guselkumab; MIR, mirikizumab; PLA, placebo; RIS, risankizumab; TOF, tofacitinib; UPA, upadacitinib; UST, ustekinumab; VED, vedolizumab.

In five RCTs reporting endoscopic remission data in 1469 patients naïve to advanced therapies [41, 46, 48, 49, 51], risankizumab 360 mg 8‐weekly ranked first, guselkumab 200 mg 4‐weekly second and upadacitinib 30 mg o.d. third (Figure S12). Risankizumab 360 mg 8‐weekly was superior to golimumab 100 mg 4‐weekly, but there were no other significant differences between active drugs after direct and indirect comparison (Table S15). In four trials reporting endoscopic remission data in 1219 patients previously exposed to advanced therapies [45, 48, 49, 51], guselkumab 100 mg 8‐weekly ranked first, with upadacitinib 30 mg o.d. second and upadacitinib 15 mg o.d. third (Figure S13). After direct and indirect comparison, guselkumab 100 mg 8‐weekly and upadacitinib 30 mg o.d. were superior to risankizumab 360 mg or 180 mg 8‐weekly and upadacitinib 15 mg o.d. was superior to risankizumab 360 mg 8‐weekly (Table S16).

### 3.4 Failure to Achieve Corticosteroid‐Free Remission in Trials re‐Randomising Patients

The network plot for 15 trials re‐randomising patients [6, 7, 9, 40, 41, 42, 43, 44, 45, 46, 50, 51, 52, 53, 54], containing 2606 patients, for corticosteroid‐free remission is provided in Figure S14. There was no heterogeneity (τ^2^ = 0), but the funnel plot appeared asymmetrical (Figure S15), suggesting publication bias or other small study effects. All drugs and dosages, other than golimumab 100 mg or 50 mg 8‐weekly, etrolizumab 105 mg 4‐weekly and filgotinib 100 mg o.d., were superior to placebo, but guselkumab 200 mg 4‐weekly ranked first (RR of failure to achieve corticosteroid‐free remission = 0.40; 95% CI 0.28–0.55, *p*‐score 0.95) (Figure 1d). Ustekinumab 90 mg 8‐weekly ranked second (RR = 0.41; 95% CI 0.27–0.62, *p*‐score 0.94) and upadacitinib 30 mg o.d. third (RR = 0.44; 95% CI 0.33–0.59, *p*‐score 0.92). After direct and indirect comparison, guselkumab 200 mg 4‐weekly was superior to all drugs other than ustekinumab 90 mg 8‐weekly or 12‐weekly and upadacitinib 30 mg or 15 mg o.d. (Table 4). Ustekinumab 90 mg 8‐weekly and upadacitinib 30 mg o.d. were superior to all drugs other than upadacitinib 15 mg o.d., guselkumab 100 mg 8‐weekly, ustekinumab 90 mg 12‐weekly and vedolizumab 300 mg 4‐weekly.

**TABLE 4:** League table for failure to achieve corticosteroid‐free remission in trials re‐randomising patients with UC.

**GUS 200 mg 4‐weekly**				0.66 [0.45; 0.97]															0.40 [0.28; 0.55]
0.97 [0.57; 1.65]	**UST 90 mg 8‐weekly**				0.69 [0.43; 1.13]														0.41 [0.27; 0.62]
0.90 [0.58; 1.41]	0.93 [0.55; 1.55]	**UPA 30 mg o.d**.	0.75 [0.52; 1.09]																0.44 [0.33; 0.59]
0.68 [0.45; 1.03]	0.70 [0.43; 1.14]	0.75 [0.52; 1.09]	**UPA 15 mg o.d**.																0.58 [0.45; 0.76]
0.66 [0.45; 0.97]	0.68 [0.42; 1.10]	0.73 [0.50; 1.07]	0.97 [0.68; 1.38]	**GUS 100 mg 8‐weekly**															0.60 [0.47; 0.76]
0.67 [0.41; 1.09]	0.69 [0.43; 1.13]	0.75 [0.47; 1.19]	0.99 [0.64; 1.54]	1.02 [0.67; 1.57]	**UST 90 mg 12‐weekly**														0.59 [0.41; 0.84]
0.62 [0.42; 0.93]	0.64 [0.40; 1.03]	0.69 [0.48; 1.01]	0.92 [0.65; 1.30]	0.95 [0.68; 1.32]	0.93 [0.61; 1.42]	**VED 300 mg 4‐weekly**				0.80 [0.61; 1.04]									0.64 [0.51; 0.80]
0.57 [0.39; 0.84]	0.59 [0.37; 0.93]	0.64 [0.45; 0.91]	0.85 [0.62; 1.16]	0.87 [0.65; 1.18]	0.85 [0.57; 1.28]	0.92 [0.69; 1.23]	**MIR 200 mg 4‐weekly**												0.69 [0.57; 0.83]
0.51 [0.36; 0.73]	0.53 [0.34; 0.82]	0.57 [0.41; 0.79]	0.75 [0.56; 1.01]	0.78 [0.59; 1.02]	0.76 [0.52; 1.11]	0.82 [0.63; 1.07]	0.89 [0.71; 1.12]	**FIL 200 mg o.d**.										0.84 [0.72; 0.98]	0.77 [0.68; 0.89]
0.51 [0.35; 0.74]	0.53 [0.33; 0.83]	0.57 [0.40; 0.81]	0.76 [0.55; 1.03]	0.78 [0.58; 1.05]	0.76 [0.51; 1.14]	0.82 [0.61; 1.09]	0.89 [0.69; 1.15]	1.00 [0.80; 1.26]	**IFX 120 mg 2‐weekly**										0.77 [0.65; 0.93]
0.51 [0.35; 0.73]	0.52 [0.33; 0.81]	0.56 [0.40; 0.79]	0.75 [0.55; 1.01]	0.77 [0.58; 1.02]	0.75 [0.51; 1.11]	0.81 [0.63; 1.04]	0.88 [0.69; 1.12]	0.99 [0.81; 1.21]	0.99 [0.78; 1.25]	**VED 300 mg 8‐weekly**	0.97 [0.71; 1.35]								0.78 [0.67; 0.91]
0.49 [0.33; 0.73]	0.51 [0.32; 0.81]	0.55 [0.38; 0.79]	0.73 [0.52; 1.02]	0.75 [0.55; 1.03]	0.73 [0.48; 1.11]	0.79 [0.58; 1.07]	0.86 [0.65; 1.14]	0.97 [0.75; 1.24]	0.97 [0.73; 1.27]	0.98 [0.77; 1.25]	**VED 108 mg S/C 2‐weekly**								0.80 [0.65; 0.99]
0.49 [0.34; 0.70]	0.50 [0.32; 0.78]	0.54 [0.39; 0.75]	0.72 [0.54; 0.96]	0.74 [0.56; 0.97]	0.72 [0.49; 1.06]	0.78 [0.60; 1.02]	0.85 [0.68; 1.07]	0.95 [0.79; 1.16]	0.95 [0.76; 1.20]	0.96 [0.79; 1.18]	0.99 [0.77; 1.27]	**TOF 5 mg b.i.d**.	1.00 [0.84; 1.19]						0.81 [0.71; 0.93]
0.49 [0.34; 0.70]	0.50 [0.32; 0.78]	0.54 [0.39; 0.75]	0.72 [0.53; 0.97]	0.74 [0.56; 0.98]	0.72 [0.49; 1.06]	0.78 [0.60; 1.02]	0.85 [0.67; 1.07]	0.95 [0.78; 1.16]	0.95 [0.75; 1.20]	0.96 [0.78; 1.19]	0.99 [0.76; 1.27]	1.00 [0.84; 1.19]	**TOF 10 mg b.i.d**.						0.81 [0.70; 0.94]
0.45 [0.31; 0.66]	0.47 [0.30; 0.73]	0.50 [0.36; 0.71]	0.67 [0.49; 0.91]	0.69 [0.51; 0.92]	0.67 [0.45; 1.00]	0.73 [0.55; 0.97]	0.79 [0.62; 1.02]	0.89 [0.71; 1.11]	0.89 [0.69; 1.14]	0.90 [0.72; 1.13]	0.92 [0.70; 1.20]	0.93 [0.75; 1.16]	0.93 [0.75; 1.17]	**GOL 50 mg 4‐weekly**			0.93 [0.78; 1.12]		0.88 [0.74; 1.04]
0.44 [0.31; 0.62]	0.45 [0.29; 0.70]	0.49 [0.35; 0.67]	0.65 [0.49; 0.86]	0.67 [0.51; 0.87]	0.65 [0.45; 0.95]	0.70 [0.55; 0.90]	0.76 [0.62; 0.95]	0.86 [0.72; 1.02]	0.86 [0.70; 1.06]	0.87 [0.72; 1.04]	0.89 [0.70; 1.12]	0.90 [0.75; 1.07]	0.90 [0.75; 1.08]	0.97 [0.79; 1.18]	**ETRO 105 mg 4‐weekly**				0.90 [0.81; 1.00]
0.44 [0.31; 0.62]	0.45 [0.29; 0.69]	0.49 [0.36; 0.67]	0.65 [0.49; 0.85]	0.67 [0.52; 0.86]	0.65 [0.45; 0.94]	0.70 [0.55; 0.90]	0.76 [0.62; 0.94]	0.86 [0.73; 1.01]	0.86 [0.70; 1.05]	0.87 [0.73; 1.04]	0.89 [0.71; 1.12]	0.90 [0.76; 1.06]	0.90 [0.76; 1.07]	0.97 [0.80; 1.17]	1.00 [0.87; 1.15]	**OZA 1 mg o.d**.			0.90 [0.82; 0.99]
0.43 [0.30; 0.62]	0.44 [0.28; 0.69]	0.48 [0.34; 0.67]	0.64 [0.47; 0.86]	0.65 [0.49; 0.87]	0.64 [0.43; 0.94]	0.69 [0.53; 0.91]	0.75 [0.59; 0.95]	0.84 [0.69; 1.03]	0.84 [0.66; 1.07]	0.85 [0.69; 1.06]	0.87 [0.67; 1.13]	0.88 [0.72; 1.09]	0.89 [0.72; 1.09]	0.95 [0.79; 1.14]	0.98 [0.81; 1.18]	0.98 [0.82; 1.17]	**GOL 100 mg 4‐weekly**		0.92 [0.79; 1.07]
0.43 [0.30; 0.61]	0.44 [0.29; 0.68]	0.48 [0.35; 0.66]	0.64 [0.48; 0.84]	0.65 [0.50; 0.85]	0.64 [0.44; 0.93]	0.69 [0.54; 0.88]	0.75 [0.61; 0.92]	0.84 [0.72; 0.98]	0.84 [0.68; 1.03]	0.85 [0.71; 1.02]	0.87 [0.69; 1.10]	0.88 [0.74; 1.05]	0.88 [0.74; 1.06]	0.95 [0.78; 1.16]	0.98 [0.85; 1.14]	0.98 [0.85; 1.13]	1.00 [0.83; 1.20]	**FIL 100 mg o.d**.	0.92 [0.83; 1.02]
0.40 [0.28; 0.55]	0.41 [0.27; 0.62]	0.44 [0.33; 0.59]	0.58 [0.45; 0.76]	0.60 [0.47; 0.76]	0.59 [0.41; 0.84]	0.63 [0.51; 0.79]	0.69 [0.57; 0.83]	0.77 [0.68; 0.89]	0.77 [0.65; 0.93]	0.78 [0.67; 0.91]	0.80 [0.65; 0.99]	0.81 [0.71; 0.93]	0.81 [0.70; 0.94]	0.87 [0.73; 1.03]	0.90 [0.81; 1.00]	0.90 [0.82; 0.99]	0.92 [0.79; 1.07]	0.92 [0.83; 1.02]	**PLA**

*Note:* Relative risk with 95% confidence intervals in parentheses. Comparisons, column versus row, should be read from left to right and are ordered relative to their overall efficacy. The intervention in the top left position is ranked as best after the network meta‐analysis of direct and indirect effects. Direct comparisons are provided above the drug labels, and indirect comparisons are below. Boxes shaded green denote a statistically significant difference.

Abbreviations: ETRO, etrolizumab; FIL, filgotinib; GOL, golimumab; GUS, guselkumab; IFX, infliximab; MIR, mirikizumab; OZA, ozanimod; PLA, placebo; RIS, risankizumab; TOF, tofacitinib; UPA, upadacitinib; UST, ustekinumab; VED, vedolizumab.

In seven RCTs reporting corticosteroid‐free remission data in 770 patients naïve to advanced therapies [40, 41, 42, 43, 46, 50, 51], upadacitinib 30 mg o.d. ranked first and filgotinib 200 mg o.d. second, with vedolizumab 300 mg 8‐weekly third (Figure S16), but none of the drugs were superior to placebo and there were no significant differences between active drugs after direct and indirect comparison (Table S17). In five trials reporting corticosteroid‐free remission data in 428 patients previously exposed to advanced therapies [40, 43, 45, 50, 51], upadacitinib 30 mg o.d. ranked first, with vedolizumab 300 mg 8‐weekly second and upadacitinib 15 mg o.d. third (Figure S17). However, vedolizumab 300 mg 8‐weekly was not superior to placebo. After direct and indirect comparison upadacitinib 30 mg o.d. was superior to all drugs other than vedolizumab 300 mg 8‐weekly and infliximab 120 mg subcutaneously 2‐weekly and upadacitinib 15 mg o.d. was superior to filgotinib 100 mg o.d. (Table S18).

### 3.5 Failure to Achieve Histological Endpoints in Trials re‐Randomising Patients

There were five RCTs re‐randomising patients [7, 40, 48, 49, 51], containing 2758 patients, providing information on histological‐endoscopic mucosal improvement, five trials [47, 48, 50, 51, 52], containing 2801 patients, providing information on histological‐endoscopic mucosal remission, and seven RCTs [44, 45, 46, 48, 49, 50, 52], containing 2706 patients, providing information on histological remission. In terms of histological‐endoscopic mucosal improvement, all drugs and dosages studied were superior to placebo, but upadacitinib 30 mg o.d. ranked first (RR of failure to achieve histological‐endoscopic mucosal improvement = 0.50; 95% CI 0.43–0.58, *p*‐score 1.00) (Figure S18). Guselkumab 200 mg 4‐weekly ranked second (RR = 0.63; 95% CI 0.54–0.73, *p*‐score 0.82) and upadacitinib 15 mg o.d. third (RR = 0.68; 95% CI 0.60–0.76, *p*‐score 0.67). After direct and indirect comparison, upadacitinib 30 mg o.d. was superior to all other drugs and dosages, and guselkumab 200 mg 4‐weekly was superior to infliximab 120 mg subcutaneously 2‐weekly and ustekinumab 90 mg 12‐weekly (Table S19). For histological‐endoscopic mucosal remission, all drugs other than risankizumab were superior to placebo, but mirikizumab 200 mg 4‐weekly ranked first (RR of failure to achieve histological‐endoscopic mucosal remission = 0.73; 95% CI 0.64–0.82, *p*‐score 0.95), followed by filgotinib 200 mg o.d. (RR = 0.77; 95% CI 0.69–0.85, *p*‐score 0.85) and upadacitinib 30 mg o.d. (RR = 0.81; 95% CI 0.75–0.88, *p*‐score 0.70) (Figure S19). After direct and indirect comparison, mirikizumab 200 mg 4‐weekly was superior to all drugs and dosages other than filgotinib 200 mg o.d., upadacitinib 30 mg o.d. and ozanimod 1 mg o.d. (Table S20). Filgotinib 200 mg o.d. was superior to filgotinib 100 mg o.d. and risankizumab 360 mg or 180 mg 8‐weekly. Upadacitinib 30 mg o.d. was superior to risankizumab 360 mg or 180 mg 8‐weekly. Finally, for histological remission, all drugs other than risankizumab or vedolizumab were superior to placebo. Guselkumab 200 mg 4‐weekly ranked first (RR = 0.54; 95% CI 0.44–0.66, *p*‐score 0.96), guselkumab 100 mg 8‐weekly second (RR = 0.56; 95% CI 0.46–0.68, *p*‐score 0.94) and filgotinib 200 mg o.d. third (RR = 0.74; 95% CI 0.65–0.83, *p*‐score 0.76) (Figure S20). Following direct and indirect comparison, guselkumab 200 mg 4‐weekly was superior to all drugs and dosages other than guselkumab 100 mg 8‐weekly, guselkumab 100 mg 8‐weekly was superior to all other drugs and dosages, and filgotinib 200 mg o.d. was superior to all drugs and dosages other than etrolizumab 105 mg 4‐weekly and ozanimod 1 mg o.d. (Table S21).

### 3.6 Failure to Achieve Clinical Remission in Trials Treating Patients Through

The network plot of the 12 treat‐through trials [5, 8, 11, 32, 33, 34, 35, 36, 37, 38, 39], containing 3771 patients, for clinical remission is provided in Figure S21. There was low heterogeneity in this analysis (τ^2^ = 0.0022), but the funnel plot appeared asymmetrical (Figure S22), suggesting publication bias or other small study effects. All drugs and dosages, other than etrolizumab 105 mg 4‐weekly, were superior to placebo, but etrasimod 2 mg o.d. ranked first (RR of failure to achieve clinical remission = 0.73; 95% CI 0.64–0.83, *p*‐score 0.88) (Figure S23). Infliximab 10 mg/kg 8‐weekly ranked second (RR = 0.76; 95% CI 0.67–0.86, *p*‐score 0.79) and vedolizumab 300 mg 8‐weekly third (RR = 0.78; 95% CI 0.67–0.90, *p*‐score 0.71). The network heat plot had no red ‘hotspots’ of inconsistency. After direct and indirect comparison, etrasimod 2 mg o.d. was superior to adalimumab 40 mg 2‐weekly (moderate confidence according to the CINeMA framework), but there were no other significant differences between active drugs (Table S22). Using the CINeMA framework to evaluate confidence in all other results for this endpoint, all direct and indirect comparisons across the network were rated as either high or moderate confidence other than the direct comparison between adalimumab 40 mg 2‐weekly and vedolizumab 300 mg 8‐weekly (Table S23).

In 10 RCTs reporting clinical remission data in 2996 patients naïve to advanced therapies [5, 11, 32, 33, 34, 35, 36, 37, 38], again etrasimod 2 mg o.d. ranked first, with vedolizumab 300 mg 8‐weekly second and infliximab 10 mg/kg 8‐weekly third (Figure S24). After direct and indirect comparison, etrasimod 2 mg o.d. was superior to all drugs other than vedolizumab 300 mg 8‐weekly and infliximab 10 mg/kg 8‐weekly, but there were no other significant differences between active drugs (Table S24). In three trials reporting clinical remission data in 489 patients previously exposed to advanced therapies [11, 32, 38], etrasimod 2 mg o.d. was the only drug superior to placebo (Figure S25). After direct and indirect comparison, there were no significant differences between active drugs (Table S25).

### 3.7 Failure to Achieve Endoscopic Improvement in Trials Treating Patients Through

The network plot for the 11 treat‐through trials [5, 8, 11, 32, 33, 34, 35, 36, 37, 38], containing 3641 patients, for endoscopic improvement is provided in Figure S26. There was no heterogeneity (τ^2^ = 0), and the funnel plot appeared asymmetrical (Figure S27), suggesting publication bias or other small study effects. All drugs and dosages were superior to placebo, but infliximab 10 mg/kg 8‐weekly ranked first (RR of failure to achieve endoscopic improvement = 0.64; 95% CI 0.56–0.74, *p*‐score 0.94) (Figure S28). Etrasimod 2 mg o.d. ranked second (RR = 0.70; 95% CI 0.63–0.78, *p*‐score 0.76) and vedolizumab 300 mg 8‐weekly third (RR = 0.73; 95% CI 0.65–0.83, *p*‐score 0.63). The network heat plot had no red ‘hotspots’ of inconsistency. After direct and indirect comparison, infliximab 10 mg/kg 8‐weekly was superior to etrolizumab 105 mg 4‐weekly and adalimumab 40 mg 2‐weekly (Table S26). Etrasimod 2 mg o.d. and vedolizumab 300 mg 8‐weekly were superior to adalimumab 40 mg 2‐weekly.

In 10 RCTs reporting endoscopic improvement data in 2996 patients naïve to advanced therapies [5, 11, 32, 33, 34, 35, 36, 37, 38], infliximab 10 mg/kg 8‐weekly ranked first, vedolizumab 300 mg 8‐weekly second and etrasimod 2 mg o.d. third (Figure S29). Again, after direct and indirect comparison, infliximab 10 mg/kg 8‐weekly was superior to etrolizumab 105 mg 4‐weekly and adalimumab 40 mg 2‐weekly (Table S27). Vedolizumab 300 mg 8‐weekly and etrasimod 2 mg o.d. were both superior to adalimumab 40 mg 2‐weekly. In three trials reporting endoscopic improvement data in 489 patients previously exposed to advanced therapies [11, 32, 38], etrasimod 2 mg o.d. was the only drug superior to placebo (Figure S30), and after direct and indirect comparison was also superior to adalimumab 40 mg 2‐weekly (Table S28).

### 3.8 Failure to Achieve Corticosteroid‐Free Remission in Trials Treating Patients Through

The network plot for the nine treat‐through trials [5, 11, 32, 33, 34, 35, 37, 38], containing 1743 patients, for corticosteroid‐free remission is provided in Figure S31. There was low heterogeneity (τ^2^ = 0.0019). All drugs and dosages, other than adalimumab 40 mg 2‐weekly and vedolizumab 300 mg 8‐weekly, were superior to placebo, but etrasimod 2 mg o.d. ranked first (RR of failure to achieve corticosteroid‐free remission = 0.75; 95% CI 0.62–0.90, *p*‐score 0.91) (Figure S32). Infliximab 5 mg/kg 8‐weekly ranked second (RR = 0.82; 95% CI 0.75–0.90, *p*‐score 0.74) and etrolizumab 105 mg 4‐weekly third (RR = 0.84; 95% CI 0.70–1.01, *p*‐score 0.65), although the latter was not superior to placebo. The network heat plot had no red ‘hotspots’ of inconsistency. After direct and indirect comparison, both etrasimod 2 mg o.d. and infliximab 5 mg/kg 8‐weekly were superior to adalimumab 40 mg 2‐weekly and vedolizumab 300 mg 8‐weekly (Table S29).

In eight RCTs reporting corticosteroid‐free remission data in 1342 patients naïve to advanced therapies [5, 11, 32, 33, 34, 35, 37], infliximab 5 mg/kg 8‐weekly ranked first and infliximab 10 mg/kg 8‐weekly ranked second and were the only active drugs superior to placebo (Figure S33). However, there were no significant differences between active drugs after direct and indirect comparison (Table S30). In two trials reporting corticosteroid‐free remission data in 250 patients previously exposed to advanced therapies [11, 32], only adalimumab 40 mg 2‐weekly and vedolizumab 300 mg 8‐weekly were assessed and neither was superior to placebo or, after direct and indirect comparison, to each other (Table S31).

## 4 Discussion

We conducted a contemporaneous systematic review and network meta‐analysis of 28 trials of biological therapies or small molecules as maintenance therapy in UC. In re‐randomised trials in all patients, irrespective of previous exposure to advanced therapies, upadacitinib 30 mg o.d. demonstrated the highest efficacy for clinical remission. Upadacitinib 30 mg o.d. appeared superior to almost all other drugs studied. Most comparisons across this network were rated as either high or moderate confidence. Upadacitinib remained the highest ranked drug for clinical remission in both patients naïve and exposed to advanced therapies, although it was only superior to other active drugs in patients previously exposed to advanced therapies. Upadacitinib 30 mg o.d. also ranked highest in all patients for endoscopic improvement and in patients exposed to advanced therapies. In terms of endoscopic remission, vedolizumab 300 mg 4‐weekly ranked first in all patients. Finally, for corticosteroid‐free remission, guselkumab 200 mg 4‐weekly ranked first in all patients. When we evaluated histological endpoints, upadacitinib 30 mg o.d. ranked first for histological‐endoscopic mucosal improvement and was superior to all other drugs studied; mirikizumab 200 mg 4‐weekly ranked first for histological‐endoscopic mucosal remission, and guselkumab 200 mg 4‐weekly ranked highest for histological remission and was superior to all other drugs. In terms of safety, all drugs were safe and well tolerated. Ozanimod 1 mg o.d. was associated with a higher likelihood of treatment‐emergent adverse events, and guselkumab 200 mg 4‐weekly with a higher likelihood of serious adverse events than placebo, but there were no other significant differences.

In treat‐through trials, etrasimod 2 mg o.d. ranked highest for clinical remission in all patients. All comparisons across this network were rated as either high or moderate confidence. The ranking of etrasimod 2 mg o.d. was consistent in both patients naïve to or exposed to advanced therapies. For endoscopic improvement, infliximab 10 mg/kg 8‐weekly ranked first in all patients. Infliximab 10 mg/kg 8‐weekly ranked first in patients naïve to advanced therapies, and etrasimod 2 mg o.d. ranked first in patients previously exposed. For corticosteroid‐free remission, again etrasimod 2 mg o.d. ranked first in all patients. In patients exposed to advanced therapies, no drug was superior to placebo. The trial of etrasimod 2 mg o.d. did not provide data in either of these analyses. In terms of safety, etrasimod 2 mg o.d. was associated with a higher likelihood of treatment‐emergent adverse events than placebo, but there were no other significant differences.

Our search strategy included multiple databases and supplementary sources. Independent data extraction, risk‐of‐bias assessment, and analysis was performed by two investigators, enhancing the internal validity of the findings. The use of a frequentist network meta‐analysis model and the *p*‐score methodology for ranking interventions provides a ranking system that is more transparent and less prone to bias [27]. We produced comparison‐adjusted funnel plots to assess for publication bias or other small study effects, wherever there were sufficient trials in the analysis. We analysed efficacy in all patients randomised, but also according to previous exposure to advanced therapies, wherever possible. We also used a structured classification of histological outcomes, separated into histological‐endoscopic mucosal improvement, histological‐endoscopic mucosal remission and histological remission, to minimise heterogeneity by aligning studies with similar definitions and criteria.

However, some limitations of the network meta‐analysis should be acknowledged. Only 11 of 28 trials were at low risk of bias across all domains. While network meta‐analysis enables indirect comparisons, the absence of direct head‐to‐head trials for most therapies remains an important consideration. Additionally, long‐term safety data for some newer therapies, including IL‐23 inhibitors and JAK inhibitors, are still emerging and further research is needed to fully characterise their risk–benefit profiles [55]. There may also be differences in the speed at which cessation of each of these active therapies, which is inherent in the design of re‐randomised trials, leads to a loss of their beneficial effects. This may impact the results of the network for RCTs of this design. Potential publication bias was present in several analyses, meaning that the efficacy of some of these therapies may have been overestimated. With only two head‐to‐head trials of one drug versus another, both of which were treat‐through trials, there was limited ability to compare direct and indirect evidence. Finally, the variations in drug efficacy may stem from differences in endpoints used, trial design, including treatment duration, population characteristics, or drug administration protocols. More recent trials have used increasingly stringent endpoints for clinical remission, which includes the necessity for the rectal bleeding subscore of the Mayo score to be zero, and definitions of corticosteroid‐free remission differed, with some RCTs mandating a minimum duration that corticosteroids had to be discontinued for. As another example, the efficacy of vedolizumab varied between re‐randomised trials and treat‐through trials, possibly due to differences in administration methods (subcutaneous vs. intravenous), previous exposure to advanced therapies among recruited patients, or baseline disease severity. Similarly, mirikizumab exhibited strong effects in achieving histological‐endoscopic mucosal remission but did not demonstrate comparable efficacy in terms of clinical remission. Overall, upadacitinib's consistent performance across multiple efficacy endpoints in re‐randomised trials, and etrasimod's performance in treat‐through trials, both of which used the more stringent endpoints to assess efficacy, position them as attractive drugs, given they are administered orally. However, infliximab performed similarly to etrasimod in treat‐through trials, may be cheaper in some countries, and has a more well‐established safety profile. That said, treatment duration was 30 weeks or less in four of the six RCTs of infliximab [5, 33, 35, 36], which may have led to an overestimation of its efficacy.

These discrepancies underscore the need to interpret the results of network meta‐analyses in the context of study‐specific variables. A fundamental issue in this network meta‐analysis is whether drug efficacy can be inferred across different trial designs. For example, in terms of the highest ranked drugs, can the similar efficacy of etrasimod or infliximab, which were assessed solely in treat‐through trials, and upadacitinib or guselkumab, which were evaluated exclusively in re‐randomised trials, be assumed via the results from indirect comparisons with common comparators used in trials of both designs, such as vedolizumab 300 mg 8‐weekly or etrolizumab 105 mg 4‐weekly? This is problematic and, we believe, should be approached cautiously, particularly as the underlying trial methodologies differ substantially. This is evident from the difference in the magnitude of the treatment effects seen, which were larger in trials re‐randomising patients, where patients who had already responded to active drug as induction therapy were re‐randomised to active drug or placebo as maintenance therapy. These re‐randomised trials also mirror clinical practice more closely, compared with treat‐through trials. If a patient had not experienced a benefit after induction, it is unlikely the clinician would choose to continue the drug into the maintenance phase, and for up to 1 year.

Previous network meta‐analyses have evaluated the efficacy and safety of biological therapies and small molecules as maintenance therapy in UC. For instance, a recent Bayesian network meta‐analysis by Shebab et al. compared the relative efficacy of biologics and small molecules in achieving remission in patients with UC [14], demonstrating that upadacitinib as maintenance therapy ranked first in achieving endoscopic improvement and histological remission. Some trials we identified appear to have been excluded in this meta‐analysis, including trials of adalimumab [32], ozanimod [8, 39] and etrasimod [38]. In this study, ORs were used to evaluate treatment efficacy, instead of using RRs; ORs tend to overestimate effect sizes when the outcome is common, potentially leading to misleading conclusions, whereas RR offers a more stable and direct estimation of risk reduction [56]. In addition, in their main analysis, the authors pooled studies together irrespective of their design, only performing a sub‐analysis based on trial design. Another network meta‐analysis of maintenance trials [15], conducted for the American Association of Gastroenterology, concluded that upadacitinib and tofacitinib were associated with higher rates of remission in re‐randomised trials. In treat‐through trials, etrasimod was associated with a higher rate of remission, with infliximab ranked much lower than in our analysis, although the dose of the latter was not specified. However, many of the endpoints we report were not examined, and this network meta‐analysis has been criticised by some for its choice of statistical approach [57].

The growing availability of newer therapies highlights the need for continued discussions regarding their accessibility and reimbursement [58]. In clinical practice, considerations such as prior exposure to advanced therapies, baseline disease severity, safety profiles and patient preference, particularly with regard to route of administration, remain essential in selecting the most appropriate maintenance therapy. Given the variability in efficacy rankings between trials of re‐randomised and treat‐through design, clinicians may benefit from a more nuanced interpretation of study results, recognising how these differences in trial design may influence outcomes [59]. Despite the insights provided by this study, several questions remain. The long‐term safety and durability of response for IL‐23 inhibitors and JAK inhibitors warrant further investigation, particularly in real‐world settings and in long‐term extension trials [60]. Additionally, the observed differences in treatment effect and inability to compare the relative efficacy of drugs studied only in re‐randomised trials with those tested only in treat‐through trials highlight the need for further head‐to‐head RCTs to assess these deficits in current knowledge. Future research should also focus on identifying predictors of response to these different available therapeutic classes, optimising sequential treatment strategies and exploring biomarker‐driven approaches to the selection of first‐line therapy. Economic evaluations will also be important in assessing the relative cost‐effectiveness of different treatments in routine clinical practice [61]. As the therapeutic landscape for UC continues to evolve, these advances are essential to support clinicians in making well‐informed decisions for their patients.

In conclusion, this network meta‐analysis demonstrates that, in re‐randomised trials, upadacitinib 30 mg o.d. is the highest ranked drug for clinical remission and endoscopic improvement, vedolizumab 300 mg 4‐weekly for endoscopic remission and guselkumab 200 mg 4‐weekly for corticosteroid‐free remission. In treat‐through trials, etrasimod 2 mg o.d. ranked highest for clinical remission and corticosteroid‐free remission and infliximab 10 mg/kg 8‐weekly highest for endoscopic improvement. All drugs were, for the most part, safe and well‐tolerated. However, top‐ranked drugs varied according to previous exposure to advanced therapies and many drugs were of similar efficacy across all these endpoints. Direct inferences of relative efficacy between drugs studied only in re‐randomised trials with drugs studied only in treat‐through trials are inappropriate and head‐to‐head RCTs of the best performing drugs studied in different trial designs, comparing drugs such as upadacitinib, guselkumab, or vedolizumab with drugs such as etrasimod or infliximab, are warranted.

## Author Contributions

**Brigida Barberio:** conceptualization, investigation, methodology, writing – original draft, formal analysis. **David J. Gracie:** conceptualization, writing – original draft, writing – review and editing. **Christopher J. Black:** conceptualization, investigation, writing – original draft, methodology, data curation, formal analysis, writing – review and editing. **Alexander C. Ford:** conceptualization, methodology, investigation, writing – original draft, writing – review and editing, formal analysis, data curation, supervision.

## Disclosure

*Guarantor*: A.C.F. is the guarantor. He accepts full responsibility for the work and the conduct of the study, had access to the data and controlled the decision to publish. The corresponding author attests that all listed authors meet authorship criteria and that no others meeting the criteria have been omitted. *Patient and public involvement statement*: We did not involve patients or the public in this work. We will disseminate our findings in lay terms via the national charity for people living with digestive diseases, ‘Guts UK’.

## Conflicts of Interest

The authors declare no conflicts of interest.

## Supporting information


Data S1:

## Data Availability

The data that support the findings of this study are available from the corresponding author upon reasonable request.
