# Supplementary material for: Network Meta‐Analysis: Efficacy of Biological Therapies and Small Molecules as Maintenance Therapy in Ulcerative Colitis
Source: Aliment Pharmacol Ther. 2025 May 23;62(1):4–21. doi: 10.1111/apt.70209 (PMC12151545; doi:10.1111/apt.70209)
Supplement: Supplementary file 1 — Data S1: [file APT-62-4-s001.pdf]

SUPPLEMENTARY MATERIALS

SUPPLEMENTARY RESULTS

Safety in Trials Re-randomising Patients

All 16 trials reported adverse events data, containing 6305 patients.[1-16] For any treatment-emergent adverse event, heterogeneity was low between studies (τ^2 = 0.0095), with vedolizumab 108mg subcutaneously 2-weekly the least likely to lead to adverse events (RR of any treatment-emergent adverse event = 0.86; 95% CI 0.67 to 1.10, P-score 0.81) and ozanimod 1mg o.d. the most likely, and significantly more likely to lead to adverse events than placebo (RR = 1.34; 95% CI 1.01 to 1.79, P-score 0.07) (Supplementary Figure 34). After direct and indirect comparison, vedolizumab 108mg subcutaneously 2-weekly, ustekinumab 90mg 12-weekly, and etrolizumab 105mg 4-weekly were less likely to lead to treatment-emergent adverse events than golimumab 100mg 4-weekly, and vedolizumab 108mg subcutaneously 2-weekly, ustekinumab 90mg 12-weekly, etrolizumab 105mg 4-weekly, and risankizumab 360mg 8-weekly less likely to lead to treatment-emergent adverse events than ozanimod 1mg o.d.

In terms of serious adverse events, there was borderline moderate heterogeneity between studies (τ^2 = 0.14). Mirikizumab 200mg 4-weekly was the least likely to cause serious adverse events (RR of any serious adverse event = 0.43; 95% CI 0.15 to 1.20, P-score 0.84), and guselkumab 200mg 4-weekly the most likely, and significantly more likely to lead to adverse events than placebo (RR = 12.13; 95% CI 1.40 to 105.25, P-score 0.02) (Supplementary Figure 35). Following direct and indirect comparison, all drugs other than filgotinib 200mg or 100mg o.d., golimumab 100mg 4-weekly, etrolizumab 105mg 4-weekly, infliximab 120mg subcutaneously 2-weekly, and guselkumab 100mg 8-weekly were less likely to lead to serious adverse events than guselkumab 200mg 4-weekly.

For serious infections there was no heterogeneity between studies ($\tau^2 = 0$). No drug was more likely than placebo to lead to serious infection, but risankizumab 360mg 8-weekly ranked first (RR of any serious infection = 0.25; 95% CI 0.03 to 2.23; P-score 0.84) and guselkumab 200mg 4-weekly last (RR = 5.05; 95% CI 0.24 to 104.55, P-score 0.19) (Supplementary Figure 36). There were no significant differences between individual drugs after direct and indirect comparison.

There was no heterogeneity between trials for withdrawals due to adverse events ($\tau^2 = 0$). Mirikizumab 200mg 4-weekly ranked first (RR of withdrawal due to adverse events = 0.19; 95% CI 0.07 to 0.47, P-score 0.94) and filgotinib 100mg o.d. last (RR = 1.77; 95% CI 0.66 to 4.77, P-score 0.11) (Supplementary Figure 37). After direct and indirect comparison, withdrawals due to adverse events were less likely with mirikizumab 200mg 4-weekly than golimumab 100mg or 50mg 4-weekly, upadacitinib 30mg o.d., etrolizumab 105mg 4-weekly, filgotinib 200mg or 100mg o.d., infliximab 120mg subcutaneously 2-weekly, tofacitinib 10mg b.i.d., or risankizumab 360mg 8-weekly, Withdrawals due to adverse events were also less likely with ustekinumab 90mg 8-weekly than with etrolizumab 105mg 4-weekly, filgotinib 200mg or 100mg o.d., infliximab 120mg subcutaneously 2-weekly, tofacitinib 10mg o.d., or risankizumab 360mg 8-weekly. Finally, withdrawals due to adverse events were less likely with vedolizumab 300mg 4-weekly or 8-weekly, guselkumab 200mg 8-weekly, upadacitinib 15mg o.d., and ustekinumab 90mg 12-weekly than with filgotinib 100mg o.d.

Safety in Trials Treating Patients Through

All 12 trials reported adverse events data,[17-27] containing 3768 patients. For any treatment-emergent adverse event, there was no heterogeneity between studies ($\tau^2 = 0$), with vedolizumab 300mg 8-weekly the least likely to lead to adverse events (RR of any treatment-emergent adverse event = 0.91; 95% CI 0.81 to 1.03, P-score 0.97) and etrasimod 2mg o.d. the most likely, and significantly more likely to lead to adverse events than placebo (RR = 1.27; 95% CI 1.08 to 1.49, P-

score 0.04) (Supplementary Figure 38). After direct and indirect comparison, vedolizumab 300mg 8-weekly was less likely to lead to treatment-emergent adverse events than infliximab 5mg/kg or 10mg/kg 8-weekly or etrasimod 2mg o.d.

In terms of serious adverse events, again all 12 RCTs provided data,[17-27] containing 3768 patients. There was low heterogeneity between studies (τ^2 = 0.021). Etrolizumab 105mg 4-weekly was the least likely drug to cause serious adverse events (RR of any serious adverse event = 0.51; 95% CI 0.26 to 1.02, P-score 0.91), and adalimumab 40mg 2-weekly the most likely (RR = 1.10; 95% CI 0.72 to 1.66, P-score 0.25) (Supplementary Figure 39). Following direct and indirect comparison, there were no significant differences between individual drugs.

For serious infections, data were available for 11 RCTs,[17-26] containing 3638 patients. There was no heterogeneity between studies (τ^2 = 0). No drug was more likely than placebo to lead to serious infection, but etrolizumab 105mg 4-weekly ranked first (RR of any serious infection = 0.24; 95% CI 0.05 to 1.19; P-score 0.90) and infliximab 10mg/kg 8-weekly last (RR = 1.79; 95% CI 0.70 to 4.57, P-score 0.13) (Supplementary Figure 40). After direct and indirect comparison, etrolizumab 105mg 4-weekly was less likely to lead to serious infection than infliximab 5mg/kg or 10mg/kg 8-weekly, and etrasimod 2mg o.d. was less likely to lead to serious infection than infliximab 10mg/kg 8-weekly.

For withdrawals due to adverse events, data were available for all 12 trials,[17-27] containing 3768 patients. There was moderate heterogeneity between trials for withdrawals due to adverse events (τ^2 = 0.27). Again, no drug was more likely than placebo to lead to withdrawals due to adverse events but etrolizumab 105mg 4-weekly ranked first (RR of withdrawal due to adverse events = 0.61; 95% CI 0.16 to 2.37, P-score 0.65) and adalimumab 40mg 2-weekly last (RR = 1.07; 95% CI 0.45 to 2.54, P-score 0.33) (Supplementary Figure 41). There were no significant differences between individual drugs after direct and indirect comparison.

SUPPLEMENTARY TABLES

Supplementary Table 1. Eligibility Criteria.

Randomised controlled trials.
Ambulatory adult (aged ≥18 years) patients with ulcerative colitis (UC).
Compared biological therapies or small molecules* as maintenance therapies at the doses taken on into phase III randomised controlled trials with placebo or each other.
Minimum duration of follow-up of 26 weeks.
Assessment of clinical remission, endoscopic improvement, endoscopic remission, corticosteroid-free remission, or histological improvement or remission at last point of follow-up as treatment was completed.

*Anti-TNFα antibodies (infliximab, adalimumab, or golimumab), anti-integrin antibodies (vedolizumab or etrolizumab), anti-interleukin-12/23 antibodies (ustekinumab), anti-interleukin-23 antibodies (mirikizumab, risamkizumab, or guselkumab), janus kinase inhibitors (tofacitinib, filgotinib, or upadacitinib), or sphingosine-1-phosphate receptor modulators (ozanimod or etrasimod).

Supplementary Table 2. Total Number of Re-randomised Trials of Each Intervention, and Total Number of Included Patients with UC Assigned to Biological Therapies, Small Molecules, or Placebo.

	Intervention	Number of RCTs	Total Number of Patients	References
Active intervention	Infliximab 120mg SC 2-weekly	1	294	[1]
	Golimumab 100mg 4-weekly	2	186	[2,3]
	Golimumab 50mg 4-weekly	1	154	[2]
	Vedolizumab 300mg 8-weekly	3	217	[4-6]
	Vedolizumab 300mg 4-weekly	1	125	[4]
	Vedolizumab 108mg SC 2-weekly	1	106	[6]
	Etrolizumab 105mg 4-weekly	2	225	[7,8]
	Ustekinumab 90mg 8-weekly	1	176	[9]
	Ustekinumab 90mg 12-weekly	1	172	[9]
	Mirikizumab 200mg 4-weekly	1	365	[10]
	Risankizumab 360mg 8-weekly	1	186	[11]
	Risankizumab 180mg 8-weekly	1	179	[11]
	Guselkumab 200mg 4-weekly	1	190	[12]
	Guselkumab 100mg 8-weekly	1	188	[12]
	Tofacitinib 10mg b.i.d.	1	197	[13]

	Tofacitinib 5mg b.i.d.	1	198	[13]
	Filgotinib 200mg o.d.	1	202	[14]
	Filgotinib 100mg o.d.	1	179	[14]
	Upadacitinib 30mg o.d.	1	233	[15]
	Upadacitinib 15mg o.d.	1	225	[15]
	Ozanimod 1mg o.d.	1	230	[16]
Placebo		16	2341	[1-16]

Supplementary Table 3. Total Number of Treat-through Trials of Each Intervention, and Total Number of Included Patients with UC Assigned to Biological Therapies, Small Molecules, or Placebo.

	Intervention	Number of RCTs	Total Number of Patients	References
Active intervention	Infliximab 5mg 8-weekly	6	635	[17,18,21,22,25]
	Infliximab 10mg 8-weekly	2	242	[17]
	Adalimumab 40mg 2-weekly	3	821	[19,20,24]
	Vedolizumab 300mg 8-weekly	1	385	[24]
	Etrolizumab 105mg 4-weekly	1	199	[25]
	Ozanimod 1mg o.d.	2	132	[23,27]
	Etrasimod 2mg o.d.	1	289	[26]
Placebo		10	1068	[17-23,26,27]

Supplementary Table 4. Characteristics of Maintenance Trials of Biological Therapies or Small Molecules Re-randomising Patients with UC.

Study	Country and Number of Centres	Disease Distribution	Number of Patients (% Advanced Therapy-naïve) and Source	Active Therapy and Dosing Schedule (Number of Patients)	Placebo Dosing Schedule (Number of Patients)	Duration of Follow-up
**Hanauer 2024 LIBERTY-UC** [1]	Multinational, 92 centres	Not reported	438 (90) responders to infliximab induction therapy after 10 weeks	Infliximab 120mg subcutaneously 2-weekly (294)	Placebo 2-weekly (144)	44 weeks
**Sandborn 2014 PURSUIT-M** [2]	Multinational, 251 centres	100% extensive or left sided colitis	464 (100) responders to golimumab induction therapy after 6 weeks	Golimumab 100mg (154) or 50mg (154) 4-weekly	Placebo 4-weekly (156)	54 weeks
**Hibi 2017 PURSUIT-J** [3]	Japan, 49 centres	38% extensive colitis, 62% left sided	63 (100) responders to golimumab induction therapy after 6 weeks	Golimumab 100mg 4-weekly (32)	Placebo 4-weekly (31)	54 weeks
**Feagan 2013 GEMINI 1** [4]	Multinational, 211 centres	49% extensive colitis, 40% left sided	373 (60) responders to vedolizumab induction therapy after 6 weeks	Vedolizumab 300mg 4-weekly (125) or 8-weekly (122)	Placebo 4-weekly (126)	46 weeks

**Motoya 2019** [5]	Japan, 100 centres	61.5% extensive colitis, 38.5% left sided	83 (63) responders to vedolizumab induction therapy after 10 weeks	Vedolizumab 300mg 8-weekly (41)	Placebo 8-weekly (42)	50 weeks
**Sandborn 2020 VISIBLE 1** [6]	Multinational, 141 centres	44% extensive colitis, 42% left sided	216 (39) responders to vedolizumab induction therapy after 6 weeks	Vedolizumab 102mg subcutaneously 2-weekly (106) or 300mg 8-weekly (54)	Placebo 2-weekly and 8-weekly (56)	46 weeks
**Peyrin-Biroulet 2022 HICKORY** [7]	Multinational, 184 centres	49% extensive colitis, 51% left sided	232 (0) responders to etrolizumab induction therapy after 14 weeks	Etrolizumab 105mg 4-weekly (117)	Placebo 4-weekly (115)	52 weeks
**Vermeire 2022 LAUREL** [8]	Multinational, 111 centres	41% extensive colitis, 59% left sided	214 (100) responders to etrolizumab induction therapy after 10 weeks	Etrolizumab 105mg 4-weekly (108)	Placebo 4-weekly (106)	52 weeks
**Sands 2019 UNIFI** [9]	Multinational, 244 centres	47% extensive colitis, 53% left sided	523 (49) responders to ustekinumab induction therapy after 8 weeks	Ustekinumab 90mg 8-weekly (176) or 12-weekly (172)	Placebo (175)	44 weeks
**D'Haens 2023 LUCENT-2** [10]	Multinational, 367 centres	35% extensive colitis, 65% left sided	544 (63) responders to mirikizumab induction therapy after 12 weeks	Mirikizumab 200mg 4-weekly (365)	Placebo 4-weekly (179)	40 weeks

**Louis 2024 COMMAND** [11]	Multinational, 238 centres	52% extensive colitis, 48% left sided	548 (25) responders to risankizumab induction therapy after 12 or 24 weeks	Risankizumab 360mg (186) or 180mg (179) 8-weekly	Placebo 8-weekly (183)	52 weeks
**Rubin 2025 QUASAR** [12]	Multinational, 254 centres	45% extensive colitis, 55% left sided	568 (54) responders to guselkumab induction therapy after 12 weeks	Guselkumab 200mg 4-weekly (190) or 100mg 8-weekly (188)	Placebo 4-weekly (190)	44 weeks
**Sandborn 2017 OCTAVE Sustain** [13]	Multinational, 297 centres	53% extensive colitis, 33% left sided	593 (52) responders to tofacitinib induction therapy after 8 weeks	Tofacitinib 10mg (197) or 5mg (198) b.i.d.	Placebo b.i.d. (198)	52 weeks
**Feagan 2021 SELECTION** [14]	Multinational, 341 centres	Not reported	571 (57) responders to filgotinib induction therapy after 10 weeks	Filgotinib 200mg (202) or 100mg (179) o.d.	Placebo o.d. (190)	47 weeks
**Vermeire 2023 U-ACHIEVE** [15]	Multinational, 251 centres	Not reported	681 (51) responders to upadacitinib induction therapy after 8 weeks	Upadacitinib 30mg (233) or 15mg (225) o.d.	Placebo o.d. (223)	52 weeks
**Sandborn 2021 TRUE NORTH** [16]	Multinational, 285 centres	32% extensive colitis, 68% left sided	457 (not reported) responders to ozanimod induction therapy after 10 weeks	Ozanimod 1mg o.d. (230)	Placebo o.d. (227)	42 weeks

Supplementary Table 5. Characteristics of Maintenance Trials of Biological Therapies or Small Molecules Treating Patients with UC Through.

Study	Country and Number of Centres	Disease Distribution	Number of Patients (% Advanced Therapy-naïve) and Severity of UC	Active Therapy and Dosing Schedule (Number of Patients)	Placebo Dosing Schedule (Number of Patients)	Duration of Follow-up
**Rutgeerts 2005 ACT 1** [17]	Multinational, 62 centres	46% extensive colitis, 54% left sided	364 (100), Mayo score ≥6 with endoscopic subscore ≥2	Infliximab 5mg/kg (121) or 10mg/kg at 0, 2, and 6 weeks (122), then 8-weekly	Placebo at 0, 2, and 6 weeks, then 8-weekly (121)	54 weeks
**Rutgeerts 2005 ACT 2** [17]	Multinational, 55 centres	60% extensive colitis, 40% left sided	364 (100), Mayo score ≥6 with endoscopic subscore ≥2	Infliximab 5mg/kg (121) or 10mg/kg at 0, 2, and 6 weeks (120), then 8-weekly	Placebo at 0, 2, and 6 weeks, then 8-weekly (123)	30 weeks
**NCT01551290** [18]	China, 12 centres	Not reported	99 (100), Mayo score ≥6	Infliximab 5mg/kg at 0, 2, and 6 weeks, then 8-weekly (50)	Placebo at 0, 2, and 6 weeks, then 8-weekly (49)	26 weeks
**Jiang 2015** [21]	China, single centre	62% extensive colitis, 38% left sided	82 (100), Mayo score ≥6 with endoscopic subscore ≥2	Infliximab 5mg/kg at 0, 2, and 6 weeks, then 8-weekly (41)	Placebo at 0, 2, and 6 weeks, then 8-weekly (41)	30 weeks

**Kobayashi 2016** [22]	Japan, 67 centres	80% extensive colitis, 20% left sided	208 (100), Mayo score ≥6 with endoscopic subscore ≥2	Infliximab 5mg/kg at 0, 2, and 6 weeks, then 8-weekly (104)	Placebo at 0, 2, and 6 weeks, then 8-weekly (104)	30 weeks
**Danese 2022 GARDENIA** [25]	Multinational, 114 centres	37% extensive colitis, 63% left sided	397 (100), Mayo score ≥6 with endoscopic subscore ≥2	Infliximab 5mg/kg at 0, 2, and 6 weeks, then 8-weekly (198) or etrolizumab 105mg 4-weekly (199)	N/A	52 weeks
**Sandborn 2012 ULTRA 2** [19]	Multinational, 103 centres	49% extensive colitis, 51% left sided	518 (60), Mayo score ≥6 with endoscopic subscore ≥2	Adalimumab 160/80mg at weeks 0 and 2, then 40mg 2-weekly (258)	Placebo 2-weekly (260)	52 weeks
**Suzuki 2014** [20]	Japan, 65 centres	64% extensive colitis, 36% left sided	273 (100), Mayo score ≥6 with endoscopic subscore ≥2	Adalimumab 160/80mg or 80/40mg at weeks 0 and 2, then 40mg 2-weekly (177)	Placebo 2-weekly (96)	32 weeks
**Sands 2019 VARSITY** [24]	Multinational, 245 centres	Not reported	771 (79), Mayo score ≥6 with endoscopic subscore ≥2	Vedolizumab 300mg at weeks 0, 2, and 6, then 8-weekly (385) or adalimumab 160/80mg at weeks 0 and 2, then 40mg 2-weekly (386)	N/A	52 weeks
**Sandborn 2016 TOUCHSTONE** [23]	Multinational, 57 centres	38% extensive colitis, 62% left sided	132 (83), Mayo score ≥6 with endoscopic subscore ≥2	Ozanimod 1mg o.d. (67)	Placebo o.d. (65)	32 weeks

**Nakase 2024 J-TRUE NORTH** [27]	Japan, number of centres not reported	Not reported	130 (not reported), Mayo score ≥6 with endoscopic subscore ≥2	Ozanimod 1mg o.d. (65)	Placebo o.d. (65)	52 weeks
**Sandborn 2023 ELEVATE UC 52** [26]	Multinational, 315 centres	32% extensive colitis, 67% left sided	433 (62), modified Mayo score ≥4 with endoscopic subscore ≥2	Etrasimod 2mg o.d. (289)	Placebo o.d. (144)	52 weeks

Supplementary Table 6. Risk of Bias of Maintenance Trials of Biological Therapies or Small Molecules Re-randomising Patients with UC.

**Study**	**Method of Generation of Randomisation Schedule Stated?**	**Method of Concealment of Treatment Allocation Stated?**	**Blinding?**	**No Evidence of Incomplete Outcomes Data?**	**No Evidence of Selective Reporting of Outcomes?**
**Hanauer 2024 LIBERTY-UC** [1]	Low	Low	Low	Low	Low
**Sandborn 2014 PURSUIT-M** [2]	Unclear	Unclear	Low	High	Low
**Hibi 2017 PURSUIT-J** [3]	Low	Unclear	Low	Low	Low
**Feagan 2013 GEMINI 1** [4]	Low	Low	Low	Low	Low
**Motoya 2019** [5]	Unclear	Low	Low	Low	Low
**Sandborn 2020 VISIBLE 1** [6]	Unclear	Unclear	Low	Low	Low
**Peyrin-Biroulet 2022 HICKORY** [7]	Low	Low	Low	High	Low
**Vermeire 2022 LAUREL** [8]	Low	Low	Low	High	Low

**Sands 2019 UNIFI [9]**	Unclear	Unclear	Low	Low	Low
**D'Haens 2023 LUCENT-2 [10]**	Low	Low	Low	Low	Low
**Louis 2024 COMMAND [11]**	Low	Low	Low	Low	Low
**Rubin 2025 QUASAR [12]**	Low	Low	Low	Low	Low
**Sandborn 2017 OCTAVE Sustain [13]**	Unclear	Low	Low	Low	Low
**Feagan 2021 SELECTION [14]**	Unclear	Low	Low	High	Low
**Vermeire 2023 U-ACHIEVE [15]**	Low	Low	Low	Low	Low
**Sandborn 2021 TRUE NORTH [16]**	Unclear	Low	Low	Low	Low

Supplementary Table 7. Risk of Bias of Maintenance Trials of Biological Therapies or Small Molecules Treating Patients with UC Through.

Study	Method of Generation of Randomisation Schedule Stated?	Method of Concealment of Treatment Allocation Stated?	Blinding?	No Evidence of Incomplete Outcomes Data?	No Evidence of Selective Reporting of Outcomes?
**Rutgeerts 2005 ACT 1 [17]**	Unclear	Low	Low	Low	Low
**Rutgeerts 2005 ACT 2 [17]**	Unclear	Low	Low	Low	Low
**NCT01551290 [18]**	Unclear	Unclear	Low	Low	Low
**Jiang 2015 [21]**	Unclear	Low	Low	Low	Low
**Kobayashi 2016 [22]**	Low	Low	Low	Low	Low
**Danese 2022 [25] GARDENIA**	Low	Low	Low	Low	Low
**Sandborn 2012 ULTRA 2 [19]**	Unclear	Low	Low	High	Low
**Suzuki 2014 [20]**	Low	Low	Low	Low	Low
**Sands 2019 VARSITY [24]**	Low	Low	Low	High	Low
**Sandborn 2016 TOUCHSTONE [23]**	Low	Low	Low	Low	Low

**Nakase 2024 J-TRUE NORTH** [27]	Unclear	Unclear	Low	Low	Unclear
**Sandborn 2023 ELEVATE UC 52** [26]	Low	Low	Low	Low	Low

Supplementary Table 8. Endpoints Reported in Maintenance Trials of Biological Therapies or Small Molecules Re-randomising Patients with UC.

Study	Clinical Endpoints	Endoscopic Endpoints	Histological Endpoints	Safety Endpoints
**Hanauer 2024**<br>**LIBERTY-UC** [1]	Clinical remission (Mayo score of ≤2 points, with no individual subscore >1 and a rectal bleeding subscore of 0)<br>Corticosteroid-free remission (clinical remission without receiving any corticosteroids for ≥8 weeks at week 44 in patients receiving corticosteroids at baseline)	None	Histological-endoscopic mucosal improvement (Robarts score <4 and Mayo endoscopic score ≤1)	Treatment-emergent adverse events<br>Serious adverse events<br>Serious infections<br>Adverse events leading to withdrawal

**Sandborn 2014 PURSUIT-M** [2]	Clinical remission (Mayo score of ≤2 points, with no individual subscore >1)<br>Corticosteroid-free remission (clinical remission without receiving any corticosteroids at week 54 in patients receiving corticosteroids at baseline)	Endoscopic improvement<br>Endoscopic remission	None	Treatment-emergent adverse events<br>Serious adverse events<br>Serious infections<br>Adverse events leading to withdrawal
**Hibi 2017 PURSUIT-J** [3]	Clinical remission (Mayo score of ≤2 points, with no individual subscore >1)<br>Corticosteroid-free remission (clinical remission without receiving any corticosteroids at week 54 in patients receiving corticosteroids at baseline)	Endoscopic improvement (endoscopic subscore ≤1)	None	Treatment-emergent adverse events<br>Serious adverse events<br>Serious infections<br>Adverse events leading to withdrawal

**Feagan 2013 GEMINI 1** [4]	Clinical remission (Mayo score of ≤2 points, with no individual subscore >1)<br>Corticosteroid-free remission (clinical remission without receiving any corticosteroids at week 46 in patients receiving corticosteroids at baseline)	Endoscopic improvement (endoscopic subscore ≤1)<br>Endoscopic remission (endoscopic subscore of 0)	None	Treatment-emergent adverse events<br>Serious adverse events<br>Serious infections<br>Adverse events leading to withdrawal
**Motoya 2019** [5]	Clinical remission (Mayo score of ≤2 points, with no individual subscore >1)<br>Corticosteroid-free remission (clinical remission without receiving any corticosteroids at week 50 in patients receiving corticosteroids at baseline)	Endoscopic improvement (endoscopic subscore ≤1)	None	Treatment-emergent adverse events<br>Serious adverse events<br>Serious infections<br>Adverse events leading to withdrawal

**Sandborn 2020 VISIBLE 1** [6]	Clinical remission (Mayo score of ≤2 points, with no individual subscore >1)<br>Corticosteroid-free remission (clinical remission without receiving any corticosteroids at week 46 in patients receiving corticosteroids at baseline)	Endoscopic improvement (endoscopic subscore ≤1)<br>Endoscopic remission (endoscopic subscore of 0)	Histological remission (Geboes score <2)	Treatment-emergent adverse events<br>Serious adverse events<br>Serious infections<br>Adverse events leading to withdrawal
**Peyrin-Biroulet 2022 HICKORY** [7]	Clinical remission (Mayo score of ≤2 points, with no individual subscore >1 and a rectal bleeding subscore of 0)<br>Corticosteroid-free remission (clinical remission without receiving any corticosteroids for ≥24 weeks at week 52 in patients receiving corticosteroids at baseline)	Endoscopic improvement (endoscopic subscore ≤1)<br>Endoscopic remission (endoscopic subscore of 0)	Histological remission (Nancy index ≤1)	Treatment-emergent adverse events<br>Serious adverse events<br>Serious infections<br>Adverse events leading to withdrawal

**Vermeire 2022 LAUREL** [8]	Clinical remission (Mayo score of ≤2 points, with no individual subscore >1 and a rectal bleeding subscore of 0)<br>Corticosteroid-free remission (clinical remission without receiving any corticosteroids for ≥24 weeks at week 52 in patients receiving corticosteroids at baseline)	Endoscopic improvement (endoscopic subscore ≤1)<br>Endoscopic remission (endoscopic subscore of 0)	Histological remission (Nancy index ≤1)	Treatment-emergent adverse events<br>Serious adverse events<br>Serious infections<br>Adverse events leading to withdrawal
**Sands 2019 UNIFI** [9]	Clinical remission (Mayo score of ≤2 points, with no individual subscore >1)<br>Corticosteroid-free remission (clinical remission without receiving any corticosteroids for ≥90 days at week 44 in patients receiving corticosteroids at baseline)	Endoscopic improvement (endoscopic subscore ≤1)	Histological-endoscopic mucosal healing (neutrophil infiltration in <5% of crypts, no crypt destruction, and no erosions, ulcerations, or granulation tissue plus endoscopic improvement)	Treatment-emergent adverse events<br>Serious adverse events<br>Serious infections<br>Adverse events leading to withdrawal

**D'Haens 2023 LUCENT-2** [10]	Clinical remission (Mayo score of ≤2 points, with no individual subscore >1 and a rectal bleeding subscore of 0)<br>Corticosteroid-free remission (clinical remission without receiving any corticosteroids for ≥12 weeks at week 40 in patients receiving corticosteroids at baseline)	Endoscopic improvement (endoscopic subscore ≤1)	Histological-endoscopic mucosal remission (Geboes score <2 and Mayo endoscopic score = 0)	Treatment-emergent adverse events<br>Serious adverse events<br>Serious infections<br>Adverse events leading to withdrawal
**Louis 2024 COMMAND** [11]	Clinical remission (Mayo score of ≤2 points, with no individual subscore >1 and a rectal bleeding subscore of 0)	Endoscopic improvement (endoscopic subscore ≤1)<br>Endoscopic remission (endoscopic subscore of 0)	Histological-endoscopic mucosal remission (Geboes score <2 and Mayo endoscopic score = 0)<br>Histological remission (Geboes score <2)<br>Histological-endoscopic mucosal improvement (Geboes score <3.2 and Mayo endoscopic score ≤1)	Treatment-emergent adverse events<br>Serious adverse events<br>Serious infections<br>Adverse events leading to withdrawal

**Rubin 2025 QUASAR** [12]	Clinical remission (Mayo score of ≤2 points, with no individual subscore >1 and a rectal bleeding subscore of 0)<br>Corticosteroid-free remission (clinical remission without receiving any corticosteroids for ≥8 weeks at week 44 in patients receiving corticosteroids at baseline)	Endoscopic improvement (endoscopic subscore ≤1)<br>Endoscopic remission (endoscopic subscore of 0)	Histological remission (Geboes score <2)<br>Histological-endoscopic mucosal improvement (Geboes score <3.2 and Mayo endoscopic score ≤1)	Treatment-emergent adverse events<br>Serious adverse events<br>Serious infections<br>Adverse events leading to withdrawal
**Sandborn 2017 OCTAVE Sustain** [13]	Clinical remission (Mayo score of ≤2 points, with no individual subscore >1 and a rectal bleeding subscore of 0)<br>Corticosteroid-free remission (clinical remission without receiving any corticosteroids for ≥4 weeks at week 52 in patients receiving corticosteroids at baseline)	Endoscopic improvement (endoscopic subscore ≤1)<br>Endoscopic remission (endoscopic subscore of 0)	None	Treatment-emergent adverse events<br>Serious adverse events<br>Serious infections<br>Adverse events leading to withdrawal

**Feagan 2021 SELECTION** [14]	Clinical remission (Mayo score of ≤2 points, with no individual subscore >1 and a rectal bleeding subscore of 0)<br>Corticosteroid-free remission (clinical remission without receiving any corticosteroids for ≥6 months at week 47 in patients receiving corticosteroids at baseline)	Endoscopic improvement (endoscopic subscore ≤1)<br>Endoscopic remission (endoscopic subscore of 0)	Histological remission and endoscopic improvement (Geboes score <2 and Mayo endoscopic score ≤1)<br>Histological remission (Geboes score <2)	Treatment-emergent adverse events<br>Serious adverse events<br>Serious infections<br>Adverse events leading to withdrawal
**Vermeire 2023 U-ACHIEVE** [15]	Clinical remission (Mayo score of ≤2 points, with no individual subscore >1 and a rectal bleeding subscore of 0)<br>Corticosteroid-free remission (clinical remission without receiving any corticosteroids for ≥90 days at week 52 in patients receiving corticosteroids at baseline)	Endoscopic improvement (endoscopic subscore ≤1)<br>Endoscopic remission (endoscopic subscore of 0)	Histological-endoscopic mucosal remission (Geboes score <2 and Mayo endoscopic score = 0)<br>Histological-endoscopic mucosal improvement (Geboes score <3.2 and Mayo endoscopic score ≤1)	Treatment-emergent adverse events<br>Serious adverse events<br>Serious infections<br>Adverse events leading to withdrawal

**Sandborn 2021**<br>**TRUE NORTH** [16]	Clinical remission (Mayo score of ≤2 points, with no individual subscore >1 and a rectal bleeding subscore of 0)<br>Corticosteroid-free remission (clinical remission without receiving any corticosteroids for ≥12 weeks at week 42 in patients receiving corticosteroids at baseline)	Endoscopic improvement (endoscopic subscore ≤1)	Histological remission and endoscopic improvement (Geboes score <2 and Mayo endoscopic score ≤1)<br>Histological remission (Geboes score <2)	Treatment-emergent adverse events<br>Serious adverse events<br>Serious infections<br>Adverse events leading to withdrawal

Supplementary Table 9. Endpoints Reported in Maintenance Trials of Biological Therapies or Small Molecules Treating Patients with UC Through.

Study	Clinical Endpoints	Endoscopic Endpoints	Histological Endpoints	Safety Endpoints
**Rutgeerts 2005 ACT 1** [17]	Clinical remission (Mayo score of ≤2 points, with no individual subscore >1)<br>Corticosteroid-free remission (clinical remission without receiving any corticosteroids at week 54 in patients receiving corticosteroids at baseline)	Endoscopic improvement (endoscopic subscore ≤1)	None	Treatment-emergent adverse events<br>Serious adverse events<br>Serious infections<br>Adverse events leading to withdrawal

**Rutgeerts 2005 ACT 2** [17]	Clinical remission (Mayo score of ≤2 points, with no individual subscore >1)<br>Corticosteroid-free remission (clinical remission without receiving any corticosteroids at week 30 in patients receiving corticosteroids at baseline)	Endoscopic improvement (endoscopic subscore ≤1)	None	Treatment-emergent adverse events<br>Serious adverse events<br>Serious infections<br>Adverse events leading to withdrawal
**NCT01551290** [18]	Clinical remission (Mayo score of ≤2 points, with no individual subscore >1)<br>Corticosteroid-free remission (clinical remission without receiving any corticosteroids for ≥1 month at week 26 in patients receiving corticosteroids at baseline)	Endoscopic improvement (endoscopic subscore ≤1)	None	Treatment-emergent adverse events<br>Serious adverse events<br>Serious infections<br>Adverse events leading to withdrawal

**Jiang 2015** [21]	Clinical remission (Mayo score of ≤2 points, with no individual subscore >1)<br>Corticosteroid-free remission (clinical remission without receiving any corticosteroids at week 30 in patients receiving corticosteroids at baseline)	Endoscopic improvement (endoscopic subscore ≤1)	None	Treatment-emergent adverse events<br>Serious adverse events<br>Serious infections<br>Adverse events leading to withdrawal
**Kobayashi 2016** [22]	Clinical remission (Mayo score of ≤2 points, with no individual subscore >1)	Endoscopic improvement (endoscopic subscore ≤1)	None	Treatment-emergent adverse events<br>Serious adverse events<br>Serious infections<br>Adverse events leading to withdrawal

**Danese 2022** [25]<br>**GARDENIA**	Clinical remission (Mayo score of ≤2 points, with no individual subscore >1)<br>Corticosteroid-free remission (clinical remission without receiving any corticosteroids for ≥24 weeks at week 52 in patients receiving corticosteroids at baseline)	Endoscopic improvement (endoscopic subscore ≤1)<br>Endoscopic remission (endoscopic subscore of 0)	None	Treatment-emergent adverse events<br>Serious adverse events<br>Serious infections<br>Adverse events leading to withdrawal
**Sandborn 2012**<br>**ULTRA 2** [19]	Clinical remission (Mayo score of ≤2 points, with no individual subscore >1)<br>Corticosteroid-free remission (clinical remission without receiving any corticosteroids at week 52 in patients receiving corticosteroids at baseline)	Endoscopic improvement (endoscopic subscore ≤1)	None	Treatment-emergent adverse events<br>Serious adverse events<br>Serious infections<br>Adverse events leading to withdrawal

**Suzuki 2014** [20]	Clinical remission (Mayo score of ≤2 points, with no individual subscore >1)<br>Corticosteroid-free remission (clinical remission without receiving any corticosteroids at week 32 in patients receiving corticosteroids at baseline)	Endoscopic improvement (endoscopic subscore ≤1)	None	Treatment-emergent adverse events<br>Serious adverse events<br>Serious infections<br>Adverse events leading to withdrawal
**Sands 2019 VARSITY** [24]	Clinical remission (Mayo score of ≤2 points, with no individual subscore >1)<br>Corticosteroid-free remission (clinical remission without receiving any corticosteroids at week 32 in patients receiving corticosteroids at baseline)	Endoscopic improvement (endoscopic subscore ≤1)	Histological remission (Geboes score <2)	Treatment-emergent adverse events<br>Serious adverse events<br>Serious infections<br>Adverse events leading to withdrawal

**Sandborn 2016 TOUCHSTONE** [23]	Clinical remission (Mayo score of ≤2 points, with no individual subscore >1)	Endoscopic improvement (endoscopic subscore ≤1)<br>Endoscopic remission (endoscopic subscore of 0)	Histological remission (Geboes score <2)	Treatment-emergent adverse events<br>Serious adverse events<br>Serious infections<br>Adverse events leading to withdrawal
**Nakase 2024 J-TRUE NORTH** [27]	Clinical remission (Mayo score of ≤2 points, with no individual subscore >1)	None	None	Treatment-emergent adverse events<br>Serious adverse events<br>Adverse events leading to withdrawal

**Sandborn 2023**<br>**ELEVATE UC 52** [26]	Clinical remission (Mayo score of ≤2 points, with no individual subscore >1 and a rectal bleeding subscore of 0)<br>Corticosteroid-free remission (clinical remission without receiving any corticosteroids for ≥12 weeks at week 52 in patients receiving corticosteroids at baseline)	Endoscopic improvement (endoscopic subscore ≤1)	Histological remission and endoscopic improvement (Geboes score <2 and Mayo endoscopic score ≤1)	Treatment-emergent adverse events<br>Serious adverse events<br>Serious infections<br>Adverse events leading to withdrawal

Supplementary Table 10. Confidence in Network Meta-Analysis Framework Evaluating the Confidence in the Indirect and Direct Treatment Estimates From the Network for Failure to Achieve Clinical Remission in Trials Re-randomising Patients with UC.

Comparison	No. of studies	Within-study bias	Reporting bias	Indirectness	Imprecision	Heterogeneity	Incoherence	Confidence rating	Reason(s) for downgrading
**DIRECT EVIDENCE**									
Etrolizumab 105mg 4-weekly: Placebo	2	Major concerns	Low risk	No concerns	Major concerns	No concerns	No concerns	Low	Within-study bias; Imprecision
Filgotinib 100mg o.d.: Filgotinib 200mg o.d.	1	Major concerns	Low risk	No concerns	No concerns	Major concerns	No concerns	Low	Within-study bias; Heterogeneity
Filgotinib 100mg o.d.: Placebo	1	Major concerns	Low risk	No concerns	No concerns	Major concerns	No concerns	Low	Within-study bias; Heterogeneity
Filgotinib 200mg o.d.: Placebo	1	Major concerns	Low risk	No concerns	No concerns	No concerns	No concerns	Moderate	Within-study bias
Golimumab 100mg 4-weekly: Golimumab 50mg 4-weekly	1	Major concerns	Low risk	No concerns	Major concerns	No concerns	Some concerns	Low	Within-study bias; Imprecision
Golimumab 100mg 4-weekly: Placebo	2	Major concerns	Low risk	No concerns	No concerns	Major concerns	No concerns	Low	Within-study bias; Heterogeneity
Golimumab 50mg 4-weekly: Placebo	1	Major concerns	Low risk	No concerns	No concerns	Major concerns	Some concerns	Low	Within-study bias; Heterogeneity
Guselkumab 100mg 8-weekly: Guselkumab 200mg 4-weekly	1	No concerns	Low risk	No concerns	Major concerns	No concerns	No concerns	Moderate	Imprecision
Guselkumab 100mg 8-weekly: Placebo	1	No concerns	Low risk	No concerns	No concerns	No concerns	No concerns	High	NA
Guselkumab 200mg 4-weekly: Placebo	1	No concerns	Low risk	No concerns	No concerns	No concerns	No concerns	High	NA
Infliximab 120mg 2-weekly: Placebo	1	No concerns	Low risk	No concerns	No concerns	No concerns	No concerns	High	NA
Mirikizumab 200mg 4-weekly: Placebo	1	No concerns	Low risk	No concerns	No concerns	No concerns	No concerns	High	NA
Ozanimod 1mg o.d.: Placebo	1	No concerns	Low risk	No concerns	No concerns	No concerns	No concerns	High	NA
Placebo: Risankizumab 180mg 8-weekly	1	No concerns	Low risk	No concerns	No concerns	Major concerns	No concerns	Moderate	Heterogeneity
Placebo: Risankizumab 360mg 8-weekly	1	No concerns	Low risk	No concerns	No concerns	Major concerns	No concerns	Moderate	Heterogeneity
Placebo: Tofacitinib 10mg b.i.d.	1	No concerns	Low risk	No concerns	No concerns	No concerns	No concerns	High	NA
Placebo: Tofacitinib 5mg b.i.d.	1	No concerns	Low risk	No concerns	No concerns	No concerns	No concerns	High	NA
Placebo: Upadacitinib 15mg o.d.	1	No concerns	Low risk	No concerns	No concerns	No concerns	No concerns	High	NA

Placebo: Upadacitinib 30mg o.d.	1	No concerns	Low risk	No concerns	No concerns	No concerns	No concerns	High	NA
Placebo: Ustekinumab 90mg 12-weekly	1	Some concerns	Low risk	No concerns	No concerns	Major concerns	No concerns	Moderate	Heterogeneity
Placebo: Ustekinumab 90mg 8-weekly	1	Some concerns	Low risk	No concerns	No concerns	No concerns	No concerns	High	NA
Placebo: Vedolizumab 108mg S/C 2-weekly	1	Some concerns	Low risk	No concerns	No concerns	No concerns	No concerns	High	NA
Placebo: Vedolizumab 300mg 4-weekly	1	No concerns	Low risk	No concerns	No concerns	No concerns	No concerns	High	NA
Placebo: Vedolizumab 300mg 8-weekly	3	No concerns	Low risk	No concerns	No concerns	No concerns	No concerns	High	NA
Risankizumab 180mg 8-weekly: Risankizumab 360mg 8-weekly	1	No concerns	Low risk	No concerns	Major concerns	No concerns	No concerns	Moderate	Imprecision
Tofacitinib 10mg b.i.d.: Tofacitinib 5mg b.i.d.	1	No concerns	Low risk	No concerns	Major concerns	No concerns	No concerns	Moderate	Imprecision
Upadacitinib 15mg o.d.: Upadacitinib 30mg o.d.	1	No concerns	Low risk	No concerns	No concerns	Major concerns	No concerns	Moderate	Heterogeneity
Ustekinumab 90mg 12-weekly: Ustekinumab 90mg 8-weekly	1	Some concerns	Low risk	No concerns	Major concerns	No concerns	No concerns	Moderate	Imprecision
Vedolizumab 108mg S/C 2-weekly: Vedolizumab 300mg 8-weekly	1	Some concerns	Low risk	No concerns	Major concerns	No concerns	No concerns	Moderate	Imprecision
Vedolizumab 300mg 4-weekly: Vedolizumab 300mg 8-weekly	1	No concerns	Low risk	No concerns	Major concerns	No concerns	No concerns	Moderate	Imprecision
**INDIRECT EVIDENCE**									
Etrolizumab 105mg 4-weekly: Filgotinib 100mg o.d.	NA	Major concerns	Low risk	No concerns	Major concerns	No concerns	No concerns	Low	Within-study bias; Imprecision
Etrolizumab 105mg 4-weekly: Filgotinib 200mg o.d.	NA	Major concerns	Low risk	No concerns	No concerns	Some concerns	No concerns	Moderate	Within-study bias
Etrolizumab 105mg 4-weekly: Golimumab 100mg 4-weekly	NA	Major concerns	Low risk	No concerns	Some concerns	Some concerns	No concerns	Low	Within-study bias; Imprecision; Heterogeneity
Etrolizumab 105mg 4-weekly: Golimumab 50mg 4-weekly	NA	Major concerns	Low risk	No concerns	Major concerns	No concerns	No concerns	Low	Within-study bias; Imprecision
Etrolizumab 105mg 4-weekly: Guselkumab 100mg 8-weekly	NA	Some concerns	Low risk	No concerns	No concerns	Some concerns	No concerns	Moderate	Within-study bias; Heterogeneity
Etrolizumab 105mg 4-weekly: Guselkumab 200mg 4-weekly	NA	Some concerns	Low risk	No concerns	No concerns	No concerns	No concerns	High	NA
Etrolizumab 105mg 4-weekly: Infliximab 120mg 2-weekly	NA	Some concerns	Low risk	No concerns	No concerns	Some concerns	No concerns	Moderate	Within-study bias; Heterogeneity
Etrolizumab 105mg 4-weekly: Mirikizumab 200mg 4-weekly	NA	Some concerns	Low risk	No concerns	No concerns	No concerns	No concerns	High	NA
Etrolizumab 105mg 4-weekly: Ozanimod 1mg o.d.	NA	Some concerns	Low risk	No concerns	No concerns	Major concerns	No concerns	Moderate	Heterogeneity

Etrolizumab 105mg 4-weekly: Risankizumab 180mg 8-weekly	NA	Some concerns	Low risk	No concerns	Some concerns	Some concerns	No concerns	Moderate	Within-study bias; Imprecision
Etrolizumab 105mg 4-weekly: Risankizumab 360mg 8-weekly	NA	Some concerns	Low risk	No concerns	Major concerns	No concerns	No concerns	Moderate	Imprecision
Etrolizumab 105mg 4-weekly: Tofacitinib 10mg b.i.d.	NA	Some concerns	Low risk	No concerns	No concerns	No concerns	No concerns	High	NA
Etrolizumab 105mg 4-weekly: Tofacitinib 5mg b.i.d.	NA	Some concerns	Low risk	No concerns	No concerns	Major concerns	No concerns	Moderate	Heterogeneity
Etrolizumab 105mg 4-weekly: Upadacitinib 15mg o.d.	NA	Some concerns	Low risk	No concerns	No concerns	No concerns	No concerns	High	NA
Etrolizumab 105mg 4-weekly: Upadacitinib 30mg o.d.	NA	Some concerns	Low risk	No concerns	No concerns	No concerns	No concerns	High	NA
Etrolizumab 105mg 4-weekly: Ustekinumab 90mg 12-weekly	NA	Some concerns	Low risk	No concerns	Some concerns	Some concerns	No concerns	Moderate	Within-study bias; Imprecision
Etrolizumab 105mg 4-weekly: Ustekinumab 90mg 8-weekly	NA	Some concerns	Low risk	No concerns	No concerns	Major concerns	No concerns	Moderate	Heterogeneity
Etrolizumab 105mg 4-weekly: Vedolizumab 108mg S/C 2-weekly	NA	Some concerns	Low risk	No concerns	No concerns	Some concerns	No concerns	Moderate	Within-study bias; Heterogeneity
Etrolizumab 105mg 4-weekly: Vedolizumab 300mg 4-weekly	NA	Some concerns	Low risk	No concerns	No concerns	Some concerns	No concerns	Moderate	Within-study bias; Heterogeneity
Etrolizumab 105mg 4-weekly: Vedolizumab 300mg 8-weekly	NA	Some concerns	Low risk	No concerns	No concerns	No concerns	No concerns	High	NA
Filgotinib 100mg o.d.: Golimumab 100mg 4-weekly	NA	Major concerns	Low risk	No concerns	Major concerns	No concerns	No concerns	Low	Within-study bias; Imprecision
Filgotinib 100mg o.d.: Golimumab 50mg 4-weekly	NA	Major concerns	Low risk	No concerns	Major concerns	No concerns	No concerns	Low	Within-study bias; Imprecision
Filgotinib 100mg o.d.: Guselkumab 100mg 8-weekly	NA	Some concerns	Low risk	No concerns	No concerns	Major concerns	No concerns	Moderate	Heterogeneity
Filgotinib 100mg o.d.: Guselkumab 200mg 4-weekly	NA	Some concerns	Low risk	No concerns	No concerns	No concerns	No concerns	High	NA
Filgotinib 100mg o.d.: Infliximab 120mg 2-weekly	NA	Some concerns	Low risk	No concerns	No concerns	Major concerns	No concerns	Moderate	Heterogeneity
Filgotinib 100mg o.d.: Mirikizumab 200mg 4-weekly	NA	Some concerns	Low risk	No concerns	No concerns	Major concerns	No concerns	Moderate	Heterogeneity
Filgotinib 100mg o.d.: Ozanimod 1mg o.d.	NA	Some concerns	Low risk	No concerns	Major concerns	No concerns	No concerns	Moderate	Imprecision
Filgotinib 100mg o.d.: Risankizumab 180mg 8-weekly	NA	Some concerns	Low risk	No concerns	Major concerns	No concerns	No concerns	Moderate	Imprecision
Filgotinib 100mg o.d.: Risankizumab 360mg 8-weekly	NA	Some concerns	Low risk	No concerns	Major concerns	No concerns	No concerns	Moderate	Imprecision
Filgotinib 100mg o.d.: Tofacitinib 10mg b.i.d.	NA	Some concerns	Low risk	No concerns	No concerns	Some concerns	No concerns	Moderate	Within-study bias; Heterogeneity
Filgotinib 100mg o.d.: Tofacitinib 5mg b.i.d.	NA	Some concerns	Low risk	No concerns	Some concerns	Some concerns	No concerns	Moderate	Within-study bias; Imprecision
Filgotinib 100mg o.d.: Upadacitinib 15mg o.d.	NA	Some concerns	Low risk	No concerns	No concerns	Some concerns	No concerns	Moderate	Within-study bias; Heterogeneity

Filgotinib 100mg o.d.: Upadacitinib 30mg o.d.	NA	Some concerns	Low risk	No concerns	No concerns	No concerns	No concerns	High	NA
Filgotinib 100mg o.d.: Ustekinumab 90mg 12-weekly	NA	Some concerns	Low risk	No concerns	Major concerns	No concerns	No concerns	Moderate	Imprecision
Filgotinib 100mg o.d.: Ustekinumab 90mg 8-weekly	NA	Some concerns	Low risk	No concerns	Some concerns	Some concerns	No concerns	Moderate	Within-study bias; Imprecision
Filgotinib 100mg o.d.: Vedolizumab 108mg S/C 2-weekly	NA	Some concerns	Low risk	No concerns	No concerns	Major concerns	No concerns	Moderate	Heterogeneity
Filgotinib 100mg o.d.: Vedolizumab 300mg 4-weekly	NA	Some concerns	Low risk	No concerns	No concerns	Major concerns	No concerns	Moderate	Heterogeneity
Filgotinib 100mg o.d.: Vedolizumab 300mg 8-weekly	NA	Some concerns	Low risk	No concerns	No concerns	Major concerns	No concerns	Moderate	Heterogeneity
Filgotinib 200mg o.d.: Golimumab 100mg 4-weekly	NA	Major concerns	Low risk	No concerns	Major concerns	No concerns	No concerns	Low	Within-study bias; Imprecision
Filgotinib 200mg o.d.: Golimumab 50mg 4-weekly	NA	Major concerns	Low risk	No concerns	Some concerns	Some concerns	No concerns	Low	Within-study bias; Imprecision; Heterogeneity
Filgotinib 200mg o.d.: Guselkumab 100mg 8-weekly	NA	Some concerns	Low risk	No concerns	Major concerns	No concerns	No concerns	Moderate	Imprecision
Filgotinib 200mg o.d.: Guselkumab 200mg 4-weekly	NA	Some concerns	Low risk	No concerns	Major concerns	No concerns	No concerns	Moderate	Imprecision
Filgotinib 200mg o.d.: Infliximab 120mg 2-weekly	NA	Some concerns	Low risk	No concerns	Major concerns	No concerns	No concerns	Moderate	Imprecision
Filgotinib 200mg o.d.: Mirikizumab 200mg 4-weekly	NA	Some concerns	Low risk	No concerns	Major concerns	No concerns	No concerns	Moderate	Imprecision
Filgotinib 200mg o.d.: Ozanimod 1mg o.d.	NA	Some concerns	Low risk	No concerns	Major concerns	No concerns	No concerns	Moderate	Imprecision
Filgotinib 200mg o.d.: Risankizumab 180mg 8-weekly	NA	Some concerns	Low risk	No concerns	Major concerns	No concerns	No concerns	Moderate	Imprecision
Filgotinib 200mg o.d.: Risankizumab 360mg 8-weekly	NA	Some concerns	Low risk	No concerns	Major concerns	No concerns	No concerns	Moderate	Imprecision
Filgotinib 200mg o.d.: Tofacitinib 10mg b.i.d.	NA	Some concerns	Low risk	No concerns	Major concerns	No concerns	No concerns	Moderate	Imprecision
Filgotinib 200mg o.d.: Tofacitinib 5mg b.i.d.	NA	Some concerns	Low risk	No concerns	Major concerns	No concerns	No concerns	Moderate	Imprecision
Filgotinib 200mg o.d.: Upadacitinib 15mg o.d.	NA	Some concerns	Low risk	No concerns	Major concerns	No concerns	No concerns	Moderate	Imprecision
Filgotinib 200mg o.d.: Upadacitinib 30mg o.d.	NA	Some concerns	Low risk	No concerns	No concerns	Some concerns	No concerns	Moderate	Within-study bias; Heterogeneity
Filgotinib 200mg o.d.: Ustekinumab 90mg 12-weekly	NA	Major concerns	Low risk	No concerns	Major concerns	No concerns	No concerns	Low	Within-study bias; Imprecision
Filgotinib 200mg o.d.: Ustekinumab 90mg 8-weekly	NA	Major concerns	Low risk	No concerns	Major concerns	No concerns	No concerns	Low	Within-study bias; Imprecision
Filgotinib 200mg o.d.: Vedolizumab 108mg S/C 2-weekly	NA	Some concerns	Low risk	No concerns	Major concerns	No concerns	No concerns	Moderate	Imprecision

Filgotinib 200mg o.d.: Vedolizumab 300mg 4-weekly	NA	Some concerns	Low risk	No concerns	Major concerns	No concerns	No concerns	Moderate	Imprecision
Filgotinib 200mg o.d.: Vedolizumab 300mg 8-weekly	NA	Some concerns	Low risk	No concerns	Major concerns	No concerns	No concerns	Moderate	Imprecision
Golimumab 100mg 4-weekly: Guselkumab 100mg 8-weekly	NA	Some concerns	Low risk	No concerns	Some concerns	Some concerns	No concerns	Moderate	Within-study bias; Imprecision
Golimumab 100mg 4-weekly: Guselkumab 200mg 4-weekly	NA	Some concerns	Low risk	No concerns	No concerns	Major concerns	No concerns	Moderate	Heterogeneity
Golimumab 100mg 4-weekly: Infliximab 120mg 2-weekly	NA	Some concerns	Low risk	No concerns	Major concerns	No concerns	No concerns	Moderate	Imprecision
Golimumab 100mg 4-weekly: Mirikizumab 200mg 4-weekly	NA	Some concerns	Low risk	No concerns	Some concerns	Some concerns	No concerns	Moderate	Within-study bias; Imprecision
Golimumab 100mg 4-weekly: Ozanimod 1mg o.d.	NA	Some concerns	Low risk	No concerns	Major concerns	No concerns	No concerns	Moderate	Imprecision
Golimumab 100mg 4-weekly: Risankizumab 180mg 8-weekly	NA	Some concerns	Low risk	No concerns	Major concerns	No concerns	No concerns	Moderate	Imprecision
Golimumab 100mg 4-weekly: Risankizumab 360mg 8-weekly	NA	Some concerns	Low risk	No concerns	Major concerns	No concerns	No concerns	Moderate	Imprecision
Golimumab 100mg 4-weekly: Tofacitinib 10mg b.i.d.	NA	Some concerns	Low risk	No concerns	Some concerns	Some concerns	No concerns	Moderate	Within-study bias; Imprecision
Golimumab 100mg 4-weekly: Tofacitinib 5mg b.i.d.	NA	Some concerns	Low risk	No concerns	Major concerns	No concerns	No concerns	Moderate	Imprecision
Golimumab 100mg 4-weekly: Upadacitinib 15mg o.d.	NA	Some concerns	Low risk	No concerns	No concerns	Major concerns	No concerns	Moderate	Heterogeneity
Golimumab 100mg 4-weekly: Upadacitinib 30mg o.d.	NA	Some concerns	Low risk	No concerns	No concerns	No concerns	No concerns	High	NA
Golimumab 100mg 4-weekly: Ustekinumab 90mg 12-weekly	NA	Some concerns	Low risk	No concerns	Major concerns	No concerns	No concerns	Moderate	Imprecision
Golimumab 100mg 4-weekly: Ustekinumab 90mg 8-weekly	NA	Some concerns	Low risk	No concerns	Major concerns	No concerns	No concerns	Moderate	Imprecision
Golimumab 100mg 4-weekly: Vedolizumab 108mg S/C 2-weekly	NA	Some concerns	Low risk	No concerns	No concerns	Major concerns	No concerns	Moderate	Heterogeneity
Golimumab 100mg 4-weekly: Vedolizumab 300mg 4-weekly	NA	Some concerns	Low risk	No concerns	Some concerns	Some concerns	No concerns	Moderate	Within-study bias; Imprecision
Golimumab 100mg 4-weekly: Vedolizumab 300mg 8-weekly	NA	Some concerns	Low risk	No concerns	Some concerns	Some concerns	No concerns	Moderate	Within-study bias; Imprecision
Golimumab 50mg 4-weekly: Guselkumab 100mg 8-weekly	NA	Some concerns	Low risk	No concerns	No concerns	Major concerns	No concerns	Moderate	Heterogeneity
Golimumab 50mg 4-weekly: Guselkumab 200mg 4-weekly	NA	Some concerns	Low risk	No concerns	No concerns	Major concerns	No concerns	Moderate	Heterogeneity
Golimumab 50mg 4-weekly: Infliximab 120mg 2-weekly	NA	Some concerns	Low risk	No concerns	Some concerns	Some concerns	No concerns	Moderate	Within-study bias; Imprecision
Golimumab 50mg 4-weekly: Mirikizumab 200mg 4-weekly	NA	Some concerns	Low risk	No concerns	No concerns	Major concerns	No concerns	Moderate	Heterogeneity
Golimumab 50mg 4-weekly: Ozanimod 1mg o.d.	NA	Some concerns	Low risk	No concerns	Major concerns	No concerns	No concerns	Moderate	Imprecision

Golimumab 50mg 4-weekly: Risankizumab 180mg 8-weekly	NA	Some concerns	Low risk	No concerns	Major concerns	No concerns	No concerns	Moderate	Imprecision
Golimumab 50mg 4-weekly: Risankizumab 360mg 8-weekly	NA	Some concerns	Low risk	No concerns	Major concerns	No concerns	No concerns	Moderate	Imprecision
Golimumab 50mg 4-weekly: Tofacitinib 10mg b.i.d.	NA	Some concerns	Low risk	No concerns	No concerns	Major concerns	No concerns	Moderate	Heterogeneity
Golimumab 50mg 4-weekly: Tofacitinib 5mg b.i.d.	NA	Some concerns	Low risk	No concerns	Major concerns	No concerns	No concerns	Moderate	Imprecision
Golimumab 50mg 4-weekly: Upadacitinib 15mg o.d.	NA	Some concerns	Low risk	No concerns	No concerns	Major concerns	No concerns	Moderate	Heterogeneity
Golimumab 50mg 4-weekly: Upadacitinib 30mg o.d.	NA	Some concerns	Low risk	No concerns	No concerns	No concerns	No concerns	High	NA
Golimumab 50mg 4-weekly: Ustekinumab 90mg 12-weekly	NA	Some concerns	Low risk	No concerns	Major concerns	No concerns	No concerns	Moderate	Imprecision
Golimumab 50mg 4-weekly: Ustekinumab 90mg 8-weekly	NA	Some concerns	Low risk	No concerns	Major concerns	No concerns	No concerns	Moderate	Imprecision
Golimumab 50mg 4-weekly: Vedolizumab 108mg S/C 2-weekly	NA	Some concerns	Low risk	No concerns	No concerns	Major concerns	No concerns	Moderate	Heterogeneity
Golimumab 50mg 4-weekly: Vedolizumab 300mg 4-weekly	NA	Some concerns	Low risk	No concerns	No concerns	Major concerns	No concerns	Moderate	Heterogeneity
Golimumab 50mg 4-weekly: Vedolizumab 300mg 8-weekly	NA	Some concerns	Low risk	No concerns	No concerns	Major concerns	No concerns	Moderate	Heterogeneity
Guselkumab 100mg 8-weekly: Infliximab 120mg 2-weekly	NA	No concerns	Low risk	No concerns	Major concerns	No concerns	No concerns	Moderate	Imprecision
Guselkumab 100mg 8-weekly: Mirikizumab 200mg 4-weekly	NA	No concerns	Low risk	No concerns	Major concerns	No concerns	No concerns	Moderate	Imprecision
Guselkumab 100mg 8-weekly: Ozanimod 1mg o.d.	NA	No concerns	Low risk	No concerns	Major concerns	No concerns	No concerns	Moderate	Imprecision
Guselkumab 100mg 8-weekly: Risankizumab 180mg 8-weekly	NA	No concerns	Low risk	No concerns	Major concerns	No concerns	No concerns	Moderate	Imprecision
Guselkumab 100mg 8-weekly: Risankizumab 360mg 8-weekly	NA	No concerns	Low risk	No concerns	Some concerns	Some concerns	No concerns	Moderate	Imprecision; Heterogeneity
Guselkumab 100mg 8-weekly: Tofacitinib 10mg b.i.d.	NA	No concerns	Low risk	No concerns	Major concerns	No concerns	No concerns	Moderate	Imprecision
Guselkumab 100mg 8-weekly: Tofacitinib 5mg b.i.d.	NA	No concerns	Low risk	No concerns	Major concerns	No concerns	No concerns	Moderate	Imprecision
Guselkumab 100mg 8-weekly: Upadacitinib 15mg o.d.	NA	No concerns	Low risk	No concerns	Major concerns	No concerns	No concerns	Moderate	Imprecision
Guselkumab 100mg 8-weekly: Upadacitinib 30mg o.d.	NA	No concerns	Low risk	No concerns	No concerns	Major concerns	No concerns	Moderate	Heterogeneity
Guselkumab 100mg 8-weekly: Ustekinumab 90mg 12-weekly	NA	Some concerns	Low risk	No concerns	Some concerns	Some concerns	No concerns	Moderate	Within-study bias; Imprecision
Guselkumab 100mg 8-weekly: Ustekinumab 90mg 8-weekly	NA	Some concerns	Low risk	No concerns	Major concerns	No concerns	No concerns	Moderate	Imprecision

Guselkumab 100mg 8-weekly: Vedolizumab 108mg S/C 2-weekly	NA	No concerns	Low risk	No concerns	Major concerns	No concerns	No concerns	Moderate	Imprecision
Guselkumab 100mg 8-weekly: Vedolizumab 300mg 4-weekly	NA	No concerns	Low risk	No concerns	Major concerns	No concerns	No concerns	Moderate	Imprecision
Guselkumab 100mg 8-weekly: Vedolizumab 300mg 8-weekly	NA	No concerns	Low risk	No concerns	Major concerns	No concerns	No concerns	Moderate	Imprecision
Guselkumab 200mg 4-weekly: Infliximab 120mg 2-weekly	NA	No concerns	Low risk	No concerns	Major concerns	No concerns	No concerns	Moderate	Imprecision
Guselkumab 200mg 4-weekly: Mirikizumab 200mg 4-weekly	NA	No concerns	Low risk	No concerns	Major concerns	No concerns	No concerns	Moderate	Imprecision
Guselkumab 200mg 4-weekly: Ozanimod 1mg o.d.	NA	No concerns	Low risk	No concerns	No concerns	Major concerns	No concerns	Moderate	Heterogeneity
Guselkumab 200mg 4-weekly: Risankizumab 180mg 8-weekly	NA	No concerns	Low risk	No concerns	No concerns	Major concerns	No concerns	Moderate	Heterogeneity
Guselkumab 200mg 4-weekly: Risankizumab 360mg 8-weekly	NA	No concerns	Low risk	No concerns	No concerns	Major concerns	No concerns	Moderate	Heterogeneity
Guselkumab 200mg 4-weekly: Tofacitinib 10mg b.i.d.	NA	No concerns	Low risk	No concerns	Major concerns	No concerns	No concerns	Moderate	Imprecision
Guselkumab 200mg 4-weekly: Tofacitinib 5mg b.i.d.	NA	No concerns	Low risk	No concerns	Some concerns	Some concerns	No concerns	Moderate	Imprecision; Heterogeneity
Guselkumab 200mg 4-weekly: Upadacitinib 15mg o.d.	NA	No concerns	Low risk	No concerns	Major concerns	No concerns	No concerns	Moderate	Imprecision
Guselkumab 200mg 4-weekly: Upadacitinib 30mg o.d.	NA	No concerns	Low risk	No concerns	Major concerns	No concerns	No concerns	Moderate	Imprecision
Guselkumab 200mg 4-weekly: Ustekinumab 90mg 12-weekly	NA	No concerns	Low risk	No concerns	No concerns	Major concerns	No concerns	Moderate	Heterogeneity
Guselkumab 200mg 4-weekly: Ustekinumab 90mg 8-weekly	NA	Some concerns	Low risk	No concerns	Major concerns	No concerns	No concerns	Moderate	Imprecision
Guselkumab 200mg 4-weekly: Vedolizumab 108mg S/C 2-weekly	NA	No concerns	Low risk	No concerns	Major concerns	No concerns	No concerns	Moderate	Imprecision
Guselkumab 200mg 4-weekly: Vedolizumab 300mg 4-weekly	NA	No concerns	Low risk	No concerns	Major concerns	No concerns	No concerns	Moderate	Imprecision
Guselkumab 200mg 4-weekly: Vedolizumab 300mg 8-weekly	NA	No concerns	Low risk	No concerns	Major concerns	No concerns	No concerns	Moderate	Imprecision
Infliximab 120mg 2-weekly: Mirikizumab 200mg 4-weekly	NA	No concerns	Low risk	No concerns	Major concerns	No concerns	No concerns	Moderate	Imprecision
Infliximab 120mg 2-weekly: Ozanimod 1mg o.d.	NA	No concerns	Low risk	No concerns	Major concerns	No concerns	No concerns	Moderate	Imprecision
Infliximab 120mg 2-weekly: Risankizumab 180mg 8-weekly	NA	No concerns	Low risk	No concerns	Major concerns	No concerns	No concerns	Moderate	Imprecision
Infliximab 120mg 2-weekly: Risankizumab 360mg 8-weekly	NA	No concerns	Low risk	No concerns	Major concerns	No concerns	No concerns	Moderate	Imprecision
Infliximab 120mg 2-weekly: Tofacitinib 10mg b.i.d.	NA	No concerns	Low risk	No concerns	Major concerns	No concerns	No concerns	Moderate	Imprecision

Infliximab 120mg 2-weekly: Tofacitinib 5mg b.i.d.	NA	No concerns	Low risk	No concerns	Major concerns	No concerns	No concerns	Moderate	Imprecision
Infliximab 120mg 2-weekly: Upadacitinib 15mg o.d.	NA	No concerns	Low risk	No concerns	Major concerns	No concerns	No concerns	Moderate	Imprecision
Infliximab 120mg 2-weekly: Upadacitinib 30mg o.d.	NA	No concerns	Low risk	No concerns	No concerns	Some concerns	No concerns	High	NA
Infliximab 120mg 2-weekly: Ustekinumab 90mg 12-weekly	NA	Some concerns	Low risk	No concerns	Major concerns	No concerns	No concerns	Moderate	Imprecision
Infliximab 120mg 2-weekly: Ustekinumab 90mg 8-weekly	NA	Some concerns	Low risk	No concerns	Major concerns	No concerns	No concerns	Moderate	Imprecision
Infliximab 120mg 2-weekly: Vedolizumab 108mg S/C 2-weekly	NA	No concerns	Low risk	No concerns	Major concerns	No concerns	No concerns	Moderate	Imprecision
Infliximab 120mg 2-weekly: Vedolizumab 300mg 4-weekly	NA	No concerns	Low risk	No concerns	Major concerns	No concerns	No concerns	Moderate	Imprecision
Infliximab 120mg 2-weekly: Vedolizumab 300mg 8-weekly	NA	No concerns	Low risk	No concerns	Major concerns	No concerns	No concerns	Moderate	Imprecision
Mirikizumab 200mg 4-weekly: Ozanimod 1mg o.d.	NA	No concerns	Low risk	No concerns	Major concerns	No concerns	No concerns	Moderate	Imprecision
Mirikizumab 200mg 4-weekly: Risankizumab 180mg 8-weekly	NA	No concerns	Low risk	No concerns	Some concerns	Some concerns	No concerns	Moderate	Imprecision; Heterogeneity
Mirikizumab 200mg 4-weekly: Risankizumab 360mg 8-weekly	NA	No concerns	Low risk	No concerns	No concerns	Major concerns	No concerns	Moderate	Heterogeneity
Mirikizumab 200mg 4-weekly: Tofacitinib 10mg b.i.d.	NA	No concerns	Low risk	No concerns	Major concerns	No concerns	No concerns	Moderate	Imprecision
Mirikizumab 200mg 4-weekly: Tofacitinib 5mg b.i.d.	NA	No concerns	Low risk	No concerns	Major concerns	No concerns	No concerns	Moderate	Imprecision
Mirikizumab 200mg 4-weekly: Upadacitinib 15mg o.d.	NA	No concerns	Low risk	No concerns	Major concerns	No concerns	No concerns	Moderate	Imprecision
Mirikizumab 200mg 4-weekly: Upadacitinib 30mg o.d.	NA	No concerns	Low risk	No concerns	No concerns	Major concerns	No concerns	Moderate	Heterogeneity
Mirikizumab 200mg 4-weekly: Ustekinumab 90mg 12-weekly	NA	Some concerns	Low risk	No concerns	Some concerns	Some concerns	No concerns	Moderate	Within-study bias; Imprecision
Mirikizumab 200mg 4-weekly: Ustekinumab 90mg 8-weekly	NA	Some concerns	Low risk	No concerns	Major concerns	No concerns	No concerns	Moderate	Imprecision
Mirikizumab 200mg 4-weekly: Vedolizumab 108mg S/C 2-weekly	NA	No concerns	Low risk	No concerns	Major concerns	No concerns	No concerns	Moderate	Imprecision
Mirikizumab 200mg 4-weekly: Vedolizumab 300mg 4-weekly	NA	No concerns	Low risk	No concerns	Major concerns	No concerns	No concerns	Moderate	Imprecision
Mirikizumab 200mg 4-weekly: Vedolizumab 300mg 8-weekly	NA	No concerns	Low risk	No concerns	Major concerns	No concerns	No concerns	Moderate	Imprecision
Ozanimod 1mg o.d.: Risankizumab 180mg 8-weekly	NA	No concerns	Low risk	No concerns	Major concerns	No concerns	No concerns	Moderate	Imprecision
Ozanimod 1mg o.d.: Risankizumab 360mg 8-weekly	NA	No concerns	Low risk	No concerns	Major concerns	No concerns	No concerns	Moderate	Imprecision

Ozanimod 1mg o.d.: Tofacitinib 10mg b.i.d.	NA	No concerns	Low risk	No concerns	Some concerns	Some concerns	No concerns	Moderate	Imprecision; Heterogeneity
Ozanimod 1mg o.d.: Tofacitinib 5mg b.i.d.	NA	No concerns	Low risk	No concerns	Major concerns	No concerns	No concerns	Moderate	Imprecision
Ozanimod 1mg o.d.: Upadacitinib 15mg o.d.	NA	No concerns	Low risk	No concerns	Some concerns	Some concerns	No concerns	Moderate	Imprecision; Heterogeneity
Ozanimod 1mg o.d.: Upadacitinib 30mg o.d.	NA	No concerns	Low risk	No concerns	No concerns	No concerns	No concerns	High	NA
Ozanimod 1mg o.d.: Ustekinumab 90mg 12-weekly	NA	Some concerns	Low risk	No concerns	Major concerns	No concerns	No concerns	Moderate	Imprecision
Ozanimod 1mg o.d.: Ustekinumab 90mg 8-weekly	NA	Some concerns	Low risk	No concerns	Major concerns	No concerns	No concerns	Moderate	Imprecision
Ozanimod 1mg o.d.: Vedolizumab 108mg S/C 2-weekly	NA	No concerns	Low risk	No concerns	Some concerns	Some concerns	No concerns	Moderate	Imprecision; Heterogeneity
Ozanimod 1mg o.d.: Vedolizumab 300mg 4-weekly	NA	No concerns	Low risk	No concerns	Major concerns	No concerns	No concerns	Moderate	Imprecision
Ozanimod 1mg o.d.: Vedolizumab 300mg 8-weekly	NA	No concerns	Low risk	No concerns	Major concerns	No concerns	No concerns	Moderate	Imprecision
Risankizumab 180mg 8-weekly: Tofacitinib 10mg b.i.d.	NA	No concerns	Low risk	No concerns	Some concerns	Some concerns	No concerns	Moderate	Imprecision; Heterogeneity
Risankizumab 180mg 8-weekly: Tofacitinib 5mg b.i.d.	NA	No concerns	Low risk	No concerns	Major concerns	No concerns	No concerns	Moderate	Imprecision
Risankizumab 180mg 8-weekly: Upadacitinib 15mg o.d.	NA	No concerns	Low risk	No concerns	Some concerns	Some concerns	No concerns	Moderate	Imprecision; Heterogeneity
Risankizumab 180mg 8-weekly: Upadacitinib 30mg o.d.	NA	No concerns	Low risk	No concerns	No concerns	No concerns	No concerns	High	NA
Risankizumab 180mg 8-weekly: Ustekinumab 90mg 12-weekly	NA	No concerns	Low risk	No concerns	Major concerns	No concerns	No concerns	Moderate	Imprecision
Risankizumab 180mg 8-weekly: Ustekinumab 90mg 8-weekly	NA	No concerns	Low risk	No concerns	Major concerns	No concerns	No concerns	Moderate	Imprecision
Risankizumab 180mg 8-weekly: Vedolizumab 108mg S/C 2-weekly	NA	No concerns	Low risk	No concerns	Some concerns	Some concerns	No concerns	Moderate	Imprecision; Heterogeneity
Risankizumab 180mg 8-weekly: Vedolizumab 300mg 4-weekly	NA	No concerns	Low risk	No concerns	Some concerns	Some concerns	No concerns	Moderate	Imprecision; Heterogeneity
Risankizumab 180mg 8-weekly: Vedolizumab 300mg 8-weekly	NA	No concerns	Low risk	No concerns	Major concerns	No concerns	No concerns	Moderate	Imprecision
Risankizumab 360mg 8-weekly: Tofacitinib 10mg b.i.d.	NA	No concerns	Low risk	No concerns	No concerns	Major concerns	No concerns	Moderate	Heterogeneity
Risankizumab 360mg 8-weekly: Tofacitinib 5mg b.i.d.	NA	No concerns	Low risk	No concerns	Major concerns	No concerns	No concerns	Moderate	Imprecision
Risankizumab 360mg 8-weekly: Upadacitinib 15mg o.d.	NA	No concerns	Low risk	No concerns	No concerns	Major concerns	No concerns	Moderate	Heterogeneity
Risankizumab 360mg 8-weekly: Upadacitinib 30mg o.d.	NA	No concerns	Low risk	No concerns	No concerns	No concerns	No concerns	High	NA

Risankizumab 360mg 8-weekly: Ustekinumab 90mg 12-weekly	NA	No concerns	Low risk	No concerns	Major concerns	No concerns	No concerns	Moderate	Imprecision
Risankizumab 360mg 8-weekly: Ustekinumab 90mg 8-weekly	NA	Some concerns	Low risk	No concerns	Major concerns	No concerns	No concerns	Moderate	Imprecision
Risankizumab 360mg 8-weekly: Vedolizumab 108mg S/C 2-weekly	NA	No concerns	Low risk	No concerns	No concerns	Major concerns	No concerns	Moderate	Heterogeneity
Risankizumab 360mg 8-weekly: Vedolizumab 300mg 4-weekly	NA	No concerns	Low risk	No concerns	No concerns	Major concerns	No concerns	Moderate	Heterogeneity
Risankizumab 360mg 8-weekly: Vedolizumab 300mg 8-weekly	NA	No concerns	Low risk	No concerns	Some concerns	Some concerns	No concerns	Moderate	Imprecision; Heterogeneity
Tofacitinib 10mg b.i.d.: Upadacitinib 15mg o.d.	NA	No concerns	Low risk	No concerns	Major concerns	No concerns	No concerns	Moderate	Imprecision
Tofacitinib 10mg b.i.d.: Upadacitinib 30mg o.d.	NA	No concerns	Low risk	No concerns	No concerns	Major concerns	No concerns	Moderate	Heterogeneity
Tofacitinib 10mg b.i.d.: Ustekinumab 90mg 12-weekly	NA	No concerns	Low risk	No concerns	Some concerns	Some concerns	No concerns	Moderate	Imprecision; Heterogeneity
Tofacitinib 10mg b.i.d.: Ustekinumab 90mg 8-weekly	NA	Some concerns	Low risk	No concerns	Major concerns	No concerns	No concerns	Moderate	Imprecision
Tofacitinib 10mg b.i.d.: Vedolizumab 108mg S/C 2-weekly	NA	No concerns	Low risk	No concerns	Major concerns	No concerns	No concerns	Moderate	Imprecision
Tofacitinib 10mg b.i.d.: Vedolizumab 300mg 4-weekly	NA	No concerns	Low risk	No concerns	Major concerns	No concerns	No concerns	Moderate	Imprecision
Tofacitinib 10mg b.i.d.: Vedolizumab 300mg 8-weekly	NA	No concerns	Low risk	No concerns	Major concerns	No concerns	No concerns	Moderate	Imprecision
Tofacitinib 5mg b.i.d.: Upadacitinib 15mg o.d.	NA	No concerns	Low risk	No concerns	Major concerns	No concerns	No concerns	Moderate	Imprecision
Tofacitinib 5mg b.i.d.: Upadacitinib 30mg o.d.	NA	No concerns	Low risk	No concerns	No concerns	No concerns	No concerns	High	NA
Tofacitinib 5mg b.i.d.: Ustekinumab 90mg 12-weekly	NA	Some concerns	Low risk	No concerns	Major concerns	No concerns	No concerns	Moderate	Imprecision
Tofacitinib 5mg b.i.d.: Ustekinumab 90mg 8-weekly	NA	Some concerns	Low risk	No concerns	Major concerns	No concerns	No concerns	Moderate	Imprecision
Tofacitinib 5mg b.i.d.: Vedolizumab 108mg S/C 2-weekly	NA	No concerns	Low risk	No concerns	Major concerns	No concerns	No concerns	Moderate	Imprecision
Tofacitinib 5mg b.i.d.: Vedolizumab 300mg 4-weekly	NA	No concerns	Low risk	No concerns	Major concerns	No concerns	No concerns	Moderate	Imprecision
Tofacitinib 5mg b.i.d.: Vedolizumab 300mg 8-weekly	NA	No concerns	Low risk	No concerns	Major concerns	No concerns	No concerns	Moderate	Imprecision
Upadacitinib 15mg o.d.: Ustekinumab 90mg 12-weekly	NA	Some concerns	Low risk	No concerns	Some concerns	Some concerns	No concerns	Moderate	Within-study bias; Imprecision
Upadacitinib 15mg o.d.: Ustekinumab 90mg 8-weekly	NA	Some concerns	Low risk	No concerns	Major concerns	No concerns	No concerns	Moderate	Imprecision

Upadacitinib 15mg o.d.: Vedolizumab 108mg S/C 2-weekly	NA	No concerns	Low risk	No concerns	Major concerns	No concerns	No concerns	Moderate	Imprecision
Upadacitinib 15mg o.d.: Vedolizumab 300mg 4-weekly	NA	No concerns	Low risk	No concerns	Major concerns	No concerns	No concerns	Moderate	Imprecision
Upadacitinib 15mg o.d.: Vedolizumab 300mg 8-weekly	NA	No concerns	Low risk	No concerns	Major concerns	No concerns	No concerns	Moderate	Imprecision
Upadacitinib 30mg o.d.: Ustekinumab 90mg 12-weekly	NA	No concerns	Low risk	No concerns	No concerns	No concerns	No concerns	High	NA
Upadacitinib 30mg o.d.: Ustekinumab 90mg 8-weekly	NA	No concerns	Low risk	No concerns	No concerns	Some concerns	No concerns	High	NA
Upadacitinib 30mg o.d.: Vedolizumab 108mg S/C 2-weekly	NA	No concerns	Low risk	No concerns	Major concerns	No concerns	No concerns	Moderate	Imprecision
Upadacitinib 30mg o.d.: Vedolizumab 300mg 4-weekly	NA	No concerns	Low risk	No concerns	Some concerns	Some concerns	No concerns	Moderate	Imprecision; Heterogeneity
Upadacitinib 30mg o.d.: Vedolizumab 300mg 8-weekly	NA	No concerns	Low risk	No concerns	No concerns	Major concerns	No concerns	Moderate	Heterogeneity
Ustekinumab 90mg 12-weekly: Vedolizumab 108mg S/C 2-weekly	NA	Some concerns	Low risk	No concerns	Some concerns	Some concerns	No concerns	Moderate	Within-study bias; Imprecision
Ustekinumab 90mg 12-weekly: Vedolizumab 300mg 4-weekly	NA	Some concerns	Low risk	No concerns	Some concerns	Some concerns	No concerns	Moderate	Within-study bias; Imprecision
Ustekinumab 90mg 12-weekly: Vedolizumab 300mg 8-weekly	NA	Some concerns	Low risk	No concerns	Some concerns	Some concerns	No concerns	Moderate	Within-study bias; Imprecision
Ustekinumab 90mg 8-weekly: Vedolizumab 108mg S/C 2-weekly	NA	Some concerns	Low risk	No concerns	Major concerns	No concerns	No concerns	Moderate	Imprecision
Ustekinumab 90mg 8-weekly: Vedolizumab 300mg 4-weekly	NA	Some concerns	Low risk	No concerns	Major concerns	No concerns	No concerns	Moderate	Imprecision
Ustekinumab 90mg 8-weekly: Vedolizumab 300mg 8-weekly	NA	Some concerns	Low risk	No concerns	Major concerns	No concerns	No concerns	Moderate	Imprecision
Vedolizumab 108mg S/C 2-weekly: Vedolizumab 300mg 4-weekly	NA	No concerns	Low risk	No concerns	Major concerns	No concerns	No concerns	Moderate	Imprecision

This table shows the confidence rating of evidence for all direct and indirect comparisons across the network.

Within-study bias: This relates to the risk of bias assessment made for each included study. The studies' contributions are combined with the risk of bias judgments to evaluate within-study bias for each estimate from a network meta-analysis.

Reporting bias: This relates to the assessment of the risk of bias from missing data or incomplete reporting.

Indirectness: Each study included in the network is evaluated according to its relevance to the research question, classified into low, moderate, or high indirectness.

Imprecision: The evaluation of imprecision requires that the relative treatment effect representing a clinically important difference is defined. We set this at 0.05 which creates a range of equivalence between 0.95 and 1.05. The treatment effect of the 95% CI of each comparison is compared with the range of equivalence. A rating of "major concerns" is given if the 95% CI extends beyond the range of equivalence on the opposite side of the null effect line as the point estimate, i.e., compatible with clinically important treatment effects in both directions, and a rating of "some concerns" is given if the 95% CI extends into, but not beyond, the range of equivalence on the opposite side of the null effect line as the point estimate.

Heterogeneity: Network meta-analysis assumes a single heterogeneity variance across all comparisons, expressed as tau^2, and this can, in turn, be expressed as a prediction interval. The prediction interval shows where the true effect of a new study similar to the existing studies is expected to lie. The 95% CI of each comparison is compared to the prediction interval, with reference to the rang of equivalence. If both lead to the same conclusions, then there are "no concerns" regarding heterogeneity. A rating of "major concerns" is given if the prediction interval extends beyond the range of equivalence on the opposite side of the null effect line as the point estimate, i.e., compatible with clinically important treatment effects in both directions, and a rating of "some concerns" is given if the prediction interval extends into, but not beyond, the range of equivalence on the opposite side of the null effect line as the point estimate.

Incoherence: This evaluates the agreement between direct and indirect evidence for certain comparisons in the network (also referred to as inconsistency). Where the 95% CI of the direct and indirect treatment estimate for a comparison would lead to the same conclusion with reference to the range of equivalence, a rating of “no concerns” is given.

Overall confidence rating and process of downgrading confidence: The quality of evidence was downgraded by one level if there were “major concerns” in one area, or “some concerns” in two areas. Consequently, the overall confidence rating for each comparison was based on the additive effect of ratings across all assessment domains.

Supplementary Table 11. League Table for Failure to Achieve Clinical Remission in Trials Re-randomising Patients with UC Naïve to Advanced Therapies.

**UPA 30mg o.d.**							0.81<br>[0.53;<br>1.23]												0.55<br>[0.37;<br>0.82]
0.98<br>[0.54;<br>1.76]	**GUS 200mg 4-weekly**					0.84<br>[0.53;<br>1.33]													0.56<br>[0.36;<br>0.87]
0.99<br>[0.51;<br>1.93]	1.01<br>[0.51;<br>2.02]	**RIS 360mg 8-weekly**									0.78<br>[0.44;<br>1.40]								0.56<br>[0.32;<br>0.95]
0.96<br>[0.52;<br>1.77]	0.99<br>[0.53;<br>1.85]	0.97<br>[0.48;<br>1.97]	**VED 108mg S/C 2-weekly**																0.57<br>[0.36;<br>0.90]
0.89<br>[0.51;<br>1.57]	0.91<br>[0.51;<br>1.64]	0.90<br>[0.46;<br>1.76]	0.93<br>[0.50;<br>1.70]	**FIL 200mg o.d.**													0.70<br>[0.47;<br>1.05]		0.62<br>[0.41;<br>0.92]
0.86<br>[0.49;<br>1.52]	0.88<br>[0.49;<br>1.59]	0.87<br>[0.44;<br>1.71]	0.89<br>[0.48;<br>1.65]	0.97<br>[0.55;<br>1.71]	**VED 300mg 4-weekly**					0.90<br>[0.58;<br>1.42]									0.64<br>[0.42;<br>0.97]
0.82<br>[0.46;<br>1.46]	0.84<br>[0.53;<br>1.33]	0.83<br>[0.42;<br>1.64]	0.85<br>[0.46;<br>1.58]	0.92<br>[0.52;<br>1.64]	0.96<br>[0.54;<br>1.70]	**GUS 100mg 8-weekly**													0.67<br>[0.44;<br>1.01]
0.81<br>[0.53;<br>1.23]	0.83<br>[0.46;<br>1.48]	0.82<br>[0.42;<br>1.59]	0.84<br>[0.46;<br>1.53]	0.91<br>[0.52;<br>1.58]	0.94<br>[0.54;<br>1.65]	0.98<br>[0.56;<br>1.73]	**UPA 15mg o.d.**												0.68<br>[0.46;<br>1.00]
0.79<br>[0.45;<br>1.37]	0.80<br>[0.45;<br>1.44]	0.79<br>[0.41;<br>1.54]	0.82<br>[0.45;<br>1.49]	0.88<br>[0.50;<br>1.54]	0.91<br>[0.52;<br>1.60]	0.96<br>[0.54;<br>1.68]	0.97<br>[0.56;<br>1.68]	**MIR 200mg 4-weekly**											0.70<br>[0.47;<br>1.03]

0.78 [0.45; 1.35]	0.80 [0.45; 1.41]	0.79 [0.41; 1.51]	0.81 [0.45; 1.46]	0.87 [0.51; 1.51]	0.91 [0.52; 1.57]	0.95 [0.54; 1.65]	0.96 [0.56; 1.65]	0.99 [0.58; 1.70]	**IFX 120mg 2-weekly**										0.71 [0.49; 1.02]
0.78 [0.46; 1.32]	0.79 [0.46; 1.38]	0.79 [0.41; 1.49]	0.81 [0.45; 1.43]	0.87 [0.51; 1.48]	0.90 [0.59; 1.39]	0.94 [0.55; 1.61]	0.96 [0.57; 1.61]	0.99 [0.59; 1.66]	1.00 [0.60; 1.65]	**VED 300mg 8-weekly**									0.71 [0.50; 1.00]
0.77 [0.41; 1.47]	0.79 [0.41; 1.53]	0.78 [0.44; 1.40]	0.80 [0.41; 1.58]	0.87 [0.46; 1.64]	0.90 [0.47; 1.71]	0.94 [0.49; 1.79]	0.96 [0.51; 1.80]	0.99 [0.52; 1.85]	0.99 [0.53; 1.85]	1.00 [0.55; 1.83]	**RIS 180mg 8-weekly**								0.71 [0.43; 1.17]
0.76 [0.42; 1.37]	0.77 [0.42; 1.43]	0.76 [0.38; 1.53]	0.78 [0.42; 1.48]	0.85 [0.47; 1.53]	0.88 [0.48; 1.59]	0.92 [0.50; 1.67]	0.93 [0.52; 1.67]	0.96 [0.54; 1.72]	0.97 [0.55; 1.72]	0.97 [0.56; 1.70]	0.98 [0.50; 1.89]	**UST 90mg 8-weekly**			0.94 [0.60; 1.48]				0.73 [0.47; 1.13]
0.74 [0.45; 1.23]	0.76 [0.45; 1.29]	0.75 [0.40; 1.39]	0.77 [0.44; 1.34]	0.83 [0.50; 1.38]	0.86 [0.52; 1.43]	0.90 [0.54; 1.51]	0.92 [0.56; 1.50]	0.94 [0.57; 1.55]	0.95 [0.59; 1.54]	0.95 [0.60; 1.51]	0.96 [0.53; 1.72]	0.98 [0.58; 1.67]	**GOL 100mg 4-weekly**			0.99 [0.68; 1.45]			0.74 [0.55; 1.01]
0.72 [0.42; 1.26]	0.74 [0.42; 1.32]	0.73 [0.38; 1.41]	0.75 [0.42; 1.36]	0.81 [0.47; 1.41]	0.84 [0.48; 1.47]	0.88 [0.50; 1.54]	0.89 [0.52; 1.54]	0.92 [0.54; 1.59]	0.93 [0.55; 1.58]	0.93 [0.56; 1.55]	0.93 [0.50; 1.74]	0.96 [0.54; 1.71]	0.98 [0.60; 1.59]	**OZA 1mg o.d.**					0.76 [0.52; 1.11]
0.71 [0.40; 1.27]	0.73 [0.40; 1.33]	0.72 [0.36; 1.42]	0.74 [0.40; 1.37]	0.80 [0.45; 1.42]	0.82 [0.46; 1.48]	0.86 [0.48; 1.55]	0.88 [0.49; 1.55]	0.90 [0.51; 1.60]	0.91 [0.52; 1.59]	0.91 [0.53; 1.57]	0.91 [0.48; 1.75]	0.94 [0.60; 1.48]	0.96 [0.57; 1.61]	0.98 [0.56; 1.72]	**UST 90mg 12-weekly**				0.78 [0.51; 1.18]
0.68 [0.40; 1.17]	0.70 [0.40; 1.22]	0.69 [0.36; 1.31]	0.71 [0.40; 1.26]	0.76 [0.45; 1.31]	0.79 [0.46; 1.36]	0.83 [0.48; 1.43]	0.84 [0.50; 1.43]	0.87 [0.51; 1.47]	0.87 [0.52; 1.47]	0.88 [0.53; 1.44]	0.88 [0.48; 1.62]	0.90 [0.51; 1.59]	0.92 [0.64; 1.32]	0.94 [0.56; 1.58]	0.96 [0.55; 1.67]	**GOL 50mg 4-weekly**			0.86 [0.60; 1.25]
0.62 [0.36; 1.08]	0.64 [0.36; 1.13]	0.63 [0.33; 1.22]	0.65 [0.36; 1.17]	0.70 [0.47; 1.05]	0.73 [0.42; 1.26]	0.76 [0.44; 1.32]	0.77 [0.45; 1.32]	0.79 [0.46; 1.36]	0.80 [0.47; 1.36]	0.80 [0.48; 1.34]	0.81 [0.43; 1.50]	0.83 [0.47; 1.47]	0.84 [0.52; 1.37]	0.86 [0.51; 1.47]	0.88 [0.50; 1.55]	0.92 [0.55; 1.54]	**FIL 100mg o.d.**		0.88 [0.61; 1.28]
0.60 [0.35; 1.05]	0.62 [0.35; 1.10]	0.61 [0.32; 1.18]	0.63 [0.35; 1.14]	0.68 [0.39; 1.18]	0.70 [0.40; 1.23]	0.74 [0.42; 1.29]	0.75 [0.43; 1.29]	0.77 [0.45; 1.33]	0.78 [0.46; 1.32]	0.78 [0.47; 1.30]	0.78 [0.42; 1.46]	0.80 [0.45; 1.43]	0.82 [0.50; 1.33]	0.84 [0.49; 1.43]	0.85 [0.48; 1.51]	0.89 [0.53; 1.50]	0.97 [0.57; 1.65]	**ETRO 105mg 4-weekly**	0.91 [0.62; 1.33]

0.55 [0.37; 0.82]	0.56 [0.36; 0.87]	0.56 [0.32; 0.95]	0.57 [0.36; 0.90]	0.62 [0.41; 0.92]	0.64 [0.43; 0.96]	0.67 [0.44; 1.01]	0.68 [0.46; 1.00]	0.70 [0.47; 1.03]	0.71 [0.49; 1.02]	0.71 [0.50; 1.00]	0.71 [0.43; 1.17]	0.73 [0.47; 1.13]	0.74 [0.55; 1.01]	0.76 [0.52; 1.11]	0.78 [0.51; 1.18]	0.81 [0.56; 1.15]	0.88 [0.61; 1.28]	0.91 [0.62; 1.33]	**PLA**

Relative risk with 95% confidence intervals in parentheses. Comparisons, column versus row, should be read from left to right, and are ordered relative to their overall efficacy. The intervention in the top left position is ranked as best after the network meta-analysis of direct and indirect effects. Direct comparisons are provided above the drug labels, and indirect comparisons are below. Boxes shaded green denote a statistically significant difference.

ETRO: etrolizumab, FIL: filgotinib; GOL: golimumab; GUS: guselkumab; IFX: infliximab; MIR: mirikizumab; OZA: ozanimod; PLA: placebo; RIS: risankizumab; UPA: upadacitinib; UST: ustekinumab; VED: vedolizumab.

Supplementary Table 12. League Table for Failure to Achieve Clinical Remission in Trials Re-randomising Patients with UC Exposed to Advanced Therapies.

**UPA 30mg o.d.**					0.75 [0.58; 0.96]												0.49 [0.40; 0.61]
0.79 [0.60; 1.04]	**MIR 200mg 4-weekly**																0.63 [0.52; 0.76]
0.77 [0.56; 1.04]	0.97 [0.72; 1.31]	**VED 300mg 8-weekly**				0.97 [0.70; 1.33]											0.64 [0.51; 0.81]
0.76 [0.57; 1.01]	0.96 [0.73; 1.26]	0.99 [0.73; 1.33]	**GUS 100mg 8-weekly**	0.99 [0.77; 1.27]													0.65 [0.53; 0.79]
0.75 [0.57; 0.99]	0.96 [0.73; 1.24]	0.98 [0.73; 1.31]	0.99 [0.77; 1.27]	**GUS 200mg 4-weekly**													0.65 [0.55; 0.79]
0.75 [0.58; 0.96]	0.95 [0.74; 1.21]	0.97 [0.74; 1.28]	0.98 [0.77; 1.26]	0.99 [0.78; 1.26]	**UPA 15mg o.d.**												0.66 [0.56; 0.77]
0.72 [0.53; 0.99]	0.91 [0.67; 1.24]	0.94 [0.69; 1.29]	0.95 [0.70; 1.29]	0.96 [0.71; 1.29]	0.97 [0.72; 1.28]	**VED 300mg 4-weekly**											0.69 [0.54; 0.87]
0.70 [0.51; 0.97]	0.89 [0.65; 1.21]	0.91 [0.65; 1.28]	0.92 [0.67; 1.26]	0.93 [0.68; 1.26]	0.94 [0.70; 1.26]	0.97 [0.69; 1.37]	**VED 108mg S/C 2-weekly**										0.70 [0.55; 0.90]
0.68 [0.51; 0.90]	0.86 [0.66; 1.12]	0.88 [0.66; 1.19]	0.89 [0.68; 1.17]	0.90 [0.69; 1.17]	0.91 [0.71; 1.16]	0.94 [0.69; 1.28]	0.97 [0.71; 1.32]	**UST 90mg 8-weekly**							0.78 [0.64; 0.97]		0.73 [0.60; 0.88]

0.62 [0.48; 0.81]	0.79 [0.62; 1.02]	0.81 [0.62; 1.08]	0.82 [0.64; 1.06]	0.83 [0.65; 1.06]	0.83 [0.67; 1.05]	0.87 [0.65; 1.16]	0.89 [0.66; 1.20]	0.92 [0.72; 1.19]	**OZA 1mg o.d.**								0.79 [0.67; 0.93]
0.61 [0.41; 0.90]	0.77 [0.52; 1.13]	0.79 [0.53; 1.18]	0.80 [0.54; 1.18]	0.80 [0.55; 1.18]	0.81 [0.56; 1.17]	0.84 [0.56; 1.27]	0.86 [0.57; 1.31]	0.89 [0.61; 1.32]	0.97 [0.67; 1.41]	**IFX 120mg 2-weekly**							0.82 [0.58; 1.14]
0.61 [0.48; 0.78]	0.77 [0.61; 0.97]	0.79 [0.61; 1.03]	0.80 [0.63; 1.01]	0.81 [0.64; 1.01]	0.81 [0.66; 1.00]	0.84 [0.64; 1.10]	0.87 [0.66; 1.14]	0.90 [0.71; 1.13]	0.97 [0.79; 1.20]	1.00 [0.70; 1.44]	**FIL 200mg o.d.**		0.94 [0.80; 1.11]				0.81 [0.71; 0.92]
0.60 [0.46; 0.78]	0.76 [0.59; 0.97]	0.78 [0.59; 1.03]	0.79 [0.61; 1.01]	0.79 [0.62; 1.01]	0.80 [0.64; 1.00]	0.83 [0.62; 1.10]	0.85 [0.64; 1.14]	0.88 [0.69; 1.13]	0.96 [0.76; 1.20]	0.99 [0.68; 1.43]	0.98 [0.80; 1.21]	**RIS 180mg 8-weekly**		0.90 [0.76; 1.06]			0.83 [0.71; 0.97]
0.57 [0.45; 0.73]	0.73 [0.58; 0.92]	0.75 [0.58; 0.97]	0.75 [0.60; 0.95]	0.76 [0.61; 0.95]	0.77 [0.63; 0.94]	0.80 [0.61; 1.04]	0.82 [0.62; 1.08]	0.85 [0.67; 1.07]	0.92 [0.75; 1.13]	0.95 [0.66; 1.36]	0.94 [0.80; 1.11]	0.96 [0.78; 1.18]	**FIL 100mg o.d.**				0.86 [0.75; 0.98]
0.54 [0.42; 0.69]	0.68 [0.54; 0.86]	0.70 [0.54; 0.91]	0.71 [0.56; 0.90]	0.71 [0.57; 0.90]	0.72 [0.58; 0.89]	0.75 [0.56; 0.98]	0.77 [0.58; 1.02]	0.79 [0.63; 1.01]	0.86 [0.69; 1.07]	0.89 [0.62; 1.28]	0.88 [0.73; 1.07]	0.90 [0.76; 1.06]	0.94 [0.77; 1.14]	**RIS 360mg 8-weekly**			0.92 [0.80; 1.06]
0.53 [0.41; 0.69]	0.67 [0.53; 0.86]	0.69 [0.52; 0.91]	0.70 [0.54; 0.90]	0.70 [0.55; 0.90]	0.71 [0.57; 0.89]	0.74 [0.55; 0.98]	0.76 [0.56; 1.01]	0.78 [0.64; 0.97]	0.85 [0.68; 1.07]	0.88 [0.60; 1.27]	0.87 [0.71; 1.07]	0.89 [0.71; 1.11]	0.93 [0.75; 1.14]	0.99 [0.80; 1.22]	**UST 90mg 12-weekly**		0.93 [0.79; 1.09]
0.51 [0.40; 0.66]	0.65 [0.52; 0.82]	0.67 [0.51; 0.87]	0.68 [0.53; 0.86]	0.68 [0.54; 0.85]	0.69 [0.56; 0.84]	0.71 [0.54; 0.94]	0.73 [0.55; 0.97]	0.76 [0.60; 0.96]	0.82 [0.67; 1.02]	0.85 [0.59; 1.22]	0.84 [0.70; 1.02]	0.86 [0.70; 1.06]	0.89 [0.74; 1.08]	0.95 [0.79; 1.16]	0.97 [0.79; 1.19]	**ETRO 105mg 4-weekly**	0.96 [0.84; 1.10]
0.49 [0.40; 0.61]	0.63 [0.52; 0.76]	0.64 [0.51; 0.81]	0.65 [0.53; 0.79]	0.65 [0.55; 0.79]	0.66 [0.56; 0.77]	0.68 [0.54; 0.87]	0.70 [0.55; 0.90]	0.73 [0.60; 0.88]	0.79 [0.67; 0.93]	0.82 [0.58; 1.14]	0.81 [0.71; 0.92]	0.83 [0.71; 0.97]	0.86 [0.75; 0.98]	0.92 [0.80; 1.06]	0.93 [0.79; 1.09]	0.96 [0.84; 1.10]	**PLA**

Relative risk with 95% confidence intervals in parentheses. Comparisons, column versus row, should be read from left to right, and are ordered relative to their overall efficacy. The intervention in the top left position is ranked as best after the network meta-analysis of direct and indirect

effects. Direct comparisons are provided above the drug labels, and indirect comparisons are below. Boxes shaded green denote a statistically significant difference.

ETRO: etrolizumab, FIL: filgotinib; GUS: guselkumab; IFX: infliximab; MIR: mirikizumab; OZA: ozanimod; PLA: placebo; RIS: risankizumab; UPA: upadacitinib; UST: ustekinumab; VED: vedolizumab.

Supplementary Table 13. League Table for Failure to Achieve Endoscopic Improvement in Trials Re-randomising Patients with UC Naïve to Advanced Therapies.

**RIS 360mg 8-weekly**							0.59 [0.25; 1.39]								0.36 [0.16; 0.82]
0.87 [0.31; 2.48]	**UPA 30mg o.d.**					0.69 [0.35; 1.36]									0.42 [0.22; 0.80]
0.66 [0.23; 1.88]	0.76 [0.30; 1.92]	**GUS 200mg 4-weekly**						0.87 [0.44; 1.71]							0.55 [0.29; 1.05]
0.64 [0.23; 1.79]	0.73 [0.29; 1.82]	0.96 [0.39; 2.38]	**MIR 200mg 4-weekly**												0.57 [0.30; 1.07]
0.61 [0.22; 1.72]	0.70 [0.28; 1.75]	0.92 [0.37; 2.29]	0.96 [0.39; 2.36]	**FIL 200mg o.d.**										0.67 [0.35; 1.28]	0.59 [0.31; 1.12]
0.62 [0.19; 2.00]	0.72 [0.25; 2.07]	0.94 [0.33; 2.71]	0.98 [0.34; 2.79]	1.02 [0.36; 2.91]	**VED 300mg 8-weekly**										0.58 [0.25; 1.34]
0.60 [0.21; 1.69]	0.69 [0.35; 1.36]	0.91 [0.37; 2.25]	0.95 [0.39; 2.31]	0.98 [0.40; 2.41]	0.97 [0.34; 2.75]	**UPA 15mg o.d.**									0.60 [0.32; 1.14]
0.59 [0.25; 1.39]	0.67 [0.25; 1.79]	0.88 [0.33; 2.35]	0.92 [0.35; 2.41]	0.96 [0.36; 2.51]	0.94 [0.31; 2.84]	0.97 [0.37; 2.55]	**RIS 180mg 8-weekly**								0.62 [0.30; 1.28]
0.58 [0.20; 1.63]	0.66 [0.27; 1.65]	0.87 [0.44; 1.71]	0.91 [0.37; 2.22]	0.94 [0.38; 2.32]	0.93 [0.32; 2.65]	0.96 [0.39; 2.35]	0.99 [0.37; 2.59]	**GUS 100mg 8-weekly**							0.63 [0.33; 1.19]
0.56 [0.22; 1.44]	0.64 [0.29; 1.44]	0.85 [0.38; 1.89]	0.88 [0.40; 1.94]	0.92 [0.41; 2.02]	0.90 [0.34; 2.35]	0.93 [0.42; 2.05]	0.96 [0.40; 2.28]	0.97 [0.44; 2.15]	**GOL 100mg 4-weekly**			0.99 [0.53; 1.84]			0.65 [0.40; 1.04]
0.56 [0.19; 1.61]	0.64 [0.25; 1.64]	0.84 [0.33; 2.15]	0.88 [0.35; 2.20]	0.91 [0.36; 2.30]	0.90 [0.31; 2.61]	0.93 [0.37; 2.33]	0.96 [0.36; 2.56]	0.97 [0.38; 2.44]	1.00 [0.44; 2.27]	**UST 90mg 8-weekly**	0.92 [0.46; 1.83]				0.65 [0.33; 1.27]

0.52 [0.18; 1.47]	0.59 [0.24; 1.50]	0.78 [0.31; 1.96]	0.81 [0.33; 2.01]	0.84 [0.34; 2.10]	0.83 [0.29; 2.39]	0.86 [0.35; 2.13]	0.88 [0.33; 2.34]	0.89 [0.36; 2.23]	0.92 [0.41; 2.07]	0.92 [0.46; 1.83]	**UST 90mg 12-weekly**				0.70 [0.37; 1.35]
0.50 [0.18; 1.37]	0.57 [0.24; 1.38]	0.75 [0.31; 1.81]	0.79 [0.33; 1.86]	0.82 [0.34; 1.94]	0.80 [0.29; 2.22]	0.83 [0.35; 1.97]	0.85 [0.34; 2.17]	0.87 [0.36; 2.06]	0.89 [0.49; 1.61]	0.89 [0.37; 2.17]	0.97 [0.40; 2.33]	**GOL 50mg 4-weekly**			0.80 [0.43; 1.48]
0.46 [0.16; 1.28]	0.53 [0.21; 1.30]	0.69 [0.28; 1.70]	0.72 [0.30; 1.75]	0.75 [0.31; 1.82]	0.74 [0.26; 2.08]	0.76 [0.31; 1.85]	0.78 [0.30; 2.04]	0.80 [0.33; 1.94]	0.82 [0.37; 1.79]	0.82 [0.33; 2.04]	0.89 [0.36; 2.19]	0.92 [0.39; 2.15]	**ETRO 105mg 4-weekly**		0.79 [0.43; 1.47]
0.41 [0.15; 1.14]	0.47 [0.19; 1.16]	0.62 [0.25; 1.52]	0.65 [0.27; 1.56]	0.67 [0.35; 1.28]	0.66 [0.23; 1.86]	0.68 [0.28; 1.65]	0.70 [0.27; 1.82]	0.71 [0.29; 1.73]	0.73 [0.34; 1.60]	0.74 [0.30; 1.82]	0.80 [0.32; 1.96]	0.82 [0.35; 1.92]	0.90 [0.37; 2.15]	**FIL 100mg o.d.**	0.88 [0.48; 1.63]
0.36 [0.16; 0.82]	0.42 [0.22; 0.80]	0.55 [0.29; 1.05]	0.57 [0.30; 1.07]	0.59 [0.31; 1.12]	0.58 [0.25; 1.34]	0.60 [0.32; 1.14]	0.62 [0.30; 1.28]	0.63 [0.33; 1.19]	0.65 [0.40; 1.04]	0.65 [0.33; 1.27]	0.70 [0.37; 1.35]	0.73 [0.40; 1.30]	0.79 [0.43; 1.47]	0.88 [0.48; 1.63]	**PLA**

Relative risk with 95% confidence intervals in parentheses. Comparisons, column versus row, should be read from left to right, and are ordered relative to their overall efficacy. The intervention in the top left position is ranked as best after the network meta-analysis of direct and indirect effects. Direct comparisons are provided above the drug labels, and indirect comparisons are below. Boxes shaded green denote a statistically significant difference.

ETRO: etrolizumab, FIL: filgotinib; GOL: golimumab; GUS: guselkumab; MIR: mirikizumab; PLA: placebo; RIS: risankizumab; UPA: upadacitinib; UST: ustekinumab; VED: vedolizumab.

Supplementary Table 14. League Table for Failure to Achieve Endoscopic Improvement in Trials Re-randomising Patients with UC Exposed to Advanced Therapies.

**UPA 30mg o.d.**			0.72 [0.55; 0.96]										0.44 [0.35; 0.55]
0.89 [0.42; 1.90]	**VED 300mg 8-weekly**												0.49 [0.24; 1.02]
0.74 [0.54; 1.02]	0.83 [0.39; 1.77]	**GUS 100mg 8-weekly**			0.94 [0.72; 1.23]								0.59 [0.48; 0.73]
0.72 [0.55; 0.96]	0.81 [0.39; 1.71]	0.98 [0.74; 1.29]	**UPA 15mg o.d.**										0.61 [0.51; 0.72]
0.71 [0.52; 0.98]	0.80 [0.38; 1.70]	0.96 [0.71; 1.30]	0.98 [0.74; 1.30]	**MIR 200mg 4-weekly**									0.62 [0.50; 0.77]
0.70 [0.52; 0.94]	0.78 [0.37; 1.66]	0.94 [0.72; 1.23]	0.96 [0.74; 1.25]	0.98 [0.74; 1.31]	**GUS 200mg 4-weekly**								0.63 [0.52; 0.76]
0.62 [0.45; 0.85]	0.69 [0.33; 1.48]	0.83 [0.61; 1.13]	0.85 [0.65; 1.13]	0.87 [0.64; 1.18]	0.89 [0.66; 1.18]	**UST 90mg 8-weekly**						0.74 [0.59; 0.93]	0.71 [0.57; 0.88]
0.59 [0.43; 0.79]	0.66 [0.31; 1.39]	0.79 [0.59; 1.06]	0.81 [0.62; 1.05]	0.82 [0.61; 1.10]	0.84 [0.64; 1.10]	0.95 [0.71; 1.27]	**RIS 180mg 8-weekly**			0.85 [0.69; 1.05]			0.75 [0.62; 0.91]
0.53 [0.40; 0.70]	0.59 [0.28; 1.25]	0.71 [0.55; 0.93]	0.73 [0.58; 0.92]	0.74 [0.57; 0.97]	0.76 [0.59; 0.97]	0.85 [0.65; 1.12]	0.90 [0.70; 1.16]	**ETRO 105mg 4-weekly**					0.83 [0.71; 0.98]
0.53 [0.40; 0.69]	0.59 [0.28; 1.24]	0.71 [0.55; 0.92]	0.73 [0.58; 0.91]	0.74 [0.57; 0.96]	0.76 [0.59; 0.96]	0.85 [0.66; 1.11]	0.90 [0.71; 1.15]	1.00 [0.80; 1.24]	**FIL 200mg o.d.**		0.93 [0.79; 1.11]		0.83 [0.72; 0.96]
0.50 [0.37; 0.67]	0.56 [0.27; 1.18]	0.67 [0.51; 0.89]	0.69 [0.54; 0.88]	0.70 [0.53; 0.93]	0.72 [0.55; 0.93]	0.81 [0.61; 1.07]	0.85 [0.69; 1.05]	0.95 [0.75; 1.20]	0.95 [0.76; 1.19]	**RIS 360mg 8-weekly**			0.88 [0.74; 1.04]

0.49 [0.37; 0.65]	0.55 [0.26; 1.16]	0.66 [0.51; 0.86]	0.68 [0.54; 0.85]	0.69 [0.53; 0.90]	0.71 [0.56; 0.90]	0.80 [0.61; 1.04]	0.84 [0.66; 1.07]	0.93 [0.75; 1.16]	0.93 [0.79; 1.11]	0.98 [0.79; 1.23]	**FIL 100mg o.d.**		0.89 [0.77; 1.03]
0.46 [0.34; 0.61]	0.51 [0.24; 1.08]	0.62 [0.47; 0.82]	0.63 [0.49; 0.81]	0.64 [0.49; 0.85]	0.66 [0.50; 0.85]	0.74 [0.59; 0.93]	0.78 [0.60; 1.02]	0.87 [0.68; 1.10]	0.87 [0.69; 1.09]	0.91 [0.71; 1.17]	0.93 [0.74; 1.17]	**UST 90mg 12-weekly**	0.96 [0.80; 1.15]
0.44 [0.35; 0.55]	0.49 [0.24; 1.02]	0.59 [0.48; 0.73]	0.61 [0.51; 0.72]	0.62 [0.50; 0.77]	0.63 [0.52; 0.76]	0.71 [0.57; 0.88]	0.75 [0.62; 0.91]	0.83 [0.71; 0.98]	0.83 [0.72; 0.96]	0.88 [0.74; 1.04]	0.89 [0.77; 1.03]	0.96 [0.80; 1.15]	**PLA**

Relative risk with 95% confidence intervals in parentheses. Comparisons, column versus row, should be read from left to right, and are ordered relative to their overall efficacy. The intervention in the top left position is ranked as best after the network meta-analysis of direct and indirect effects. Direct comparisons are provided above the drug labels, and indirect comparisons are below. Boxes shaded green denote a statistically significant difference.

ETRO: etrolizumab, FIL: filgotinib; GUS: guselkumab; MIR: mirikizumab; PLA: placebo; RIS: risankizumab; UPA: upadacitinib; UST: ustekinumab; VED: vedolizumab.

Supplementary Table 15. League Table for Failure to Achieve Endoscopic Remission in Trials Re-randomising Patients with UC Naïve to Advanced Therapies.

**RIS 360mg 8-weekly**					0.76 [0.53; 1.09]				0.61 [0.44; 0.85]
0.84 [0.57; 1.22]	**GUS 200mg 4-weekly**		0.94 [0.75; 1.18]						0.73 [0.60; 0.89]
0.81 [0.57; 1.15]	0.96 [0.76; 1.22]	**UPA 30mg o.d.**		0.96 [0.82; 1.14]					0.76 [0.66; 0.87]
0.79 [0.54; 1.14]	0.94 [0.75; 1.18]	0.98 [0.78; 1.22]	**GUS 100mg 8-weekly**						0.78 [0.65; 0.93]
0.78 [0.55; 1.10]	0.93 [0.73; 1.17]	0.96 [0.82; 1.14]	0.98 [0.79; 1.23]	**UPA 15mg o.d.**					0.79 [0.69; 0.90]
0.76 [0.53; 1.09]	0.91 [0.66; 1.26]	0.94 [0.70; 1.26]	0.97 [0.70; 1.32]	0.98 [0.73; 1.31]	**RIS 180mg 8-weekly**				0.81 [0.62; 1.05]
0.74 [0.52; 1.06]	0.89 [0.69; 1.13]	0.92 [0.75; 1.12]	0.94 [0.74; 1.19]	0.95 [0.78; 1.16]	0.97 [0.72; 1.32]	**ETRO 105mg 4-weekly**			0.83 [0.71; 0.96]
0.71 [0.51; 1.01]	0.86 [0.68; 1.07]	0.89 [0.75; 1.05]	0.91 [0.74; 1.12]	0.92 [0.78; 1.09]	0.94 [0.71; 1.25]	0.97 [0.80; 1.16]	**GOL 50mg 4-weekly**	0.95 [0.84; 1.08]	0.86 [0.77; 0.96]
0.68 [0.48; 0.96]	0.81 [0.65; 1.01]	0.84 [0.71; 1.00]	0.86 [0.70; 1.06]	0.88 [0.74; 1.03]	0.89 [0.67; 1.18]	0.92 [0.77; 1.10]	0.95 [0.84; 1.08]	**GOL 100mg 4-weekly**	0.90 [0.81; 1.00]
0.61 [0.44; 0.85]	0.73 [0.60; 0.89]	0.76 [0.66; 0.87]	0.78 [0.65; 0.93]	0.79 [0.69; 0.90]	0.81 [0.62; 1.05]	0.83 [0.71; 0.96]	0.86 [0.77; 0.96]	0.90 [0.81; 1.00]	**PLA**

Relative risk with 95% confidence intervals in parentheses. Comparisons, column versus row, should be read from left to right, and are ordered relative to their overall efficacy. The intervention in the top left position is ranked as best after the network meta-analysis of direct and indirect effects. Direct comparisons are provided above the drug labels, and indirect comparisons are below. Boxes shaded green denote a statistically significant difference.

ETRO: etrolizumab, GOL: golimumab; GUS: guselkumab; PLA: placebo; RIS: risankizumab; UPA: upadacitinib.

Supplementary Table 16. League Table for Failure to Achieve Endoscopic Remission in Trials Re-randomising Patients with UC Exposed to Advanced Therapies.

**GUS 100mg 8-weekly**			0.90 [0.75; 1.09]				0.75 [0.63; 0.88]
0.97 [0.80; 1.19]	**UPA 30mg o.d.**	0.95 [0.82; 1.10]					0.77 [0.69; 0.86]
0.92 [0.76; 1.12]	0.95 [0.82; 1.10]	**UPA 15mg o.d.**					0.81 [0.73; 0.90]
0.90 [0.75; 1.09]	0.93 [0.78; 1.11]	0.98 [0.83; 1.16]	**GUS 200mg 4-weekly**				0.83 [0.72; 0.95]
0.85 [0.70; 1.04]	0.88 [0.74; 1.03]	0.92 [0.79; 1.08]	0.94 [0.79; 1.13]	**ETRO 105mg 4-weekly**			0.88 [0.78; 0.99]
0.80 [0.66; 0.97]	0.82 [0.70; 0.96]	0.87 [0.75; 1.00]	0.88 [0.75; 1.05]	0.94 [0.80; 1.10]	**RIS 180mg 8-weekly**	0.96 [0.86; 1.07]	0.94 [0.84; 1.04]
0.77 [0.63; 0.93]	0.79 [0.68; 0.91]	0.83 [0.72; 0.95]	0.85 [0.72; 1.00]	0.90 [0.77; 1.04]	0.96 [0.86; 1.07]	**RIS 360mg 8-weekly**	0.98 [0.89; 1.07]
0.75 [0.63; 0.88]	0.77 [0.69; 0.86]	0.81 [0.73; 0.90]	0.83 [0.72; 0.95]	0.88 [0.78; 0.99]	0.94 [0.84; 1.04]	0.98 [0.89; 1.07]	**PLA**

Relative risk with 95% confidence intervals in parentheses. Comparisons, column versus row, should be read from left to right, and are ordered relative to their overall efficacy. The intervention in the top left position is ranked as best after the network meta-analysis of direct and indirect effects. Direct comparisons are provided above the drug labels, and indirect comparisons are below. Boxes shaded green denote a statistically significant difference.

ETRO: etrolizumab, GUS: guselkumab, PLA: placebo; RIS: risankizumab; UPA: upadacitinib.

Supplementary Table 17. League Table for Failure to Achieve Corticosteroid Free-Remission in Trials Re-randomising Patients with UC Naïve to Advanced Therapies.

**UPA 30mg o.d.**						0.72 [0.32; 1.59]			0.58 [0.27; 1.25]
0.92 [0.32; 2.67]	**FIL 200mg o.d.**						0.72 [0.34; 1.52]		0.63 [0.30; 1.31]
0.81 [0.23; 2.78]	0.88 [0.26; 2.95]	**VED 300mg 8-weekly**							0.71 [0.27; 1.87]
0.75 [0.28; 1.97]	0.82 [0.32; 2.07]	0.93 [0.30; 2.85]	**GOL 100mg 4-weekly**		1.07 [0.53; 2.17]				0.77 [0.43; 1.37]
0.75 [0.26; 2.13]	0.81 [0.29; 2.25]	0.92 [0.28; 3.05]	1.00 [0.40; 2.48]	**IFX 120mg 2-weekly**					0.77 [0.38; 1.57]
0.72 [0.26; 2.03]	0.79 [0.29; 2.14]	0.90 [0.28; 2.91]	0.97 [0.49; 1.90]	0.97 [0.37; 2.58]	**GOL 50mg 4-weekly**				0.88 [0.44; 1.78]
0.72 [0.32; 1.59]	0.78 [0.28; 2.20]	0.89 [0.27; 2.97]	0.96 [0.38; 2.43]	0.96 [0.35; 2.66]	0.99 [0.37; 2.68]	**UPA 15mg o.d.**			0.80 [0.39; 1.67]
0.66 [0.23; 1.89]	0.72 [0.34; 1.52]	0.82 [0.25; 2.70]	0.89 [0.36; 2.20]	0.89 [0.33; 2.41]	0.92 [0.35; 2.42]	0.92 [0.34; 2.55]	**FIL 100mg o.d.**		0.87 [0.43; 1.76]
0.65 [0.23; 1.84]	0.71 [0.26; 1.94]	0.80 [0.24; 2.64]	0.87 [0.35; 2.14]	0.87 [0.32; 2.35]	0.90 [0.34; 2.36]	0.90 [0.33; 2.48]	0.98 [0.36; 2.63]	**ETRO 105mg 4-weekly**	0.89 [0.44; 1.79]
0.58 [0.27; 1.25]	0.63 [0.30; 1.31]	0.71 [0.27; 1.87]	0.77 [0.43; 1.37]	0.77 [0.38; 1.57]	0.80 [0.41; 1.56]	0.80 [0.39; 1.67]	0.87 [0.43; 1.76]	0.89 [0.44; 1.79]	**PLA**

Relative risk with 95% confidence intervals in parentheses. Comparisons, column versus row, should be read from left to right, and are ordered relative to their overall efficacy. The intervention in the top left position is ranked as best after the network meta-analysis of direct and indirect effects. Direct comparisons are provided above the drug labels, and indirect comparisons are below. Boxes shaded green denote a statistically significant difference.

ETRO: etrolizumab, FIL: filgotinib; GOL: golimumab; IFX: infliximab; PLA: placebo; UPA: upadacitinib, VED: vedolizumab.

Supplementary Table 18. League Table for Failure to Achieve Corticosteroid Free-Remission in Trials Re-randomising Patients with UC Exposed to Advanced Therapies.

**UPA 30mg o.d.**		0.67 [0.47; 0.94]					0.49 [0.36; 0.66]
0.81 [0.28; 2.41]	**VED 300mg 8-weekly**						0.60 [0.21; 1.70]
0.67 [0.47; 0.94]	0.82 [0.28; 2.36]	**UPA 15mg o.d.**					0.73 [0.60; 0.89]
0.64 [0.32; 1.26]	0.78 [0.23; 2.62]	0.95 [0.50; 1.81]	**IFX 120mg 2-weekly**				0.77 [0.42; 1.42]
0.54 [0.39; 0.76]	0.67 [0.23; 1.91]	0.82 [0.65; 1.03]	0.86 [0.46; 1.60]	**FIL 200mg o.d.**		0.93 [0.80; 1.07]	0.90 [0.79; 1.02]
0.53 [0.38; 0.75]	0.66 [0.23; 1.88]	0.80 [0.63; 1.03]	0.84 [0.45; 1.58]	0.98 [0.80; 1.20]	**ETRO 105mg 4-weekly**		0.91 [0.78; 1.07]
0.50 [0.37; 0.69]	0.62 [0.22; 1.77]	0.76 [0.61; 0.94]	0.79 [0.43; 1.48]	0.93 [0.80; 1.07]	0.94 [0.78; 1.14]	**FIL 100mg o.d.**	0.97 [0.87; 1.08]
0.49 [0.36; 0.66]	0.60 [0.21; 1.70]	0.73 [0.60; 0.89]S	0.77 [0.42; 1.42]	0.90 [0.79; 1.02]	0.91 [0.78; 1.07]	0.97 [0.87; 1.08]	**PLA**

Relative risk with 95% confidence intervals in parentheses. Comparisons, column versus row, should be read from left to right, and are ordered relative to their overall efficacy. The intervention in the top left position is ranked as best after the network meta-analysis of direct and indirect effects. Direct comparisons are provided above the drug labels, and indirect comparisons are below. Boxes shaded green denote a statistically significant difference.

ETRO: etrolizumab, FIL: filgotinib; IFX: infliximab; PLA: placebo; UPA: upadacitinib; VED: vedolizumab.

Supplementary Table 19. League Table for Failure to Achieve Histological-endoscopic Mucosal Improvement in Trials Re-randomising Patients with UC.

**UPA 30mg o.d.**		0.74 [0.61; 0.88]							0.50 [0.43; 0.58]
0.79 [0.64; 0.99]	**GUS 200mg 4-weekly**		0.92 [0.77; 1.11]						0.63 [0.54; 0.73]
0.74 [0.61; 0.88]	0.92 [0.76; 1.12]	**UPA 15mg o.d.**							0.68 [0.60; 0.76]
0.73 [0.60; 0.90]	0.92 [0.77; 1.11]	1.00 [0.83; 1.20]	**GUS 100mg 8-weekly**						0.68 [0.59; 0.78]
0.69 [0.56; 0.86]	0.87 [0.70; 1.08]	0.94 [0.77; 1.15]	0.94 [0.76; 1.16]	**UST 90mg 8-weekly**				0.89 [0.75; 1.07]	0.72 [0.62; 0.84]
0.67 [0.54; 0.83]	0.84 [0.68; 1.04]	0.91 [0.75; 1.10]	0.91 [0.74; 1.12]	0.97 [0.78; 1.20]	**RIS 180mg 8-weekly**	0.99 [0.83; 1.18]			0.74 [0.64; 0.87]
0.66 [0.54; 0.82]	0.83 [0.67; 1.03]	0.90 [0.75; 1.09]	0.90 [0.74; 1.11]	0.96 [0.77; 1.19]	0.99 [0.83; 1.18]	**RIS 360mg 8-weekly**			0.75 [0.65; 0.87]
0.65 [0.53; 0.78]	0.81 [0.67; 0.98]	0.88 [0.75; 1.03]	0.88 [0.73; 1.05]	0.93 [0.77; 1.13]	0.97 [0.80; 1.16]	0.97 [0.81; 1.17]	**IFX 120mg 2-weekly**		0.77 [0.69; 0.86]
0.62 [0.50; 0.76]	0.78 [0.63; 0.96]	0.84 [0.70; 1.01]	0.84 [0.69; 1.03]	0.89 [0.75; 1.07]	0.93 [0.75; 1.14]	0.93 [0.76; 1.15]	0.96 [0.80; 1.15]	**UST 90mg 12-weekly**	0.80 [0.70; 0.93]
0.50 [0.43; 0.58]	0.63 [0.54; 0.73]	0.68 [0.60; 0.76]	0.68 [0.59; 0.78]	0.72 [0.62; 0.84]	0.74 [0.64; 0.87]	0.75 [0.65; 0.87]	0.77 [0.69; 0.86]	0.80 [0.70; 0.93]	**PLA**

Relative risk with 95% confidence intervals in parentheses. Comparisons, column versus row, should be read from left to right, and are ordered relative to their overall efficacy. The intervention in the top left position is ranked as best after the network meta-analysis of direct and indirect effects. Direct comparisons are provided above the drug labels, and indirect comparisons are below. Boxes shaded green denote a statistically significant difference.

GUS: guselkumab; IFX: infliximab; PLA: placebo; RIS: risankizumab; UPA: upadacitinib; UST: ustekinumab.

Supplementary Table 20. League Table for Failure to Achieve Histological-endoscopic Mucosal Remission in Trials Re-randomising Patients with UC.

**MIR 200mg 4-weekly**								0.73 [0.64; 0.82]
0.95 [0.81; 1.11]	**FIL 200mg o.d.**				0.85 [0.75; 0.96]			0.77 [0.69; 0.85]
S0.89 [0.78; 1.03]	0.94 [0.83; 1.08]	**UPA 30mg o.d.**		0.95 [0.86; 1.04]				0.81 [0.75; 0.88]
0.88 [0.76; 1.03]	0.94 [0.81; 1.08]	0.99 [0.87; 1.12]	**OZA 1mg o.d.**					0.82 [0.74; 0.91]
0.85 [0.74; 0.97]	0.90 [0.79; 1.02]	0.95 [0.86; 1.04]	0.96 [0.85; 1.08]	**UPA 15mg o.d.**				0.86 [0.80; 0.92]
0.80 [0.69; 0.93]	0.85 [0.75; 0.96]	0.90 [0.80; 1.01]	0.91 [0.79; 1.04]	0.95 [0.85; 1.06]	**FIL 100mg o.d.**			0.90 [0.83; 0.99]
0.77 [0.67; 0.89]	0.82 [0.72; 0.94]	0.87 [0.78; 0.97]	0.88 [0.77; 0.99]	0.91 [0.82; 1.01]	0.97 [0.86; 1.09]	**RIS 360mg 8-weekly**	0.97 [0.89; 1.05]	0.94 [0.87; 1.01]
0.75 [0.65; 0.86]	0.79 [0.70; 0.90]	0.84 [0.76; 0.93]	0.85 [0.75; 0.96]	0.89 [0.80; 0.98]	0.93 [0.83; 1.05]	0.97 [0.89; 1.05]	**RIS 180mg 8-weekly**	0.97 [0.90; 1.04]
0.73 [0.64; 0.82]	0.77 [0.69; 0.85]	0.81 [0.75; 0.88]	0.82 [0.74; 0.91]	0.86 [0.80; 0.92]	0.90 [0.83; 0.99]	0.94 [0.87; 1.01]	0.97 [0.90; 1.04]	**PLA**

Relative risk with 95% confidence intervals in parentheses. Comparisons, column versus row, should be read from left to right, and are ordered relative to their overall efficacy. The intervention in the top left position is ranked as best after the network meta-analysis of direct and indirect effects. Direct comparisons are provided above the drug labels, and indirect comparisons are below. Boxes shaded green denote a statistically significant difference.

FIL: filgotinib; MIR: mirikizumab; OZA: ozanimod; PLA: placebo; RIS: risankizumab; UPA: upadacitinib.

Supplementary Table 21. League Table for Failure to Achieve Histological Remission in Trials Re-randomising Patients with UC.

**GUS 200mg 4-weekly**	0.96 [0.75; 1.23]								0.54 [0.44; 0.66]	
0.96 [0.75; 1.23]	**GUS 100mg 8-weekly**								0.56 [0.46; 0.68]	
0.73 [0.58; 0.92]	0.76 [0.61; 0.96]	**FIL 200mg o.d.**			0.85 [0.74; 0.98]				0.74 [0.65; 0.83]	
0.69 [0.55; 0.87]	0.72 [0.57; 0.90]	0.94 [0.79; 1.13]	**ETRO 105mg 4-weekly**						0.78 [0.69; 0.89]	
0.68 [0.54; 0.85]	0.70 [0.57; 0.88]	0.93 [0.79; 1.09]	0.98 [0.83; 1.16]	**OZA 1mg o.d.**					0.79 [0.71; 0.89]	
0.62 [0.50; 0.78]	0.65 [0.52; 0.81]	0.85 [0.74; 0.98]	0.90 [0.76; 1.07]	0.92 [0.79; 1.07]	**FIL 100mg o.d.**				0.86 [0.78; 0.96]	
0.58 [0.47; 0.73]	0.61 [0.49; 0.76]	0.80 [0.67; 0.94]	0.85 [0.71; 1.01]	0.86 [0.74; 1.01]	0.94 [0.80; 1.09]	**RIS 360mg 8-weekly**	1.00 [0.88; 1.13]		0.92 [0.82; 1.03]	
0.58 [0.47; 0.73]	0.61 [0.48; 0.76]	0.80 [0.67; 0.94]	0.85 [0.71; 1.01]	0.86 [0.73; 1.01]	0.94 [0.80; 1.09]	1.00 [0.88; 1.13]	**RIS 180mg 8-weekly**		0.92 [0.82; 1.04]	
0.54 [0.44; 0.66]	0.56 [0.46; 0.68]	0.74 [0.64; 0.84]	0.78 [0.68; 0.90]	0.80 [0.71; 0.90]	0.86 [0.77; 0.97]	0.92 [0.81; 1.05]	0.92 [0.81; 1.05]	**VED 300mg 8-weekly**	1.00 [0.95; 1.05]	0.98 [0.95; 1.02]
0.54 [0.44; 0.66]	0.56 [0.46; 0.68]	0.74 [0.65; 0.83]	0.78 [0.69; 0.89]	0.79 [0.71; 0.89]	0.86 [0.78; 0.96]	0.92 [0.82; 1.03]	0.92 [0.82; 1.04]	1.00 [0.95; 1.05]	**PLA**	0.98 [0.95; 1.02]
0.53 [0.43; 0.65]	0.55 [0.45; 0.67]	0.72 [0.64; 0.82]	0.77 [0.67; 0.88]	0.78 [0.70; 0.87]	0.85 [0.76; 0.95]	0.91 [0.80; 1.02]	0.91 [0.80; 1.02]	0.98 [0.95; 1.02]	0.98 [0.95; 1.02]	**VED 108mg S/C 2-weekly**

Relative risk with 95% confidence intervals in parentheses. Comparisons, column versus row, should be read from left to right, and are ordered relative to their overall efficacy. The intervention in the top left position is ranked as best after the network meta-analysis of direct and indirect effects. Direct comparisons are provided above the drug labels, and indirect comparisons are below. Boxes shaded green denote a statistically significant difference.

ETRO: etrolizumab, FIL: filgotinib; GOL: golimumab; GUS: guselkumab; OZA: ozanimod; PLA: placebo; RIS: risankizumab; VED: vedolizumab.

Supplementary Table 22. League Table for Failure to Achieve Clinical Remission in Trials Treating Patients with UC Through.

**ETRA 2mg o.d.**							0.73 [0.64; 0.83]
0.96 [0.81; 1.15]	**IFX 10mg/kg 8-weekly**			0.93 [0.81; 1.07]			0.75 [0.66; 0.85]
0.94 [0.77; 1.15]	0.98 [0.80; 1.19]	**VED 300mg 8-weekly**				0.89 [0.78; 1.01]	
0.90 [0.75; 1.08]	0.94 [0.79; 1.12]	0.96 [0.79; 1.17]	**OZA 1mg o.d.**				0.81 [0.71; 0.92]
0.88 [0.75; 1.02]	0.91 [0.80; 1.04]	0.93 [0.78; 1.11]	0.97 [0.84; 1.13]	**IFX 5mg/kg 8-weekly**	0.95 [0.83; 1.10]		0.83 [0.77; 0.91]
0.84 [0.68; 1.03]	0.87 [0.72; 1.05]	0.89 [0.71; 1.11]	0.93 [0.76; 1.14]	0.95 [0.83; 1.10]	**ETRO 105mg 4-weekly**		
0.84 [0.72; 0.98]	0.87 [0.75; 1.01]	0.89 [0.78; 1.01]	0.93 [0.80; 1.08]	0.95 [0.85; 1.08]	1.00 [0.83; 1.20]	**ADA 40mg 2-weekly**	0.87 [0.80; 0.95]
0.73 [0.64; 0.83]	0.76 [0.67; 0.86]	0.78 [0.67; 0.90]	0.81 [0.71; 0.92]	0.83 [0.77; 0.91]	0.87 [0.74; 1.03]	0.87 [0.80; 0.95]	**PLA**

Relative risk with 95% confidence intervals in parentheses. Comparisons, column versus row, should be read from left to right, and are ordered relative to their overall efficacy. The intervention in the top left position is ranked as best after the network meta-analysis of direct and indirect effects. Direct comparisons are provided above the drug labels, and indirect comparisons are below. Boxes shaded green denote a statistically significant difference.

ADA: adalimumab, ETRA: etrasimod, ETRO: etrolizumab, IFX: infliximab; OZA: ozanimod; PLA: placebo; VED: vedolizumab.

Supplementary Table 23. Confidence in Network Meta-Analysis Framework Evaluating the Confidence in the Indirect and Direct Treatment Estimates From the Network for Failure to Achieve Clinical Remission in Trials Treating Patients with UC Through.

Comparison	No. of studies	Within-study bias	Reporting bias	Indirectness	Imprecision	Heterogeneity	Incoherence	Confidence rating	Reason(s) for downgrading
**DIRECT EVIDENCE**									
Adalimumab 40mg 2-weekly: Placebo	2	Some concerns	Low risk	No concerns	No concerns	Some concerns	No concerns	Moderate	Within-study bias; Heterogeneity
Adalimumab 40mg 2-weekly: Vedolizumab 300mg 8-weekly	1	Major concerns	Low risk	No concerns	Some concerns	Some concerns	No concerns	Low	Within-study bias; Imprecision; Heterogeneity
Etrasimod 2mg o.d.: Placebo	1	No concerns	Low risk	No concerns	No concerns	No concerns	No concerns	High	NA
Etrolizumab 105mg 4-weekly: Infliximab 5mg/kg 8-weekly	1	No concerns	Low risk	No concerns	Major concerns	No concerns	No concerns	Moderate	Imprecision
Infliximab 10mg/kg 8-weekly: Infliximab 5mg/kg 8-weekly	2	No concerns	Low risk	No concerns	Some concerns	Some concerns	No concerns	Moderate	Imprecision; Heterogeneity
Infliximab 10mg/kg 8-weekly: Placebo	2	No concerns	Low risk	No concerns	No concerns	No concerns	No concerns	High	NA
Infliximab 5mg/kg 8-weekly: Placebo	5	No concerns	Low risk	No concerns	No concerns	No concerns	No concerns	High	NA
Ozanimod 1mg o.d.: Placebo	2	No concerns	Low risk	No concerns	No concerns	No concerns	No concerns	High	NA
**INDIRECT EVIDENCE**									
Adalimumab 40mg 2-weekly: Etrasimod 2mg o.d.	NA	Some concerns	Low risk	No concerns	No concerns	Some concerns	No concerns	Moderate	Within-study bias; Heterogeneity
Adalimumab 40mg 2-weekly: Etrolizumab 105mg 4-weekly	NA	No concerns	Low risk	No concerns	Major concerns	No concerns	No concerns	Moderate	Imprecision
Adalimumab 40mg 2-weekly: Infliximab 10mg/kg 8-weekly	NA	Some concerns	Low risk	No concerns	Some concerns	Some concerns	No concerns	Moderate	Within-study bias; Imprecision
Adalimumab 40mg 2-weekly: Infliximab 5mg/kg 8-weekly	NA	Some concerns	Low risk	No concerns	Major concerns	No concerns	No concerns	Moderate	Imprecision
Adalimumab 40mg 2-weekly: Ozanimod 1mg o.d.	NA	Some concerns	Low risk	No concerns	Major concerns	No concerns	No concerns	Moderate	Imprecision
Etrasimod 2mg o.d.: Etrolizumab 105mg 4-weekly	NA	No concerns	Low risk	No concerns	Some concerns	Some concerns	No concerns	Moderate	Imprecision; Heterogeneity
Etrasimod 2mg o.d.: Infliximab 10mg/kg 8-weekly	NA	No concerns	Low risk	No concerns	Major concerns	No concerns	No concerns	Moderate	Imprecision
Etrasimod 2mg o.d.: Infliximab 5mg/kg 8-weekly	NA	No concerns	Low risk	No concerns	Some concerns	Some concerns	No concerns	Moderate	Imprecision; Heterogeneity
Etrasimod 2mg o.d.: Ozanimod 1mg o.d.	NA	No concerns	Low risk	No concerns	Major concerns	No concerns	No concerns	Moderate	Imprecision

Etrasimod 2mg o.d.: Vedolizumab 300mg 8-weekly	NA	Some concerns	Low risk	No concerns	Major concerns	No concerns	No concerns	Moderate	Imprecision
Etrolizumab 105mg 4-weekly: Infliximab 10mg/kg 8-weekly	NA	No concerns	Low risk	No concerns	Major concerns	No concerns	No concerns	Moderate	Imprecision
Etrolizumab 105mg 4-weekly: Ozanimod 1mg o.d.	NA	No concerns	Low risk	No concerns	Major concerns	No concerns	No concerns	Moderate	Imprecision
Etrolizumab 105mg 4-weekly: Placebo	NA	No concerns	Low risk	No concerns	Some concerns	Some concerns	No concerns	Moderate	Imprecision; Heterogeneity
Etrolizumab 105mg 4-weekly: Vedolizumab 300mg 8-weekly	NA	Some concerns	Low risk	No concerns	Major concerns	No concerns	No concerns	Moderate	Imprecision
Infliximab 10mg/kg 8-weekly: Ozanimod 1mg o.d.	NA	No concerns	Low risk	No concerns	Major concerns	No concerns	No concerns	Moderate	Imprecision
Infliximab 10mg/kg 8-weekly: Vedolizumab 300mg 8-weekly	NA	Some concerns	Low risk	No concerns	Major concerns	No concerns	No concerns	Moderate	Imprecision
Infliximab 5mg/kg 8-weekly: Ozanimod 1mg o.d.	NA	No concerns	Low risk	No concerns	Major concerns	No concerns	No concerns	Moderate	Imprecision
Infliximab 5mg/kg 8-weekly: Vedolizumab 300mg 8-weekly	NA	Some concerns	Low risk	No concerns	Major concerns	No concerns	No concerns	Moderate	Imprecision
Ozanimod 1mg o.d.: Vedolizumab 300mg 8-weekly	NA	Some concerns	Low risk	No concerns	Major concerns	No concerns	No concerns	Moderate	Imprecision
Placebo: Vedolizumab 300mg 8-weekly	NA	Major concerns	Low risk	No concerns	No concerns	No concerns	No concerns	Moderate	Within-study bias
Adalimumab 40mg 2-weekly: Etrasimod 2mg o.d.	NA	Some concerns	Low risk	No concerns	No concerns	Some concerns	No concerns	Moderate	Within-study bias; Heterogeneity

This table shows the confidence rating of evidence for all direct and indirect comparisons across the network.

Within-study bias: This relates to the risk of bias assessment made for each included study. The studies' contributions are combined with the risk of bias judgments to evaluate within-study bias for each estimate from a network meta-analysis.

Reporting bias: This relates to the assessment of the risk of bias from missing data or incomplete reporting.

Indirectness: Each study included in the network is evaluated according to its relevance to the research question, classified into low, moderate, or high indirectness.

Imprecision: The evaluation of imprecision requires that the relative treatment effect representing a clinically important difference is defined. We set this at 0.05 which creates a range of equivalence between 0.95 and 1.05. The treatment effect of the 95% CI of each comparison is

compared with the range of equivalence. A rating of “major concerns” is given if the 95% CI extends beyond the range of equivalence on the opposite side of the null effect line as the point estimate, i.e., compatible with clinically important treatment effects in both directions, and a rating of “some concerns” is given if the 95% CI extends into, but not beyond, the range of equivalence on the opposite side of the null effect line as the point estimate.

Heterogeneity: Network meta-analysis assumes a single heterogeneity variance across all comparisons, expressed as tau^2, and this can, in turn, be expressed as a prediction interval. The prediction interval shows where the true effect of a new study similar to the existing studies is expected to lie. The 95% CI of each comparison is compared to the prediction interval, with reference to the rang of equivalence. If both lead to the same conclusions, then there are “no concerns” regarding heterogeneity. A rating of “major concerns” is given if the prediction interval extends beyond the range of equivalence on the opposite side of the null effect line as the point estimate, i.e., compatible with clinically important treatment effects in both directions, and a rating of “some concerns” is given if the prediction interval extends into, but not beyond, the range of equivalence on the opposite side of the null effect line as the point estimate.

Incoherence: This evaluates the agreement between direct and indirect evidence for certain comparisons in the network (also referred to as inconsistency). Where the 95% CI of the direct and indirect treatment estimate for a comparison would lead to the same conclusion with reference to the range of equivalence, a rating of “no concerns” is given.

Overall confidence rating and process of downgrading confidence: The quality of evidence was downgraded by one level if there were “major concerns” in one area, or “some concerns” in two areas. Consequently, the overall confidence rating for each comparison was based on the additive effect of ratings across all assessment domains.

Supplementary Table 24. League Table for Failure to Achieve Clinical Remission in Trials Treating Patients with UC Naïve to Advanced Therapies Through.

**ETRA 2mg o.d.**						0.69 [0.60; 0.80]
0.92 [0.74; 1.15]	**VED 300mg 8-weekly**			0.87 [0.76; 1.00]		
0.91 [0.75; 1.10]	0.98 [0.80; 1.21]	**IFX 10mg/kg 8-weekly**	0.93 [0.81; 1.06]			0.75 [0.66; 0.85]
0.83 [0.70; 0.98]	0.89 [0.74; 1.08]	0.91 [0.80; 1.03]	**IFX 5mg/kg 8-weekly**		0.95 [0.83; 1.09]	0.83 [0.77; 0.91]
0.80 [0.67; 0.96]	0.87 [0.76; 1.00]	0.88 [0.76; 1.03]	0.97 [0.86; 1.10]	**ADA 40mg 2-weekly**		0.86 [0.78; 0.94]
0.79 [0.64; 0.98]	0.85 [0.68; 1.07]	0.87 [0.72; 1.05]	0.95 [0.83; 1.09]	0.98 [0.82; 1.18]	**ETRO 105mg 4-weekly**	
0.69 [0.60; 0.80]	0.75 [0.63; 0.88]	0.76 [0.67; 0.85]	0.83 [0.77; 0.91]	0.86 [0.78; 0.94]	0.87 [0.75; 1.02]	**PLA**

Relative risk with 95% confidence intervals in parentheses. Comparisons, column versus row, should be read from left to right, and are ordered relative to their overall efficacy. The intervention in the top left position is ranked as best after the network meta-analysis of direct and indirect effects. Direct comparisons are provided above the drug labels, and indirect comparisons are below. Boxes shaded green denote a statistically significant difference.

ADA: adalimumab, ETRA: etrasimod, ETRO: etrolizumab, IFX: infliximab; PLA: placebo; VED: vedolizumab.

Supplementary Table 25. League Table for Failure to Achieve Clinical Remission in Trials Treating Patients with UC Exposed to Advanced Therapies Through.

**ETRA 2mg o.d.**			0.83 [0.72; 0.95]
0.94 [0.76; 1.17]	**VED 300mg 8-weekly**	0.95 [0.82; 1.10]	
0.90 [0.76; 1.05]	0.95 [0.82; 1.10]	**ADA 40mg 2-weekly**	0.93 [0.86; 1.00]
0.83 [0.72; 0.95]	0.88 [0.75; 1.04]	0.93 [0.86; 1.00]	**PLA**

Relative risk with 95% confidence intervals in parentheses. Comparisons, column versus row, should be read from left to right, and are ordered relative to their overall efficacy. The intervention in the top left position is ranked as best after the network meta-analysis of direct and indirect effects. Direct comparisons are provided above the drug labels, and indirect comparisons are below. Boxes shaded green denote a statistically significant difference.

ADA: adalimumab, ETRA: etrasimod, PLA: placebo; VED: vedolizumab.

Supplementary Table 26. League Table for Failure to Achieve Endoscopic Improvement in Trials Treating Patients with UC Through.

**IFX 10mg/kg 8-weekly**			0.90 [0.75; 1.07]				0.64 [0.55; 0.74]
0.92 [0.77; 1.10]	**ETRA 2mg o.d.**						0.70 [0.63; 0.78]
0.88 [0.73; 1.06]	0.96 [0.81; 1.13]	**VED 300mg 8-weekly**				0.84 [0.76; 0.93]	
0.87 [0.74; 1.02]	0.95 [0.81; 1.10]	0.99 [0.84; 1.16]	**IFX 5mg/kg 8-weekly**		0.93 [0.82; 1.06]		0.74 [0.67; 0.82]
0.84 [0.66; 1.07]	0.92 [0.73; 1.14]	0.96 [0.76; 1.20]	0.97 [0.78; 1.20]	**OZA 1mg o.d.**			0.77 [0.63; 0.93]
0.81 [0.66; 0.99]	0.88 [0.72; 1.07]	0.92 [0.75; 1.13]	0.93 [0.82; 1.06]	0.96 [0.75; 1.23]	**ETRO 105mg 4-weekly**		
0.74 [0.63; 0.86]	0.80 [0.70; 0.92]	0.84 [0.76; 0.93]	0.85 [0.75; 0.96]	0.88 [0.71; 1.07]	0.91 [0.76; 1.09]	**ADA 40mg 2-weekly**	0.88 [0.82; 0.94]
0.64 [0.56; 0.74]	0.70 [0.63; 0.78]	0.73 [0.65; 0.83]	0.74 [0.67; 0.82]	0.77 [0.63; 0.93]	0.80 [0.68; 0.94]	0.88 [0.82; 0.94]	**PLA**

Relative risk with 95% confidence intervals in parentheses. Comparisons, column versus row, should be read from left to right, and are ordered relative to their overall efficacy. The intervention in the top left position is ranked as best after the network meta-analysis of direct and indirect effects. Direct comparisons are provided above the drug labels, and indirect comparisons are below. Boxes shaded green denote a statistically significant difference.

ADA: adalimumab, ETRA: etrasimod, ETRO: etrolizumab, IFX: infliximab; OZA: ozanimod; PLA: placebo; VED: vedolizumab.

Supplementary Table 27. League Table for Failure to Achieve Endoscopic Improvement in Trials Treating Patients with UC Naïve to Advanced Therapies Through.

**IFX 10mg/kg 8-weekly**			0.90 [0.75; 1.07]			0.64 [0.55; 0.74]
0.94 [0.76; 1.16]	**VED 300mg 8-weekly**				0.81 [0.72; 0.91]	
0.93 [0.76; 1.14]	0.99 [0.80; 1.22]	**ETRA 2mg o.d.**				0.69 [0.60; 0.80]
0.87 [0.74; 1.02]	0.92 [0.77; 1.11]	0.93 [0.78; 1.11]	**IFX 5mg/kg 8-weekly**	0.93 [0.82; 1.06]		0.74 [0.67; 0.82]
0.81 [0.66; 0.99]	0.86 [0.69; 1.07]	0.87 [0.70; 1.08]	0.93 [0.82; 1.06]	**ETRO 105mg 4-weekly**		
0.76 [0.64; 0.90]	0.81 [0.72; 0.91]	0.82 [0.69; 0.97]	0.87 [0.76; 1.00]	0.94 [0.78; 1.14]	**ADA 40mg 2-weekly**	0.85 [0.77; 0.93]
0.64 [0.56; 0.74]	0.69 [0.59; 0.80]	0.69 [0.60; 0.80]	0.74 [0.67; 0.82]	0.80 [0.68; 0.94]	0.85 [0.77; 0.93]	**PLA**

Relative risk with 95% confidence intervals in parentheses. Comparisons, column versus row, should be read from left to right, and are ordered relative to their overall efficacy. The intervention in the top left position is ranked as best after the network meta-analysis of direct and indirect effects. Direct comparisons are provided above the drug labels, and indirect comparisons are below. Boxes shaded green denote a statistically significant difference.

ADA: adalimumab, ETRA: etrasimod, ETRO: etrolizumab, IFX: infliximab; PLA: placebo; VED: vedolizumab.

Supplementary Table 28. League Table for Failure to Achieve Endoscopic Improvement in Trials Treating Patients with UC Exposed to Advanced Therapies Through.

**ETRA 2mg o.d.**			0.73 [0.62; 0.86]
0.83 [0.64; 1.08]	**VED 300mg 8-weekly**	0.93 [0.79; 1.11]	
0.77 [0.63; 0.94]	0.93 [0.79; 1.11]	**ADA 40mg 2-weekly**	0.94 [0.85; 1.05]
0.73 [0.62; 0.86]	0.88 [0.72; 1.07]	0.94 [0.85; 1.05]	**PLA**

Relative risk with 95% confidence intervals in parentheses. Comparisons, column versus row, should be read from left to right, and are ordered relative to their overall efficacy. The intervention in the top left position is ranked as best after the network meta-analysis of direct and indirect effects. Direct comparisons are provided above the drug labels, and indirect comparisons are below. Boxes shaded green denote a statistically significant difference.

ADA: adalimumab, ETRA: etrasimod, PLA: placebo; VED: vedolizumab.

Supplementary Table 29. League Table for Failure to Achieve Corticosteroid-Free Remission in Trials Treating Patients Through.

**ETRA 2mg o.d.**					0.75 [0.62; 0.90]	
0.91 [0.74; 1.12]	**IFX 5mg/kg 8-weekly**	0.98 [0.83; 1.14]	0.99 [0.86; 1.14]		0.82 [0.75; 0.90]	
0.89 [0.68; 1.15]	0.98 [0.83; 1.14]	**ETRO 105mg 4-weekly**				
0.88 [0.71; 1.10]	0.97 [0.85; 1.11]	0.99 [0.81; 1.22]	**IFX 10mg/kg 8-weekly**		0.84 [0.75; 0.95]	
0.79 [0.65; 0.97]	0.87 [0.77; 0.99]	0.89 [0.73; 1.09]	0.90 [0.78; 1.03]	**ADA 40mg 2-weekly**	0.94 [0.87; 1.02]	0.89 [0.77; 1.03]
0.75 [0.62; 0.90]	0.82 [0.75; 0.90]	0.84 [0.70; 1.01]	0.85 [0.76; 0.95]	0.94 [0.87; 1.02]	**PLA**	
0.71 [0.55; 0.91]	0.78 [0.64; 0.94]	0.80 [0.62; 1.02]	0.80 [0.66; 0.98]	0.89 [0.77; 1.03]	0.95 [0.81; 1.12]	**VED 300mg 8-weekly**

Relative risk with 95% confidence intervals in parentheses. Comparisons, column versus row, should be read from left to right, and are ordered relative to their overall efficacy. The intervention in the top left position is ranked as best after the network meta-analysis of direct and indirect effects. Direct comparisons are provided above the drug labels, and indirect comparisons are below. Boxes shaded green denote a statistically significant difference.

ADA: adalimumab, ETRA: etrasimod, ETRO: etrolizumab, IFX: infliximab; PLA: placebo; VED: vedolizumab.

Supplementary Table 30. League Table for Failure to Achieve Corticosteroid-Free Remission in Trials Treating Patients with UC Naïve to Advanced Therapies Through.

**IFX 5mg/kg 8-weekly**	0.99 [0.86; 1.14]	0.98 [0.83; 1.14]			0.82 [0.75; 0.90]
0.97 [0.85; 1.11]	**IFX 10mg/kg 8-weekly**				0.84 [0.75; 0.95]
0.98 [0.83; 1.14]	1.01 [0.82; 1.23]	**ETRO 105mg 4-weekly**			
0.88 [0.78; 1.00]	0.91 [0.79; 1.05]	0.91 [0.74; 1.11]	**ADA 40mg 2-weekly**	0.92 [0.78; 1.08]	0.93 [0.86; 1.01]
0.81 [0.66; 1.00]	0.84 [0.68; 1.04]	0.83 [0.64; 1.08]	0.92 [0.78; 1.08]	**VED 300mg 8-weekly**	
0.82 [0.75; 0.90]	0.85 [0.76; 0.95]	0.84 [0.70; 1.01]	0.93 [0.86; 1.01]	1.01 [0.84; 1.21]	**PLA**

Relative risk with 95% confidence intervals in parentheses. Comparisons, column versus row, should be read from left to right, and are ordered relative to their overall efficacy. The intervention in the top left position is ranked as best after the network meta-analysis of direct and indirect effects. Direct comparisons are provided above the drug labels, and indirect comparisons are below. Boxes shaded green denote a statistically significant difference.

ADA: adalimumab, ETRO: etrolizumab, IFX: infliximab; PLA: placebo; VED: vedolizumab.

Supplementary Table 31. League Table for Failure to Achieve Corticosteroid-Free Remission in Trials Treating Patients with UC Exposed to Advanced Therapies Through.

**ADA 40mg 2-weekly**	0.98 [0.92; 1.04]	0.81 [0.65; 1.01]
0.98 [0.92; 1.04]	**PLA**	
0.81 [0.65; 1.01]	0.83 [0.66; 1.04]	**VED 300mg 8-weekly**

Relative risk with 95% confidence intervals in parentheses. Comparisons, column versus row, should be read from left to right, and are ordered relative to their overall efficacy. The intervention in the top left position is ranked as best after the network meta-analysis of direct and indirect effects. Direct comparisons are provided above the drug labels, and indirect comparisons are below. Boxes shaded green denote a statistically significant difference.

ADA: adalimumab, PLA: placebo; VED: vedolizumab.

SUPPLEMENTARY FIGURES

Supplementary Figure 1. Flow Diagram of Assessment of Studies Identified in the Systematic Review.

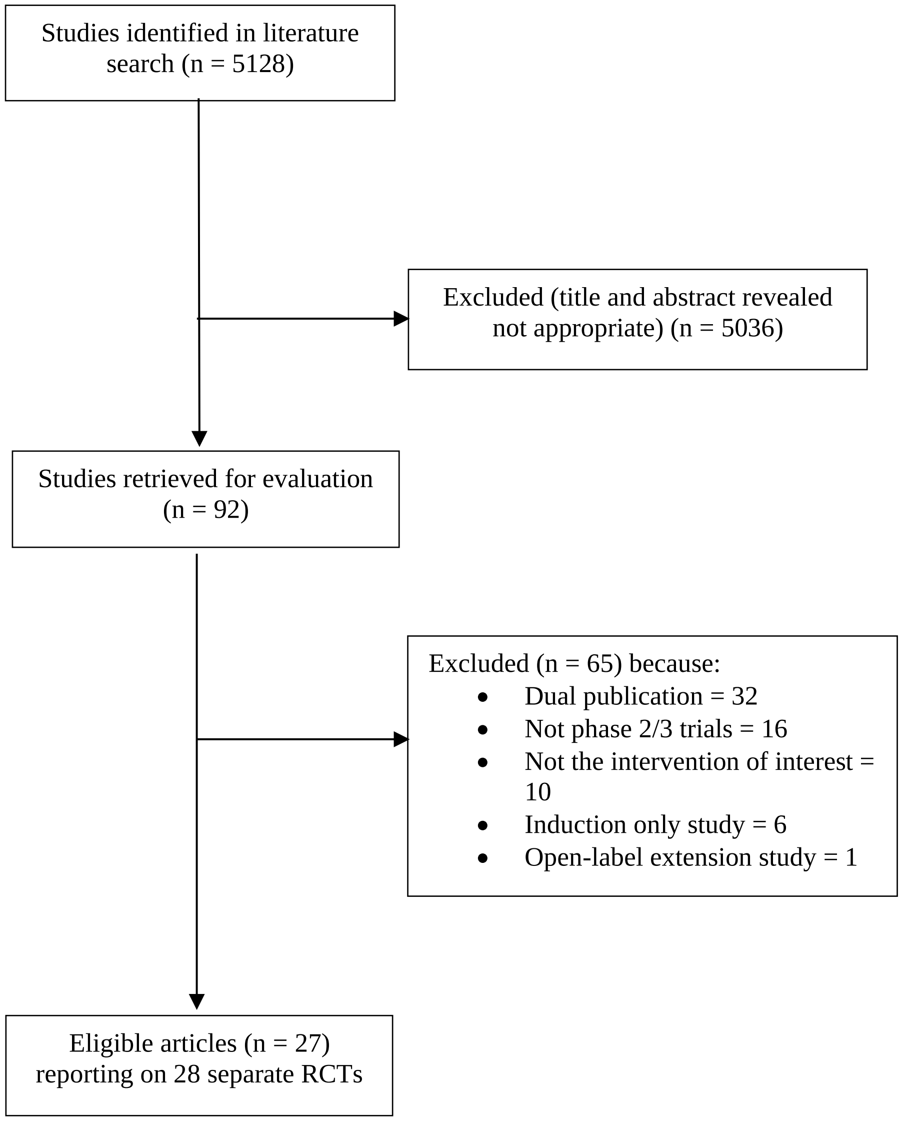

Supplementary Figure 2. Network Plot for Failure to Achieve Clinical Remission in Trials Re-randomising Patients with UC.

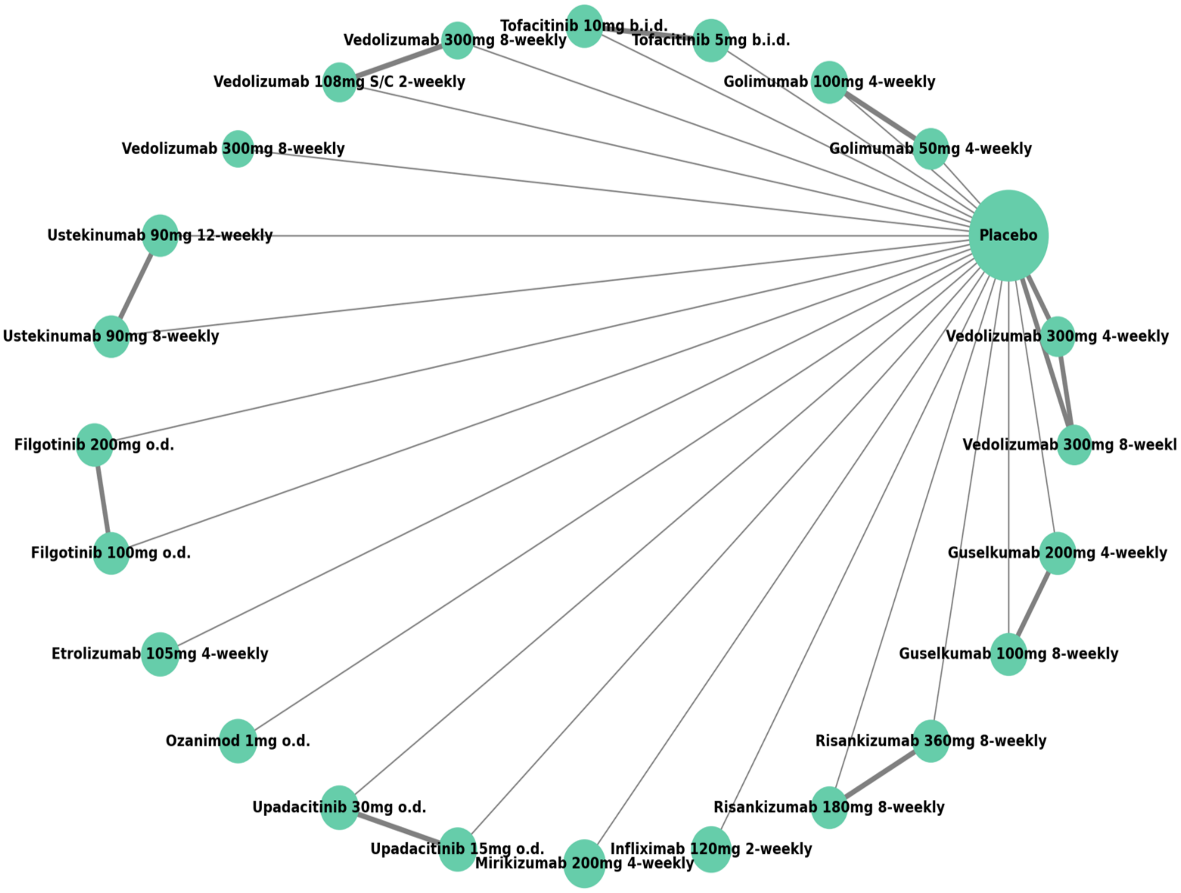

Note: Circle (node) size is proportional to the number of study participants assigned to receive each intervention. The line width (connection size) corresponds to the number of studies comparing the individual interventions.

Supplementary Figure 3. Funnel Plot for Failure to Achieve Clinical Remission in Trials Re-randomising Patients with UC.

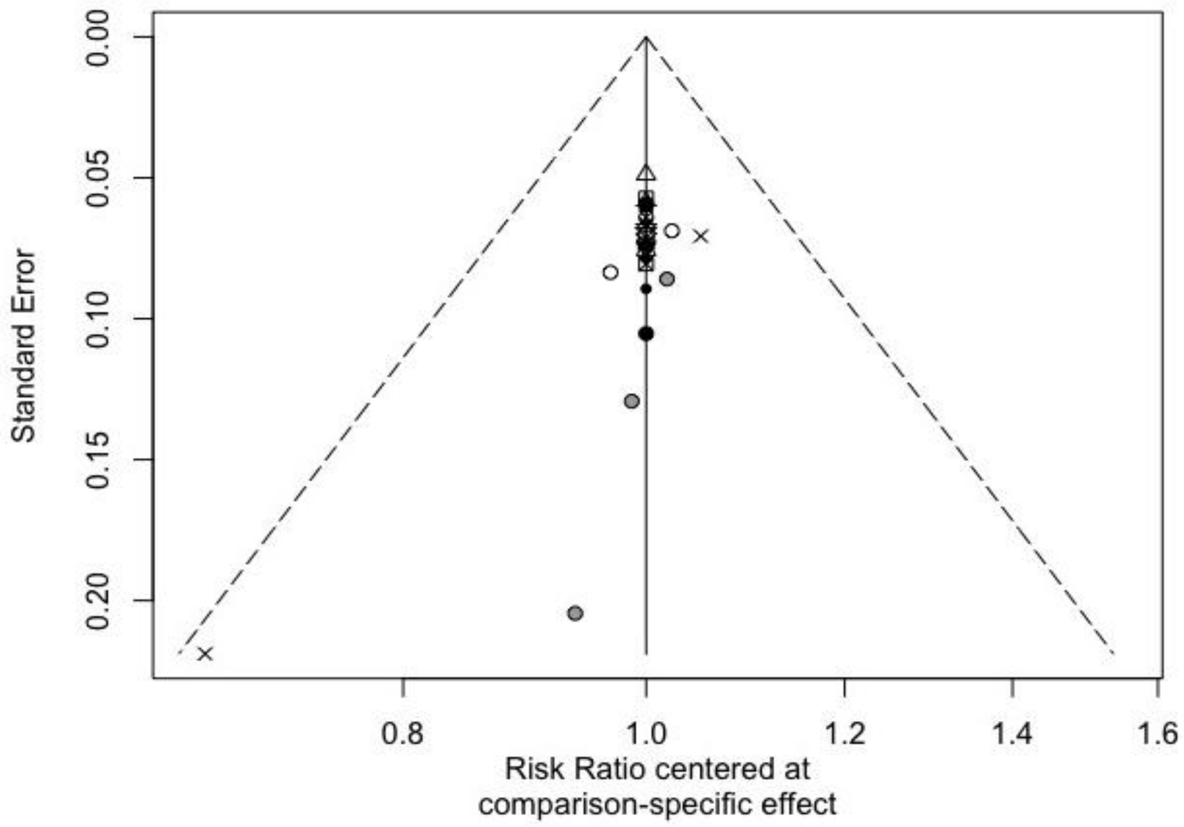

Supplementary Figure 4. Forest Plot for Failure to Achieve Clinical Remission in Trials Re-randomising Patients with UC Naïve to Advanced Therapies.

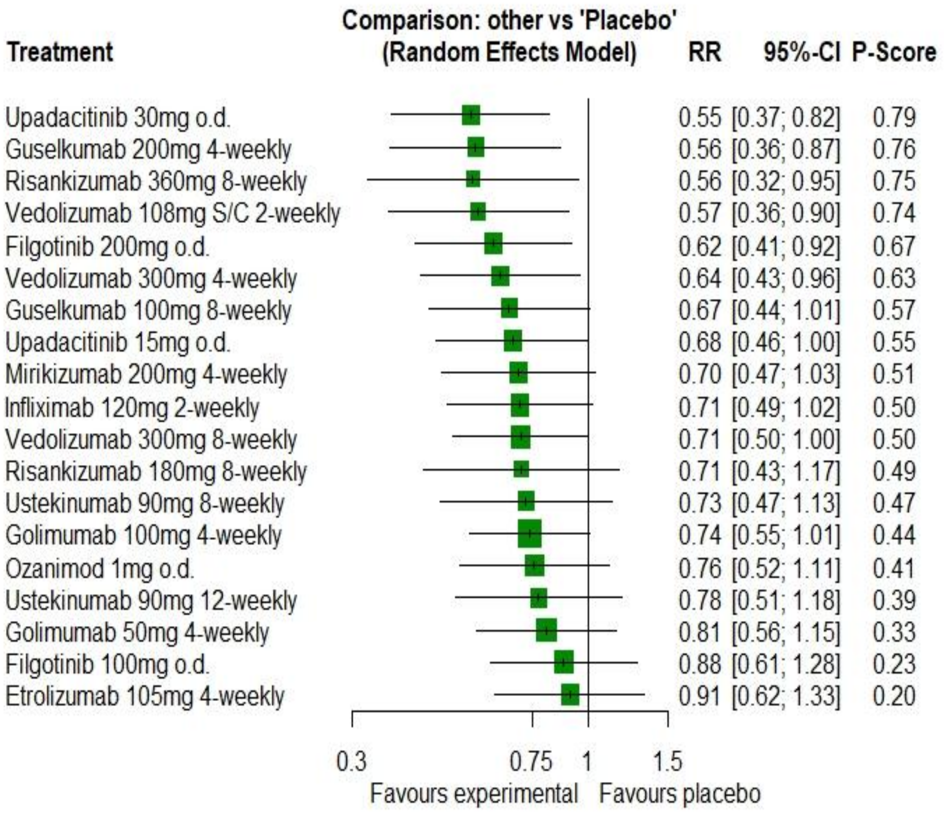

Note: The P-score is the probability of each intervention being ranked as best in the network.

Supplementary Figure 5. Forest Plot for Failure to Achieve Clinical Remission in Trials Re-randomising Patients with UC Exposed to Advanced Therapies.

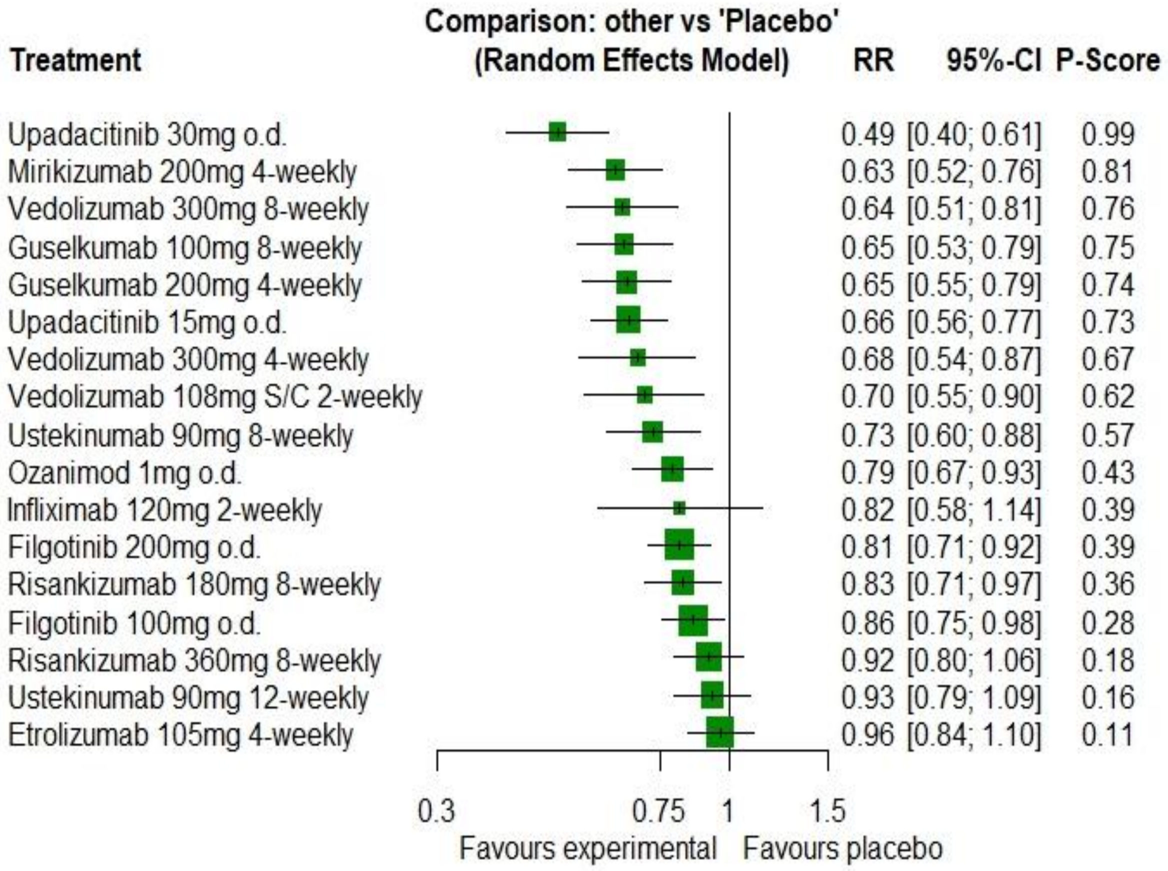

Note: The P-score is the probability of each intervention being ranked as best in the network.

Supplementary Figure 6. Network Plot for Failure to Achieve Endoscopic Improvement in Trials Re-randomising Patients with UC.

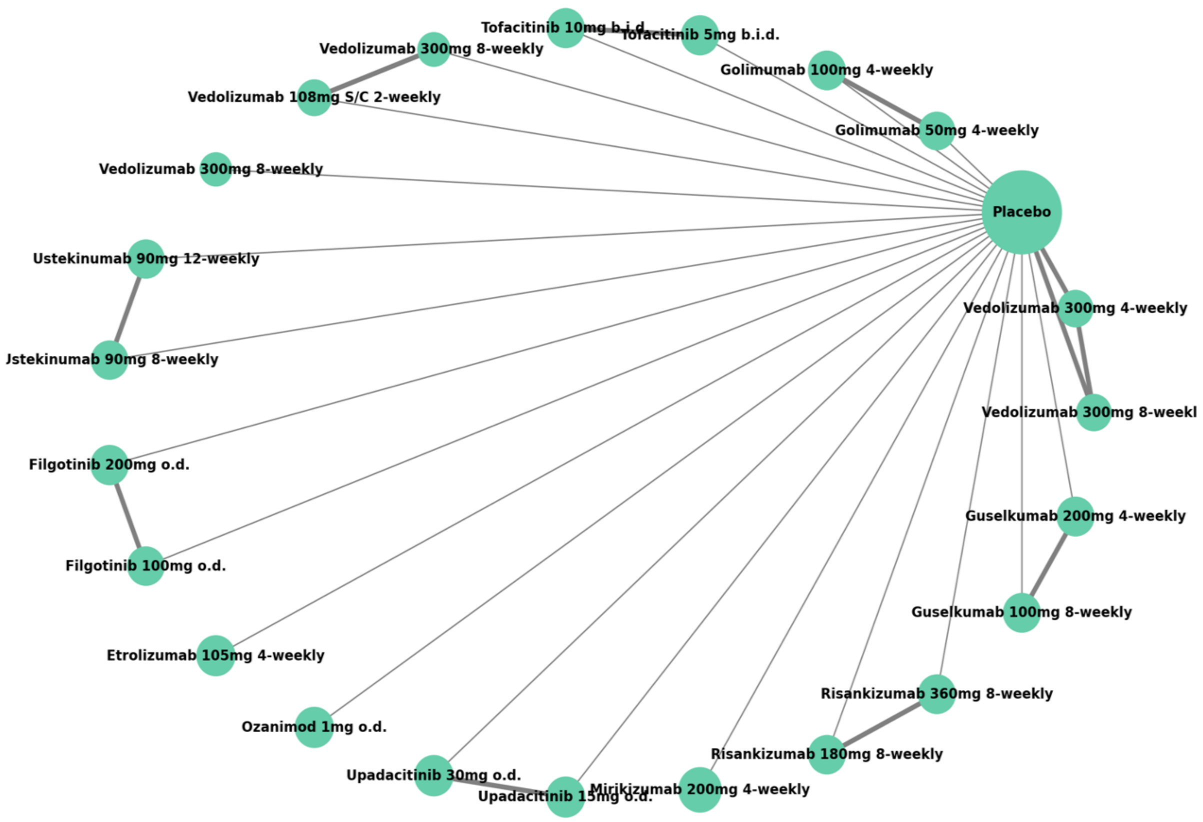

Note: Circle (node) size is proportional to the number of study participants assigned to receive each intervention. The line width (connection size) corresponds to the number of studies comparing the individual interventions.

Supplementary Figure 7. Funnel Plot for Failure to Achieve Endoscopic Improvement in Trials Re-randomising Patients with UC.

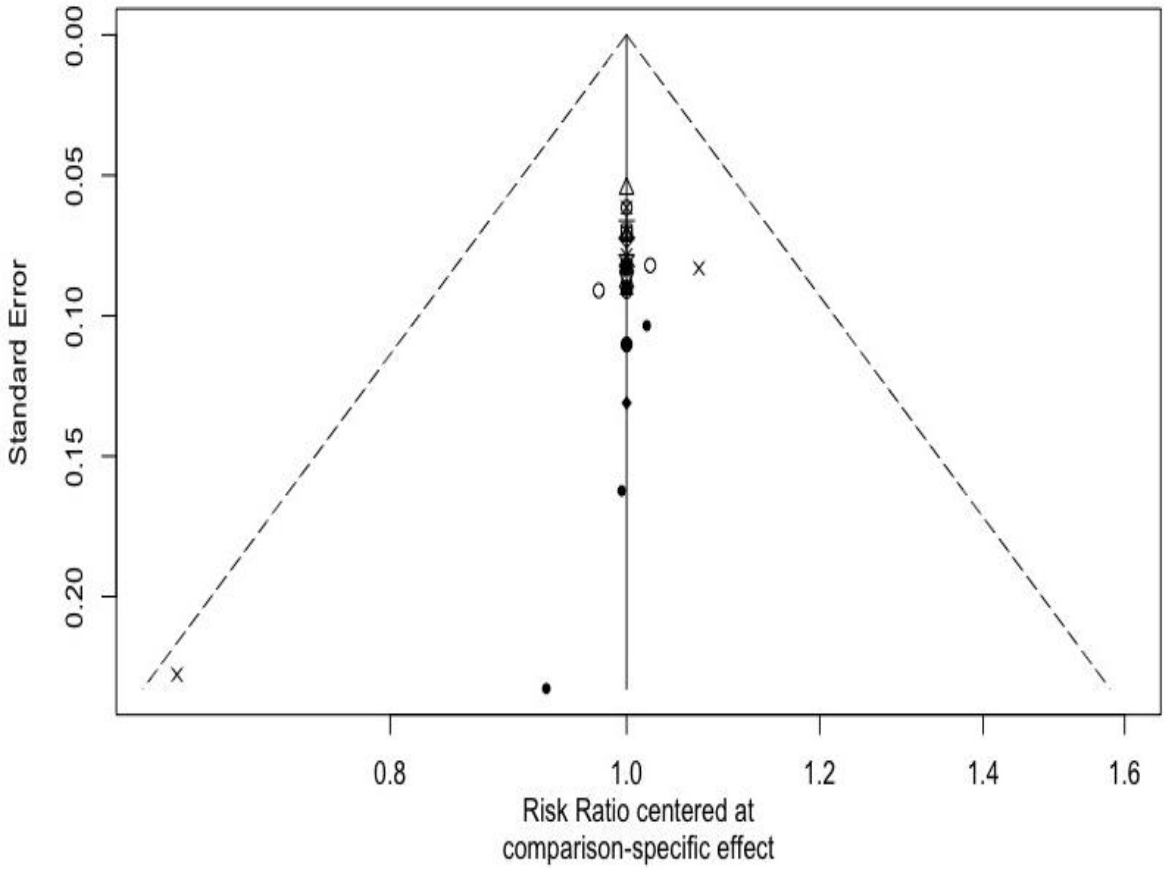

Supplementary Figure 8. Forest Plot for Failure to Achieve Endoscopic Improvement in Trials Re-randomising Patients with UC Naïve to Advanced Therapies.

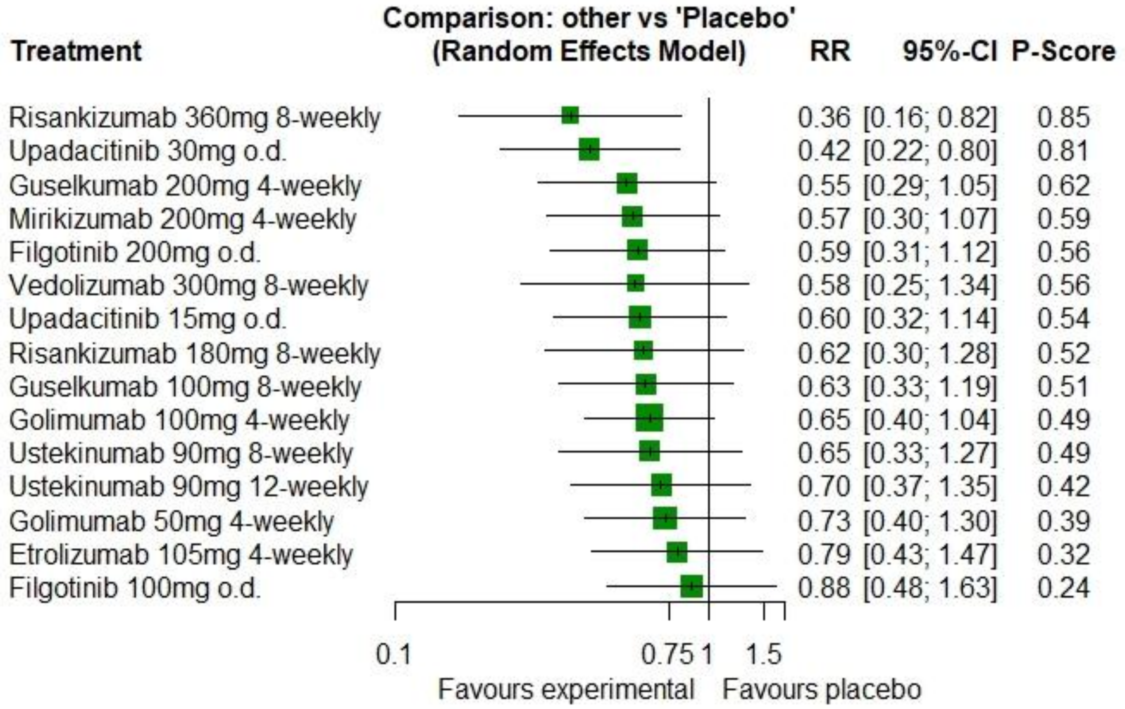

Note: The P-score is the probability of each intervention being ranked as best in the network.

Supplementary Figure 9. Forest Plot for Failure to Achieve Endoscopic Improvement in Trials Re-randomising Patients with UC Exposed to Advanced Therapies.

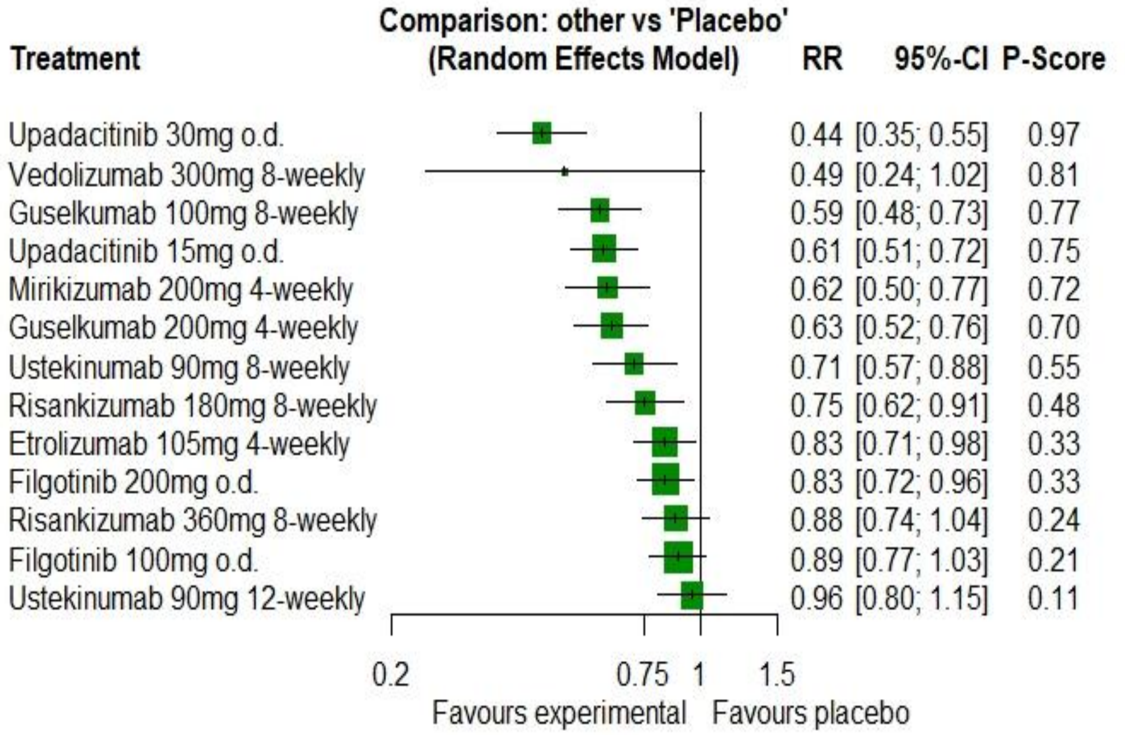

Note: The P-score is the probability of each intervention being ranked as best in the network.

Supplementary Figure 10. Network Plot for Failure to Achieve Endoscopic Remission in Trials Re-randomising Patients with UC.

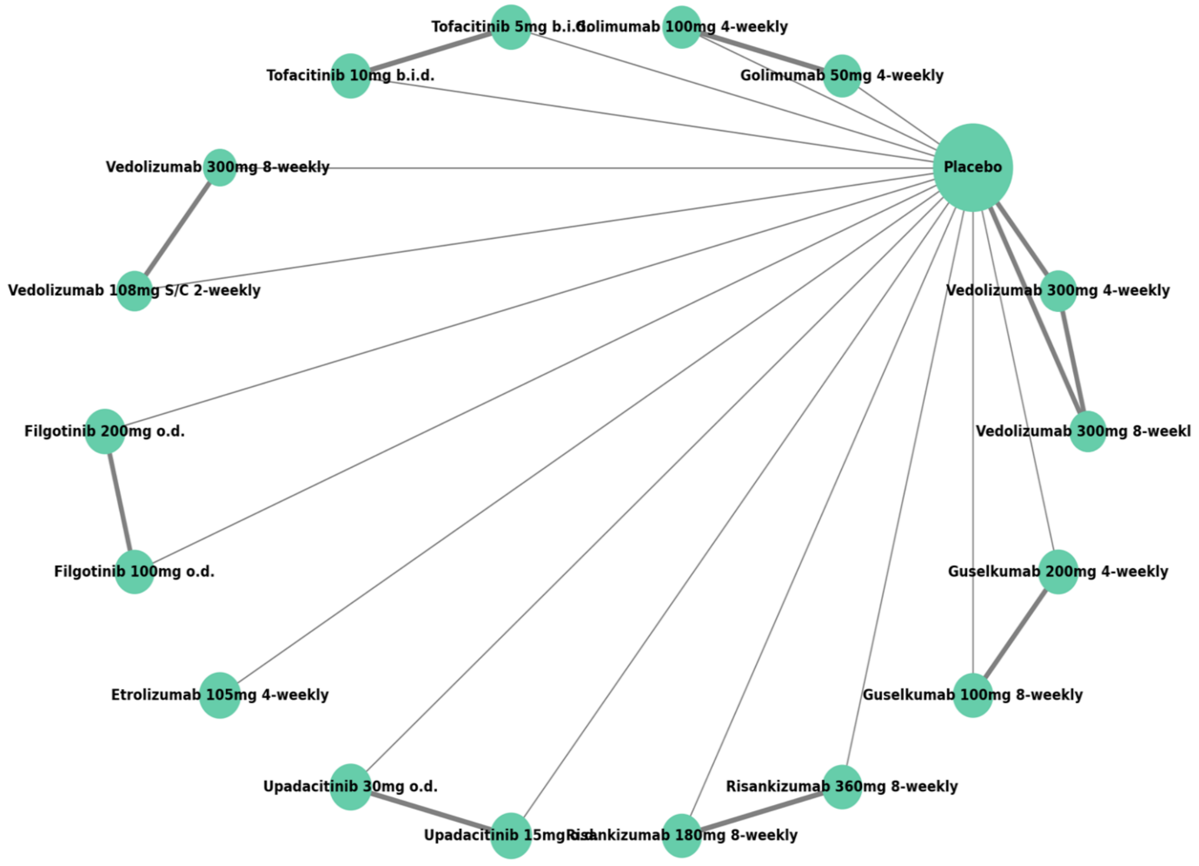

Note: Circle (node) size is proportional to the number of study participants assigned to receive each intervention. The line width (connection size) corresponds to the number of studies comparing the individual interventions.

Supplementary Figure 11. Funnel Plot for Failure to Achieve Endoscopic Remission in Trials Re-randomising Patients with UC.

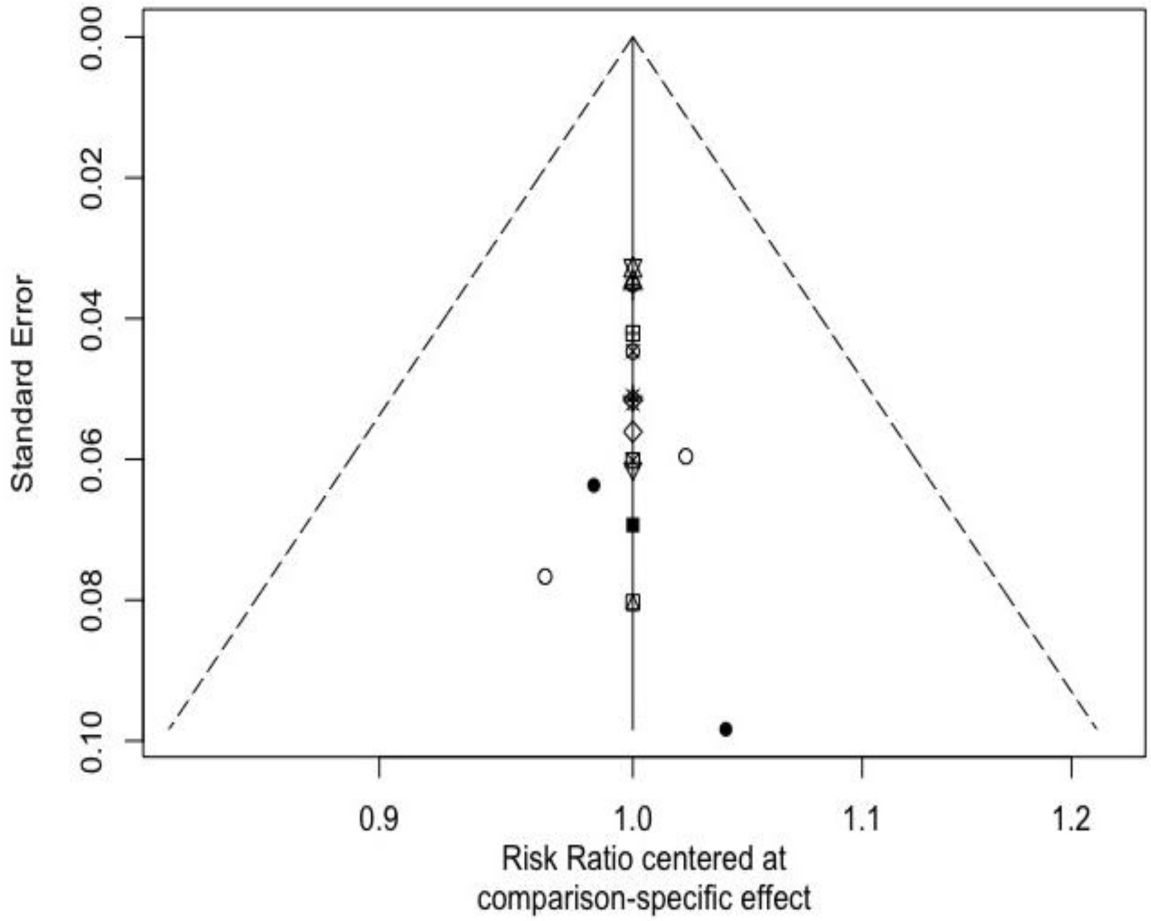

Supplementary Figure 12. Forest Plot for Failure to Achieve Endoscopic Remission in Trials Re-randomising Patients with UC Naïve to Advanced Therapies.

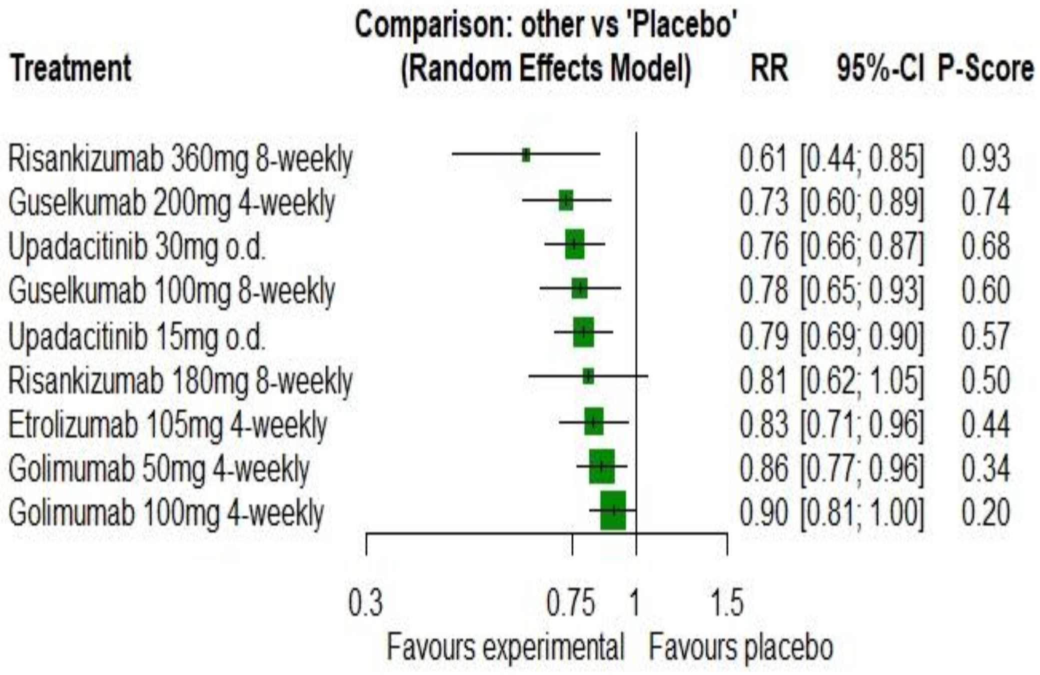

Note: The P-score is the probability of each intervention being ranked as best in the network.

Supplementary Figure 13. Forest Plot for Failure to Achieve Endoscopic Remission in Trials Re-randomising Patients with UC Exposed to Advanced Therapies.

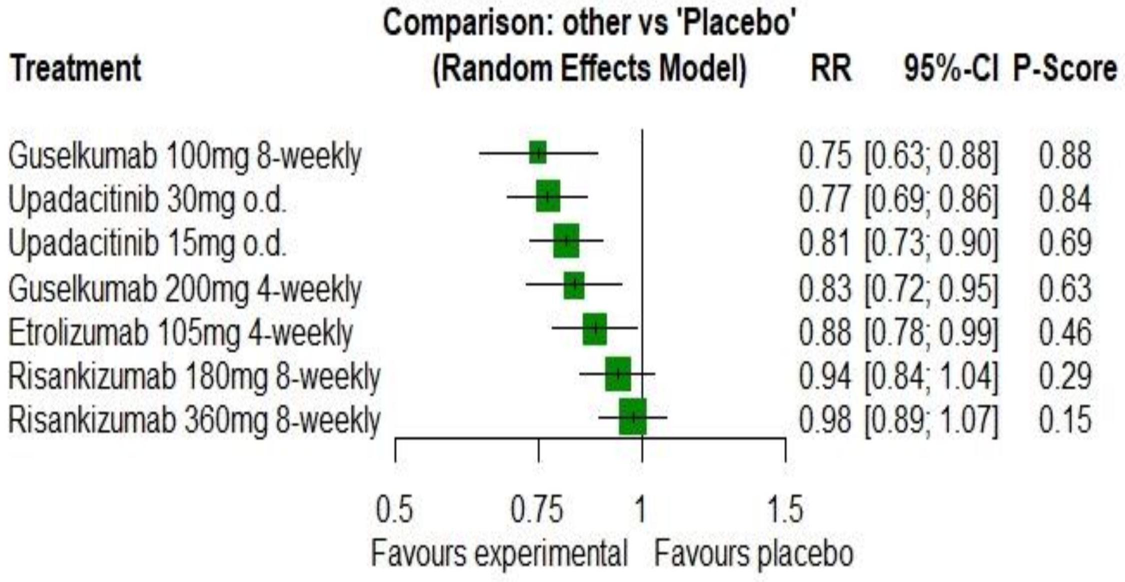

Note: The P-score is the probability of each intervention being ranked as best in the network.

Supplementary Figure 14. Network Plot for Failure to Achieve Corticosteroid-free Remission in Trials Re-randomising Patients with UC.

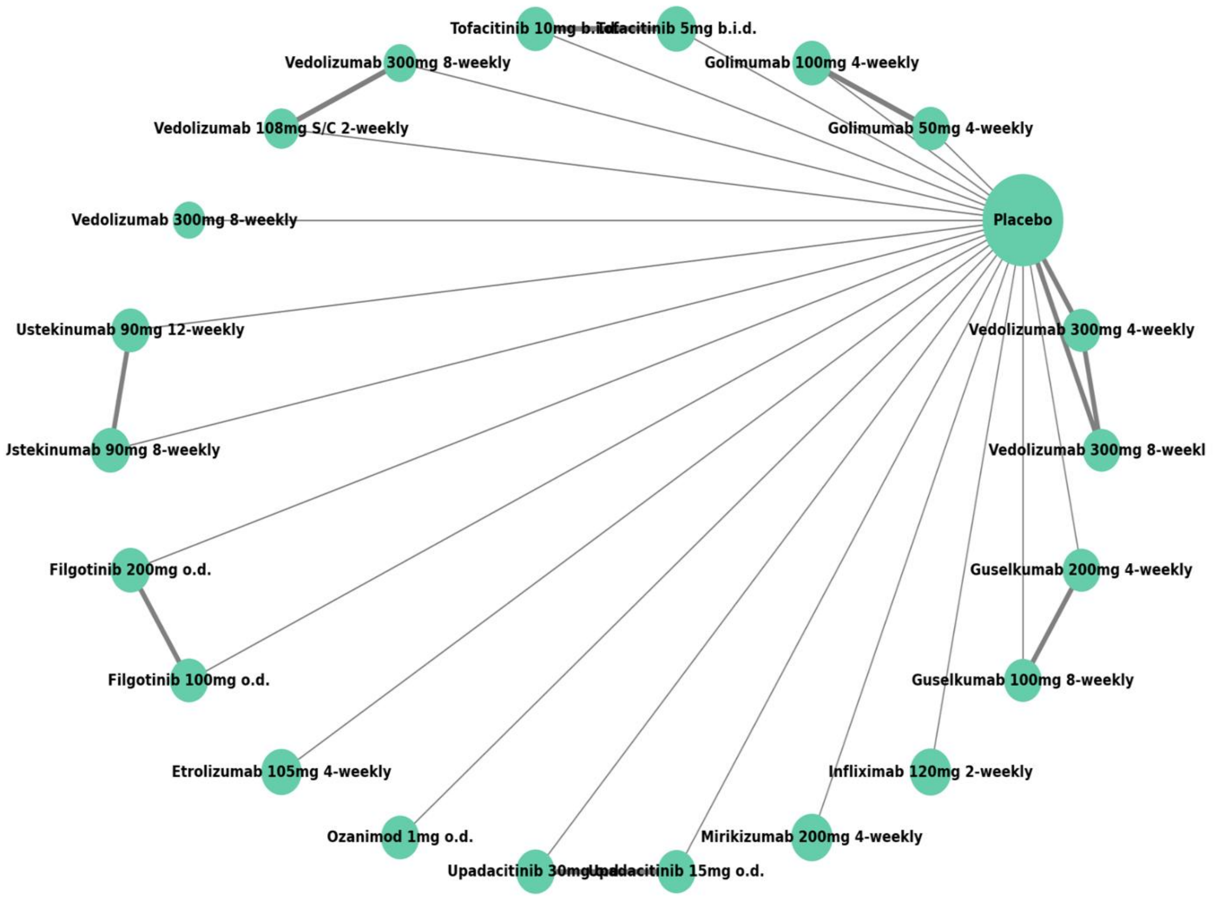

Note: Circle (node) size is proportional to the number of study participants assigned to receive each intervention. The line width (connection size) corresponds to the number of studies comparing the individual interventions.

Supplementary Figure 15. Funnel Plot for Failure to Achieve Corticosteroid-free Remission in Trials Re-randomising Patients with UC.

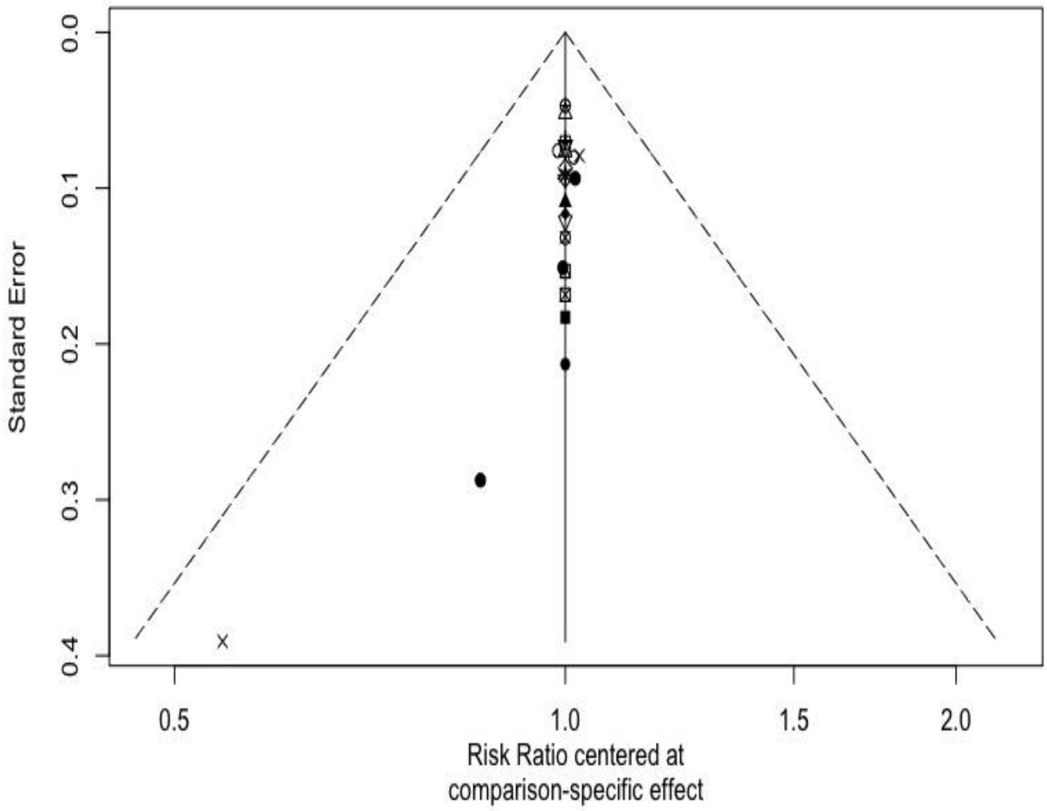

Supplementary Figure 16. Forest Plot for Failure to Achieve Corticosteroid-free Remission in Trials Re-randomising Patients with UC Naïve to Advanced Therapies.

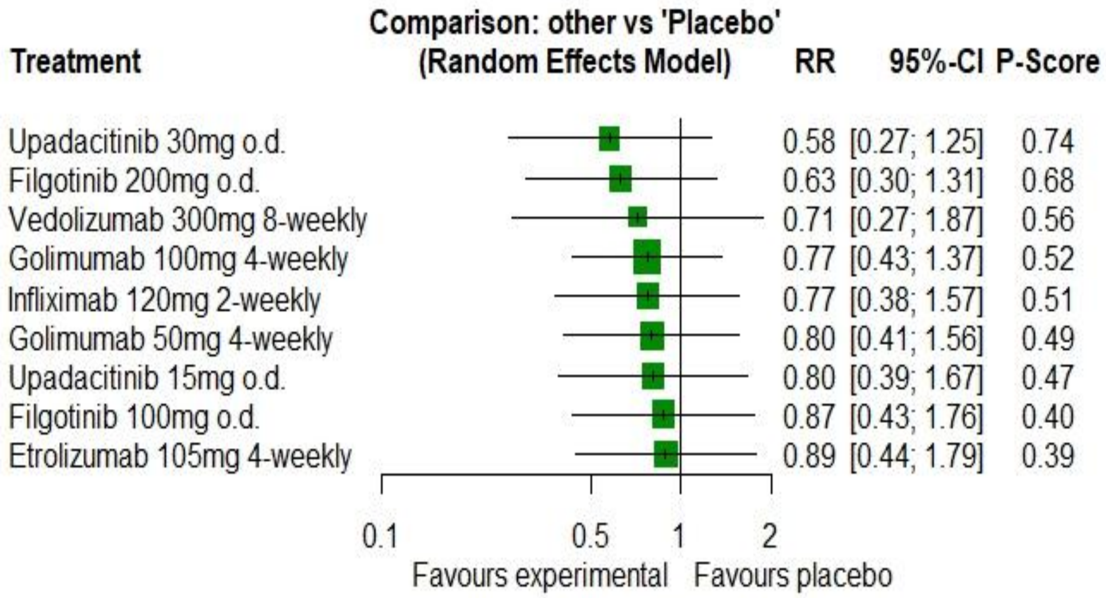

Note: The P-score is the probability of each intervention being ranked as best in the network.

Supplementary Figure 17. Forest Plot for Failure to Achieve Corticosteroid-free Remission in Trials Re-randomising Patients with UC Exposed to Advanced Therapies.

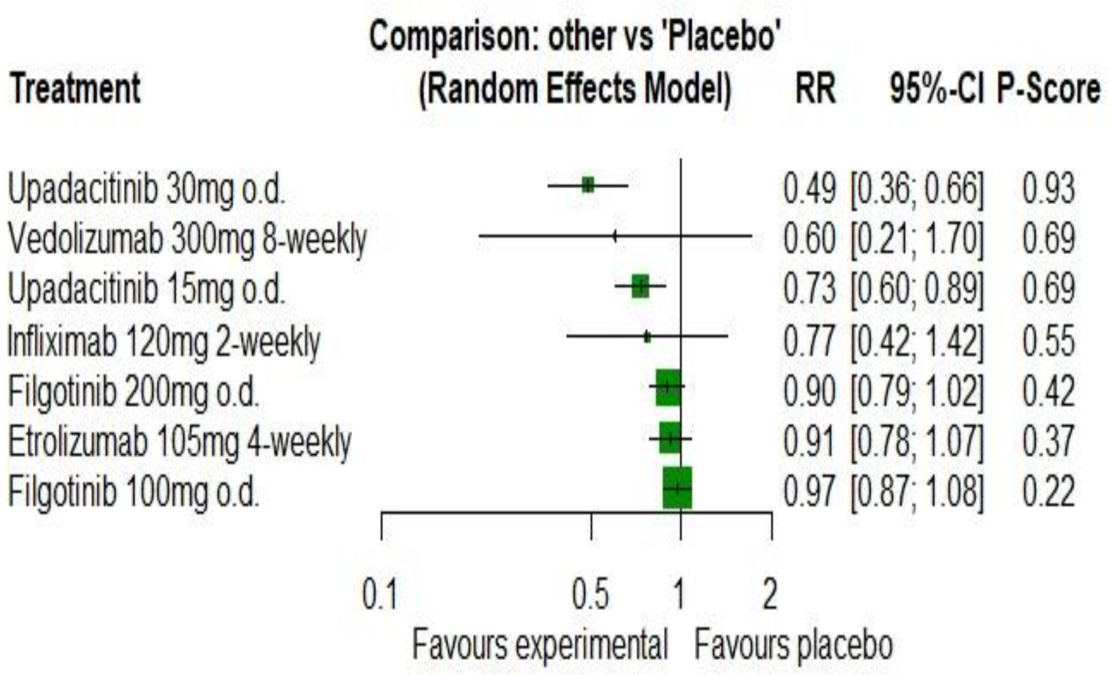

Note: The P-score is the probability of each intervention being ranked as best in the network.

Supplementary Figure 18. Forest Plot for Failure to Achieve Histological-Endoscopic Mucosal Improvement in Trials Re-randomising Patients with UC.

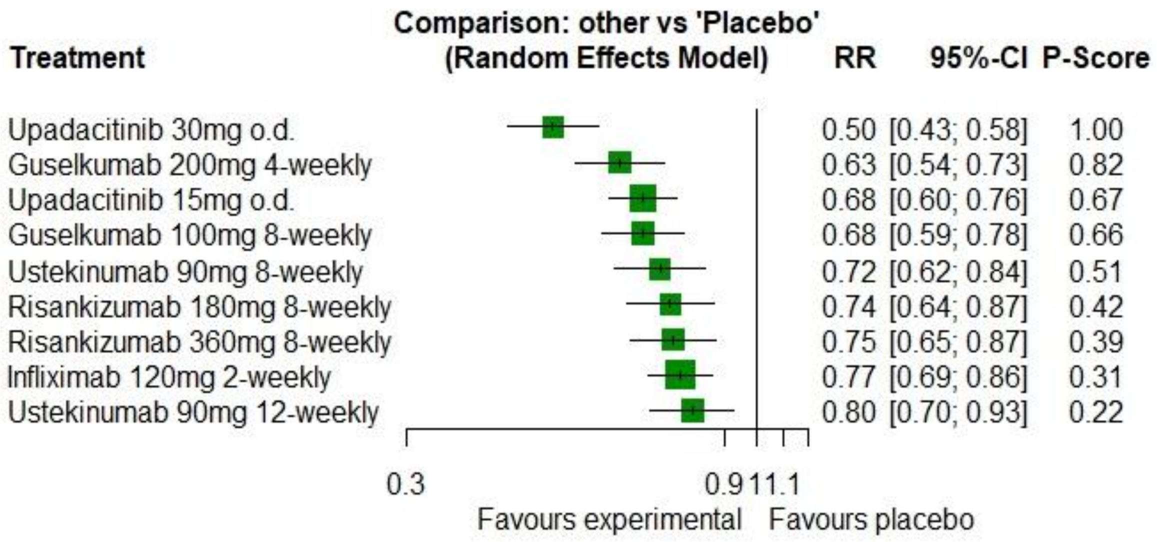

Note: The P-score is the probability of each intervention being ranked as best in the network.

Supplementary Figure 19. Forest Plot for Failure to Achieve Histological-Endoscopic Mucosal Remission in Trials Re-randomising Patients with UC.

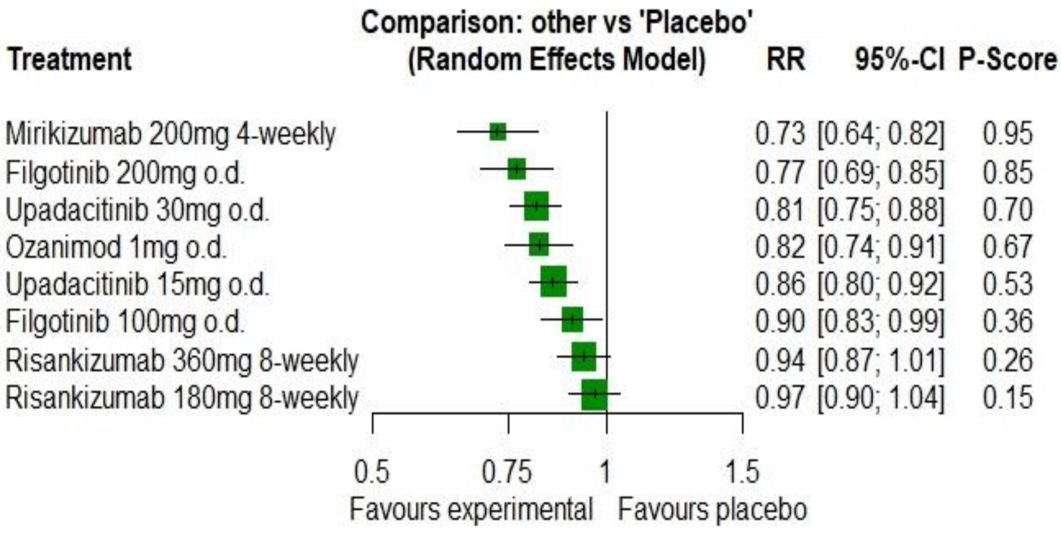

Note: The P-score is the probability of each intervention being ranked as best in the network.

Supplementary Figure 20. Forest Plot for Failure to Achieve Histological Remission in Trials Re-randomising Patients with UC.

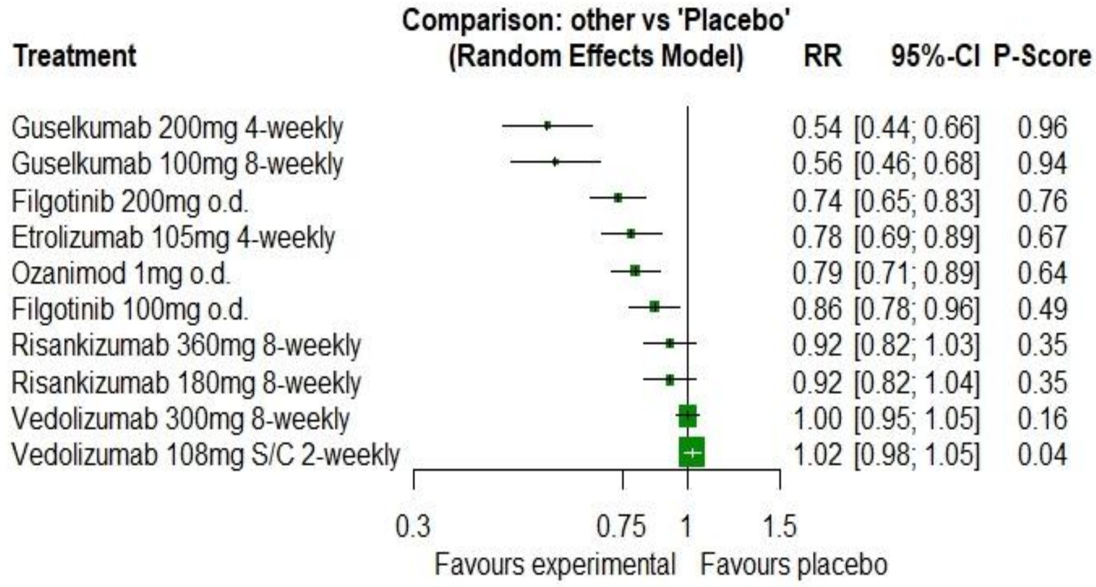

Note: The P-score is the probability of each intervention being ranked as best in the network.

Supplementary Figure 21. Network Plot for Failure to Achieve Clinical Remission in Trials Treating Patients with UC Through.

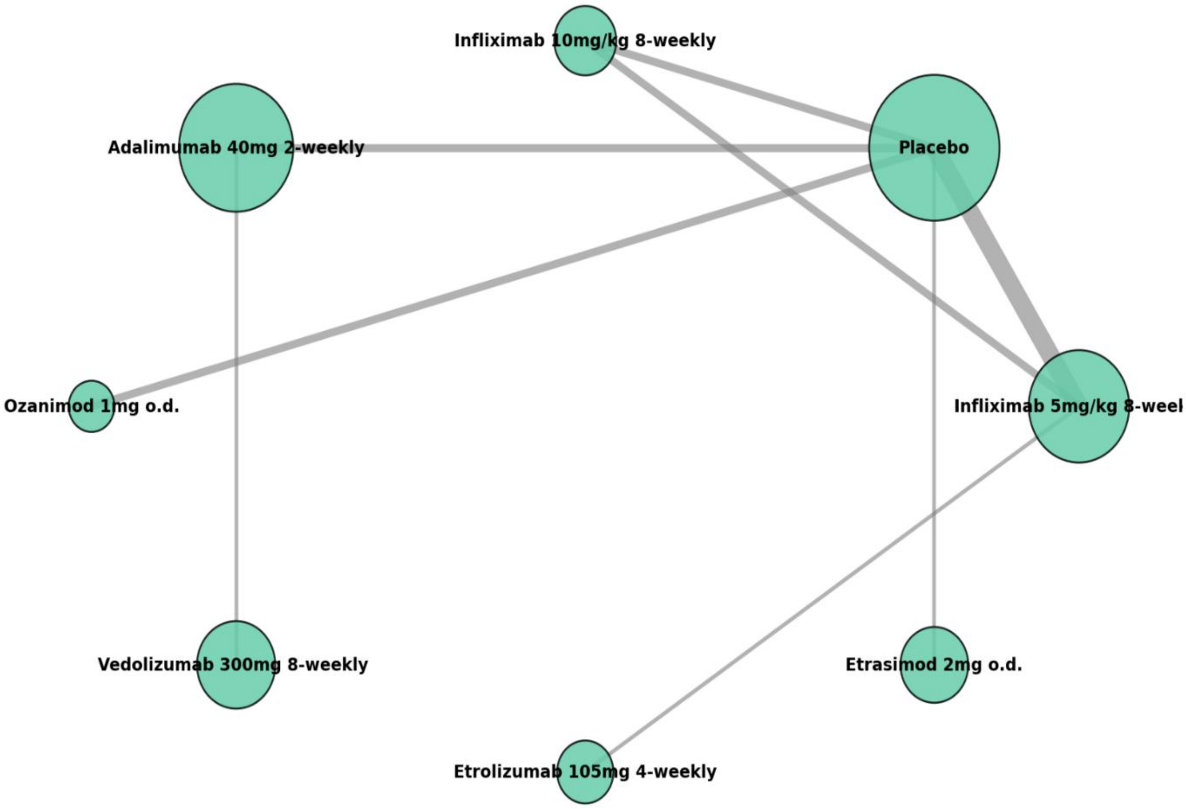

Note: Circle (node) size is proportional to the number of study participants assigned to receive each intervention. The line width (connection size) corresponds to the number of studies comparing the individual interventions.

Supplementary Figure 22. Funnel Plot for Failure to Achieve Clinical Remission in Trials Treating Patients with UC Through.

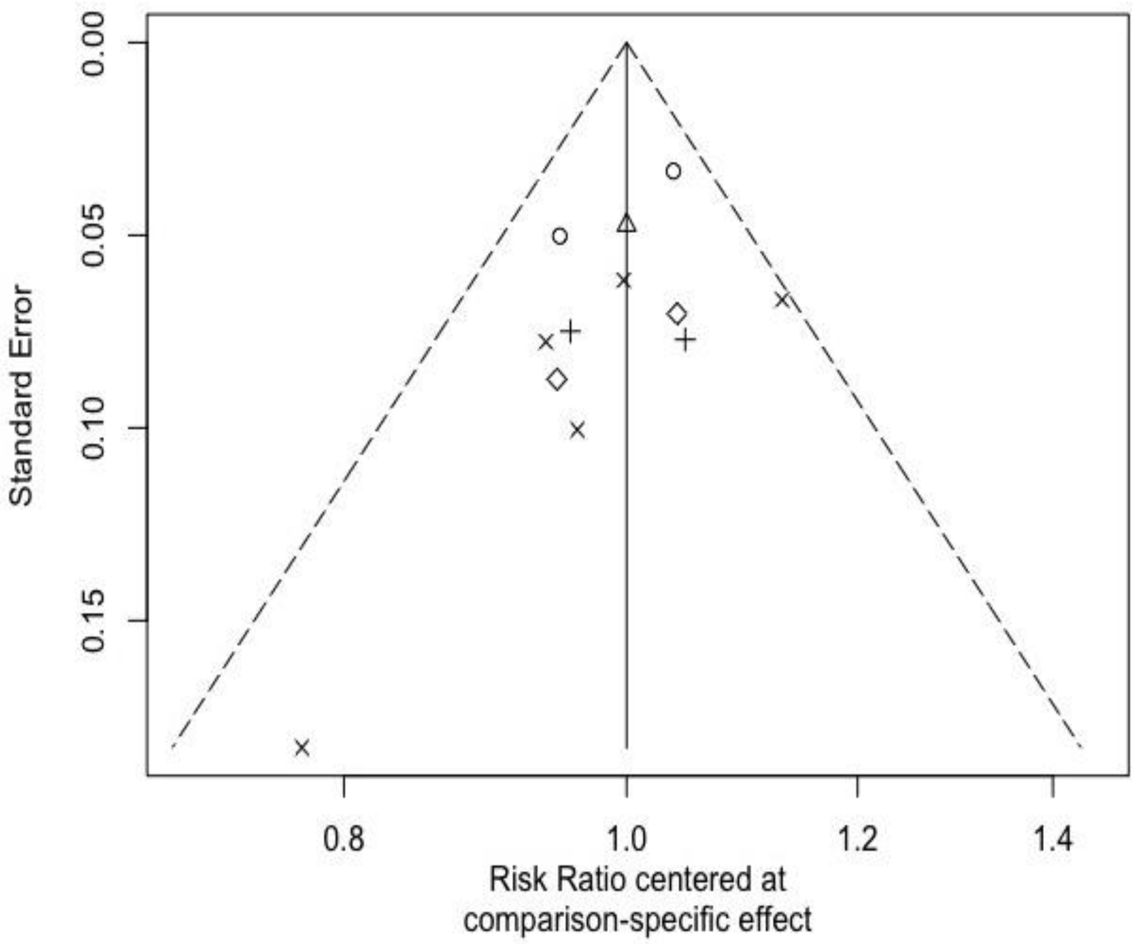

Supplementary Figure 23. Forest Plot for Failure to Achieve Clinical Remission in Trials Treating Patients with UC Through.

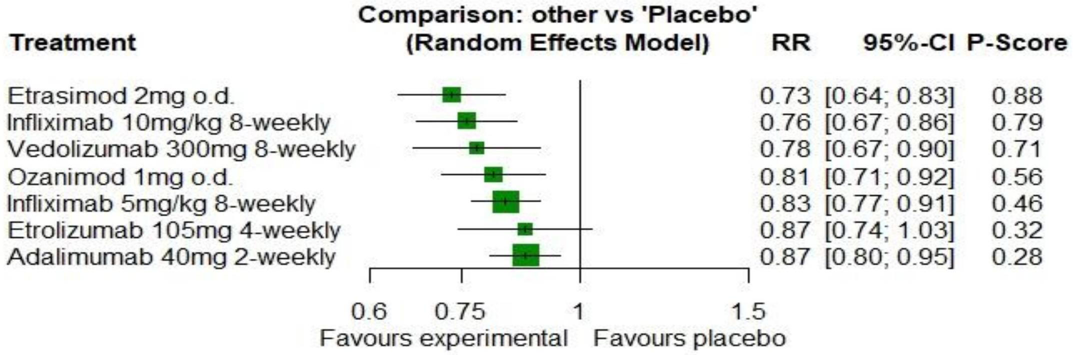

Note: The P-score is the probability of each intervention being ranked as best in the network.

Supplementary Figure 24. Forest Plot for Failure to Achieve Clinical Remission in Trials Treating Patients with UC Naïve to Advanced Therapies Through.

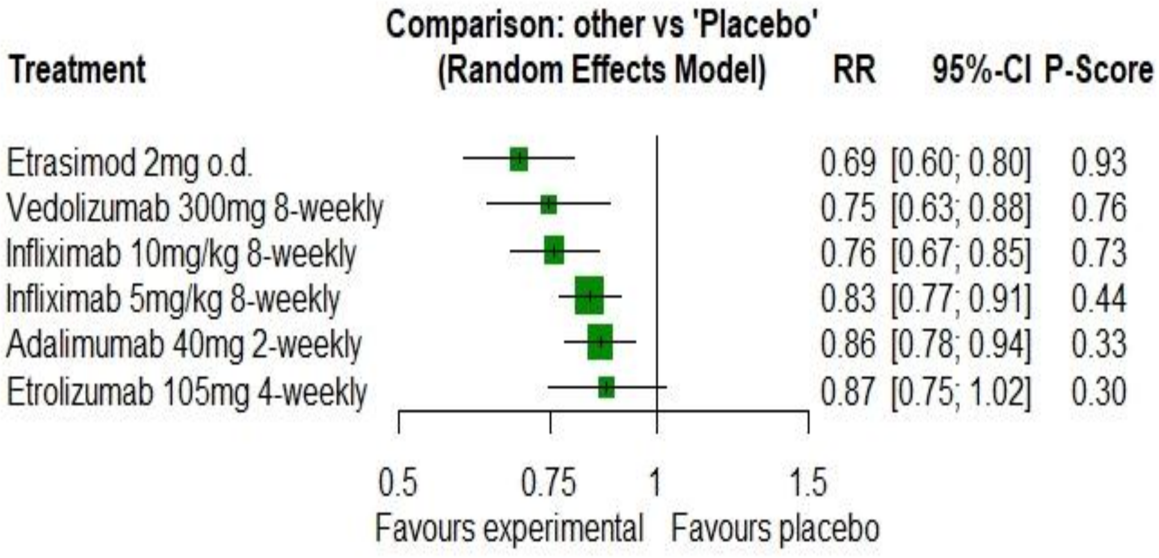

Note: The P-score is the probability of each intervention being ranked as best in the network.

Supplementary Figure 25. Forest Plot for Failure to Achieve Clinical Remission in Trials Treating Patients with UC Exposed to Advanced Therapies Through.

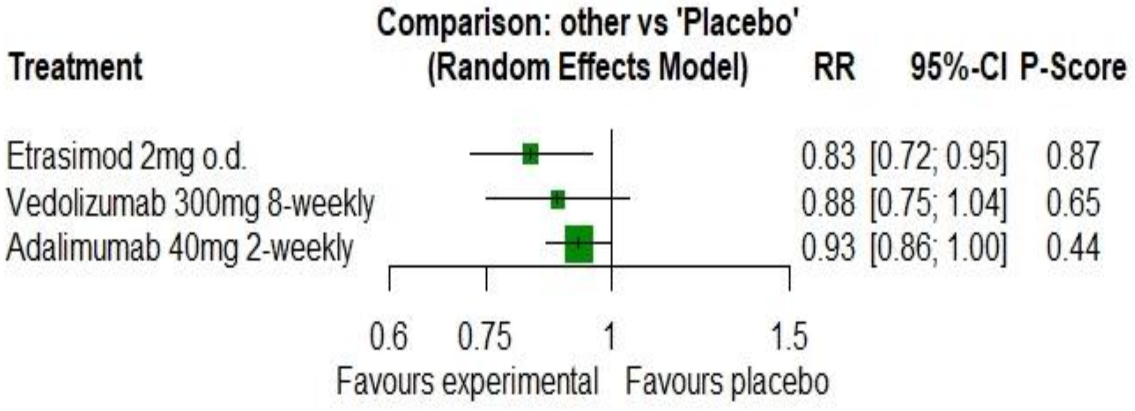

Note: The P-score is the probability of each intervention being ranked as best in the network.

Supplementary Figure 26. Network Plot for Failure to Achieve Endoscopic Improvement in Trials Treating Patients with UC Through.

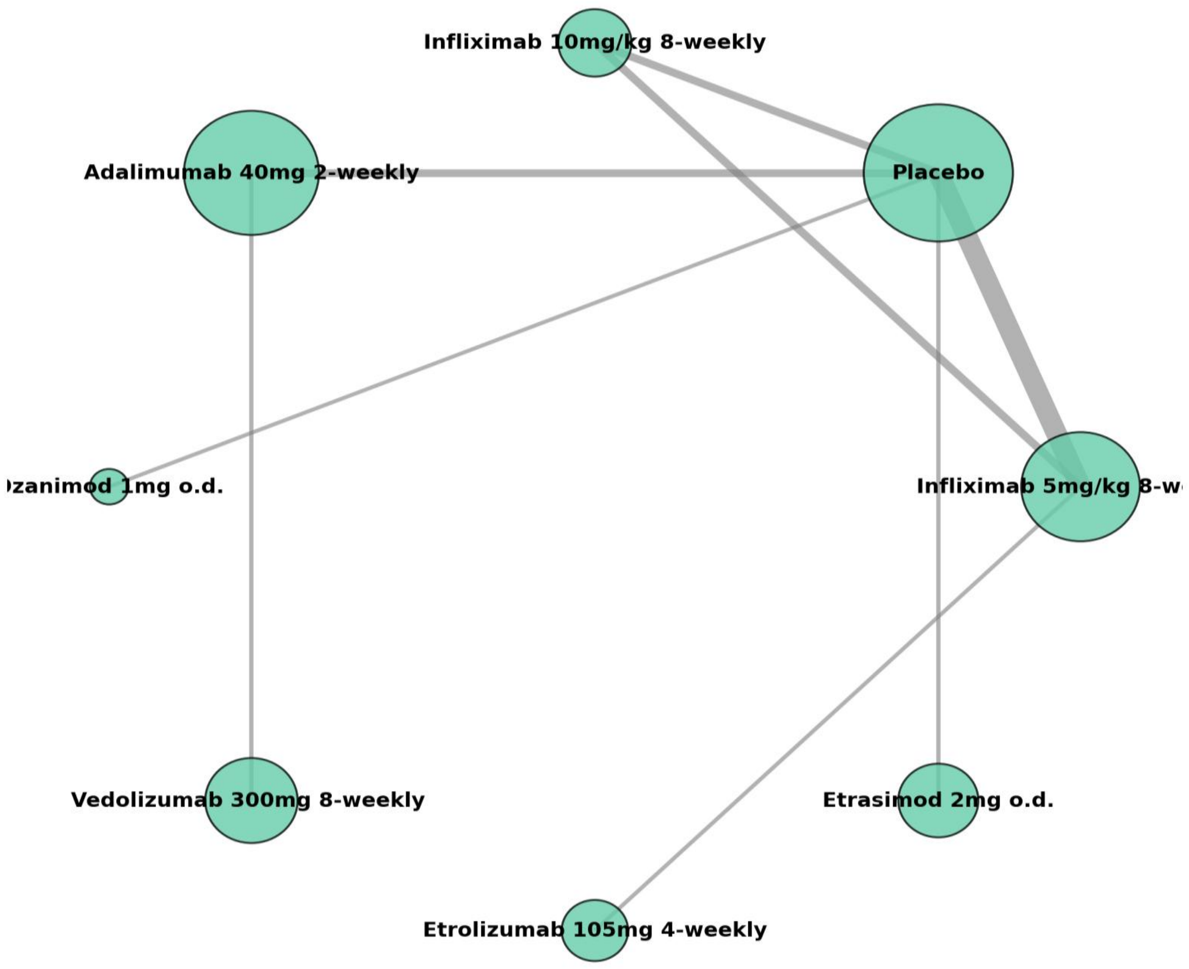

Note: Circle (node) size is proportional to the number of study participants assigned to receive each intervention. The line width (connection size) corresponds to the number of studies comparing the individual interventions.

Supplementary Figure 27. Funnel Plot for Failure to Achieve Endoscopic Improvement in Trials Treating Patients with UC Through.

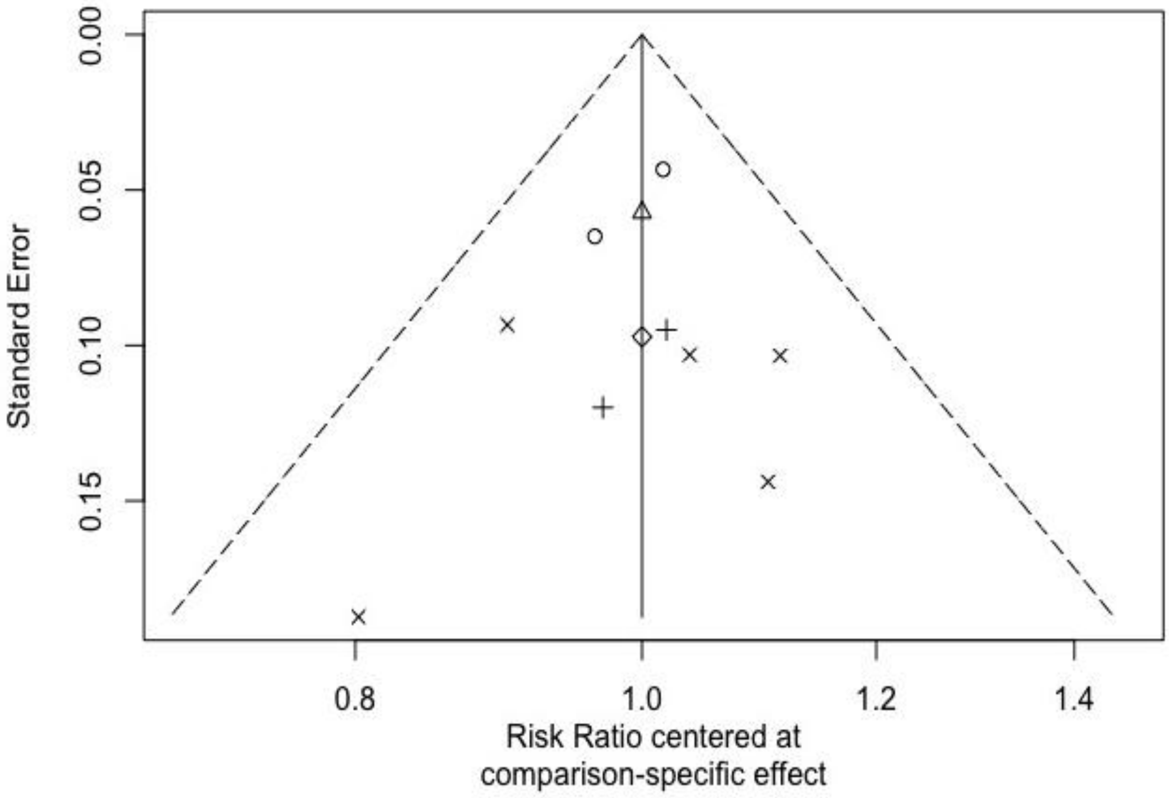

Supplementary Figure 28. Forest Plot for Failure to Achieve Endoscopic Improvement in Trials Treating Patients with UC Through.

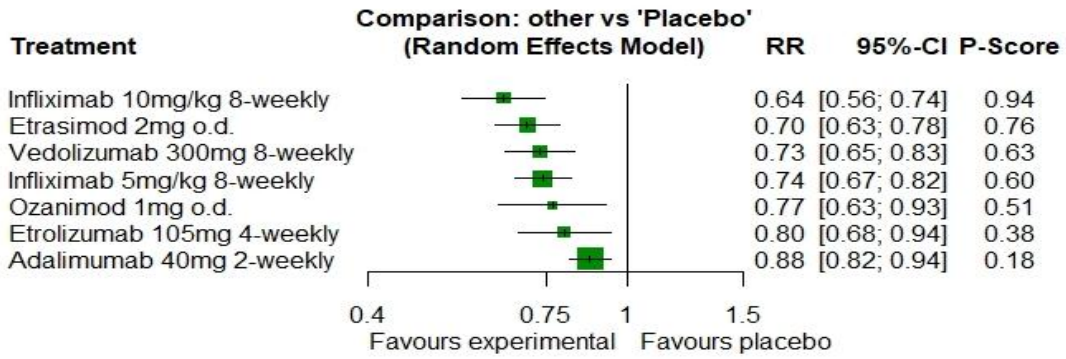

Note: The P-score is the probability of each intervention being ranked as best in the network.

Supplementary Figure 29. Forest Plot for Failure to Achieve Endoscopic Improvement in Trials Treating Patients with UC Naïve to Advanced Therapies Through.

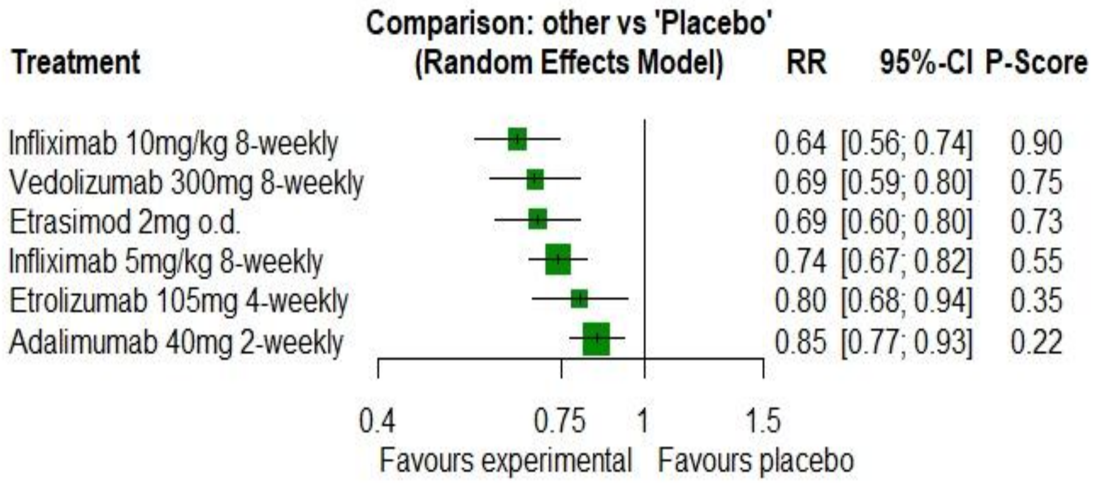

Note: The P-score is the probability of each intervention being ranked as best in the network.

Supplementary Figure 30. Forest Plot for Failure to Achieve Endoscopic Improvement in Trials Treating Patients with UC Exposed to Advanced Therapies Through.

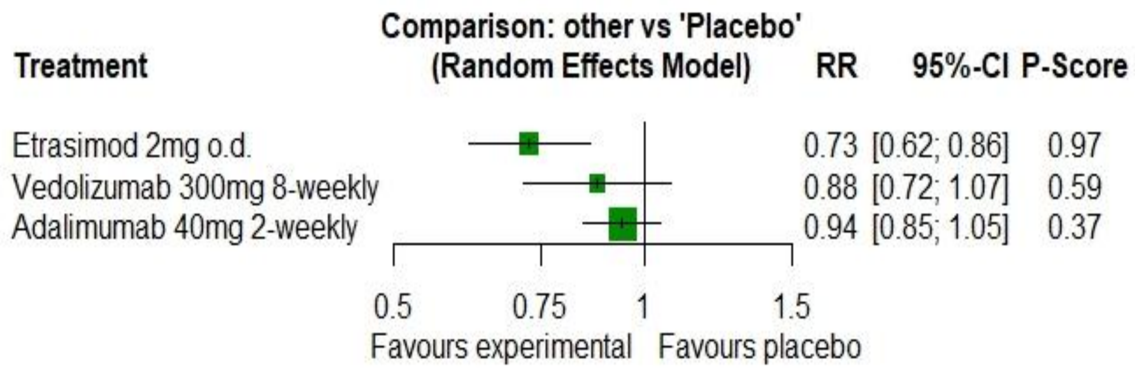

Note: The P-score is the probability of each intervention being ranked as best in the network.

Supplementary Figure 31. Network Plot for Failure to Achieve Corticosteroid-free Remission in Trials Treating Patients with UC Through.

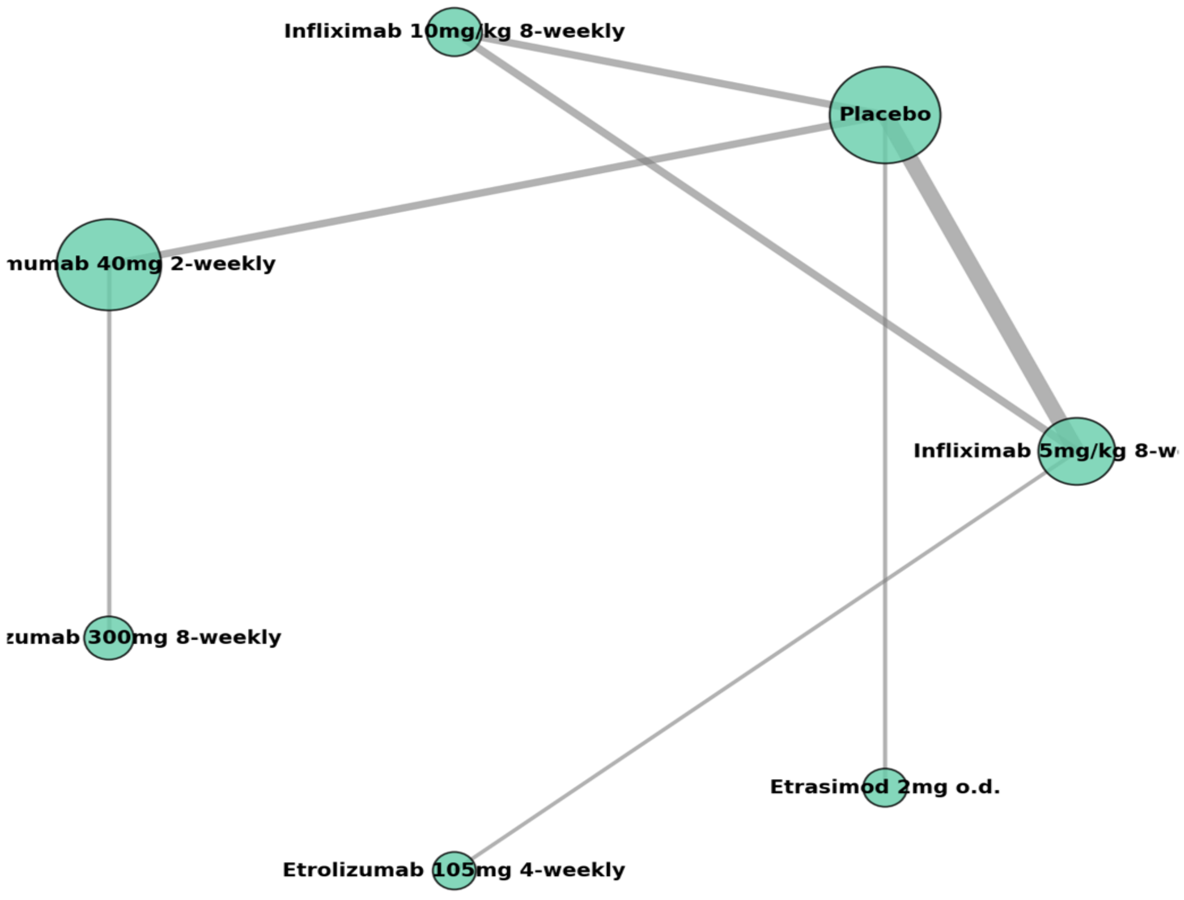

Note: Circle (node) size is proportional to the number of study participants assigned to receive each intervention. The line width (connection size) corresponds to the number of studies comparing the individual interventions.

Supplementary Figure 32. Forest Plot for Failure to Achieve Corticosteroid-free Remission in Trials Treating Patients with UC Through.

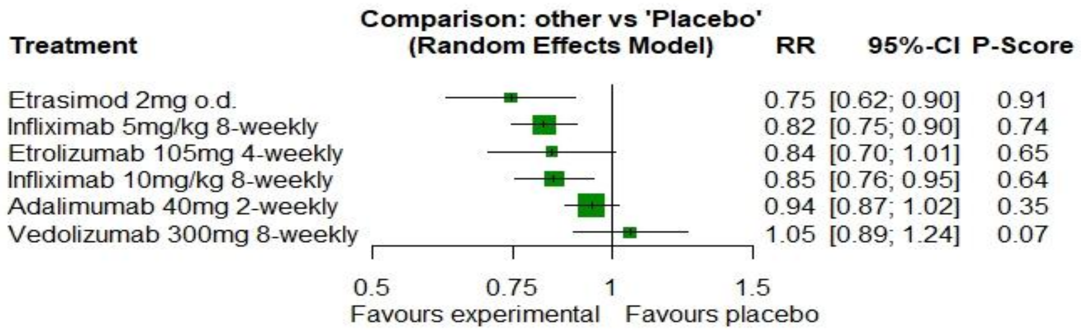

Note: The P-score is the probability of each intervention being ranked as best in the network.

Supplementary Figure 33. Forest Plot for Failure to Achieve Corticosteroid-free Remission in Trials Treating Patients with UC Naïve to Advanced Therapies Through.

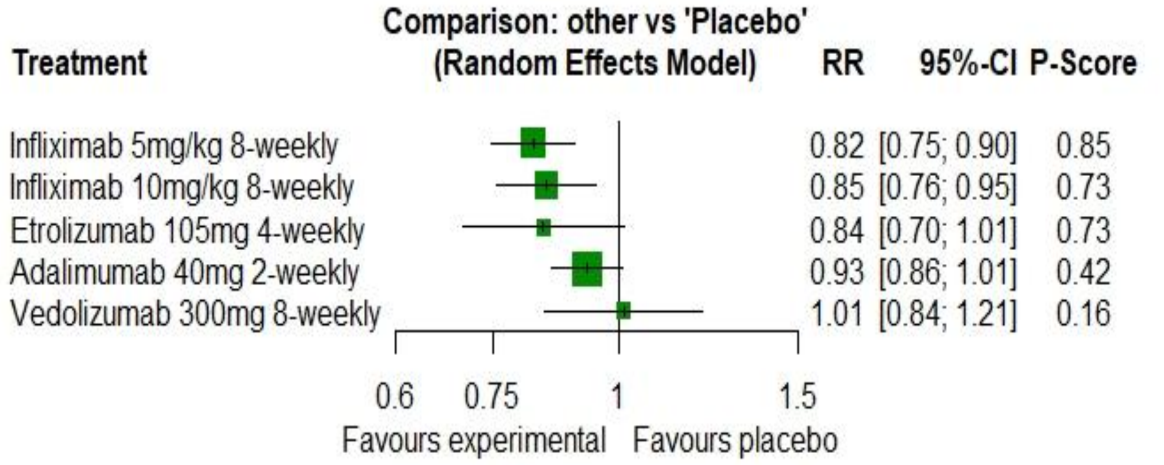

Note: The P-score is the probability of each intervention being ranked as best in the network.

Supplementary Figure 34. Forest Plot for Treatment-emergent Adverse Events in Trials Re-randomising Patients with UC.

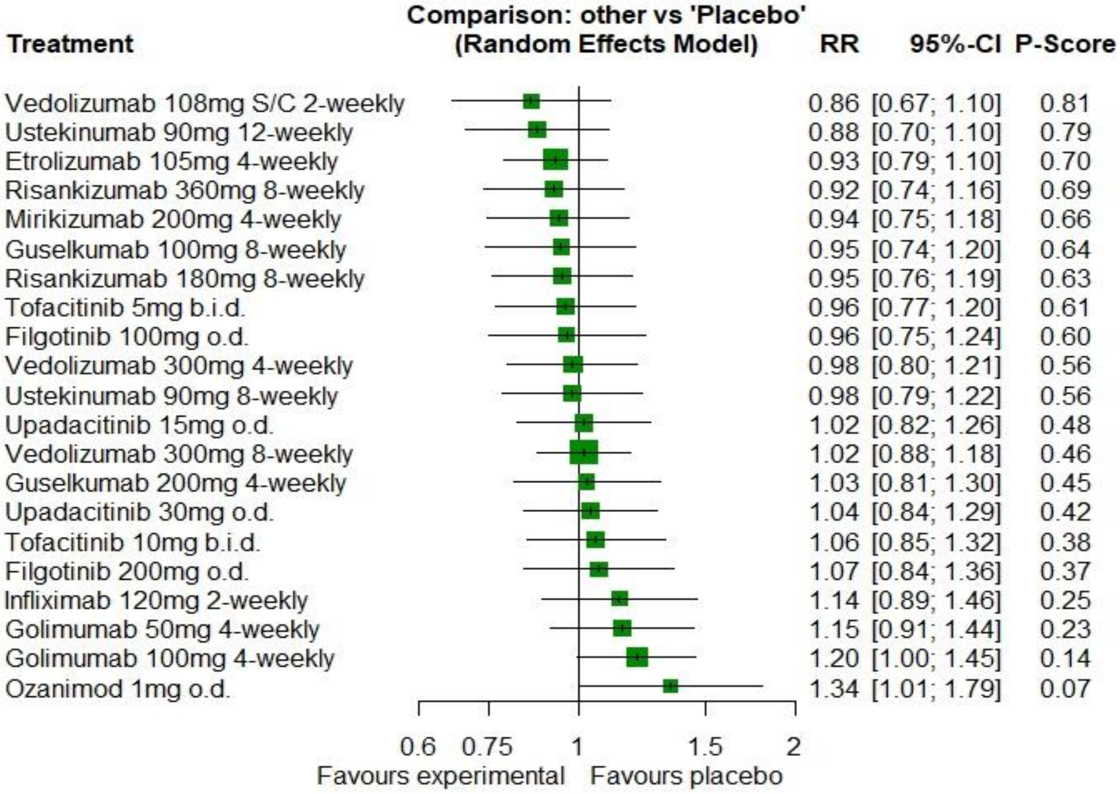

Note: The P-score is the probability of each intervention being ranked as best in the network.

Supplementary Figure 35. Forest Plot for Serious Adverse Events in Trials Re-randomising Patients with UC.

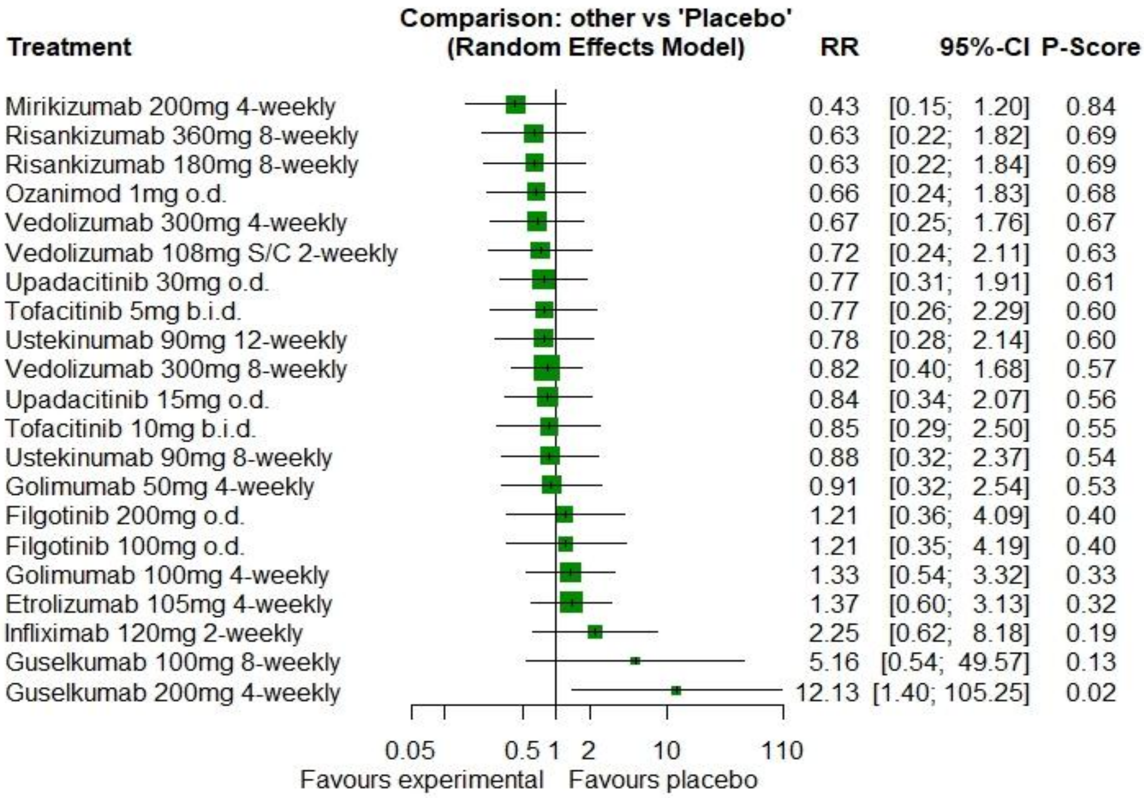

Note: The P-score is the probability of each intervention being ranked as best in the network.

Supplementary Figure 36. Forest Plot for Serious Infections in Trials Re-randomising Patients with UC.

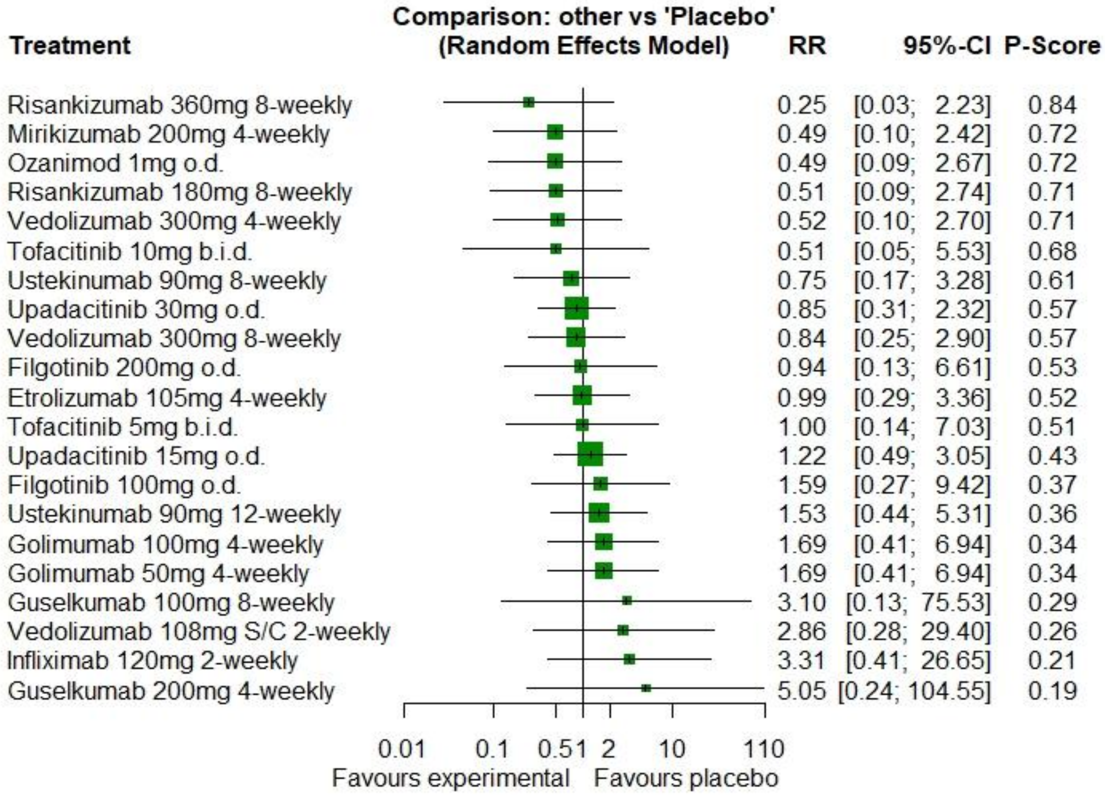

Note: The P-score is the probability of each intervention being ranked as best in the network.

Supplementary Figure 37. Forest Plot for Adverse Events Leading to Withdrawal in Trials Re-randomising Patients with UC.

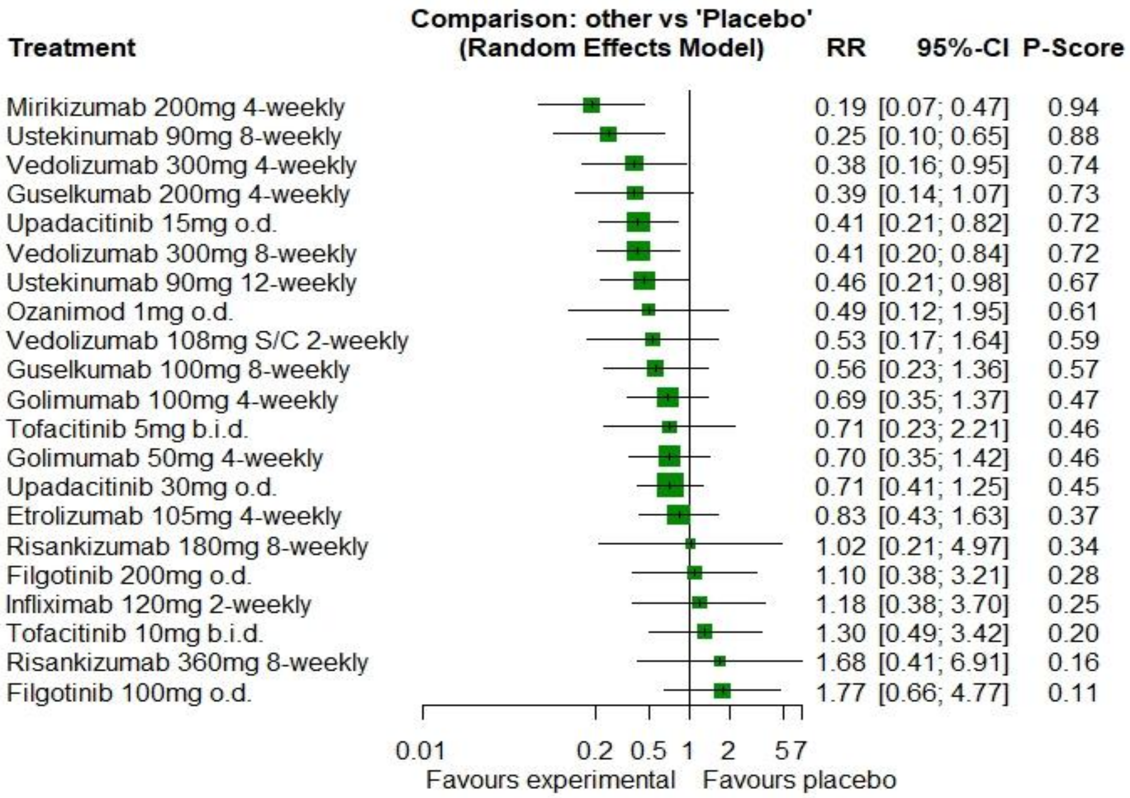

Note: The P-score is the probability of each intervention being ranked as best in the network.

Supplementary Figure 38. Forest Plot for Treatment-emergent Adverse Events in Trials Treating Patients with UC Through.

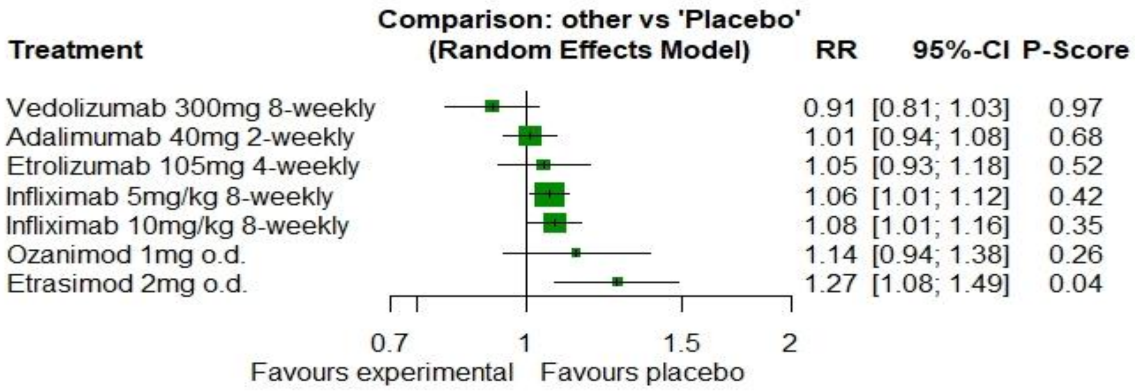

Note: The P-score is the probability of each intervention being ranked as best in the network.

Supplementary Figure 39. Forest Plot for Serious Adverse Events in Trials Treating Patients with UC Through.

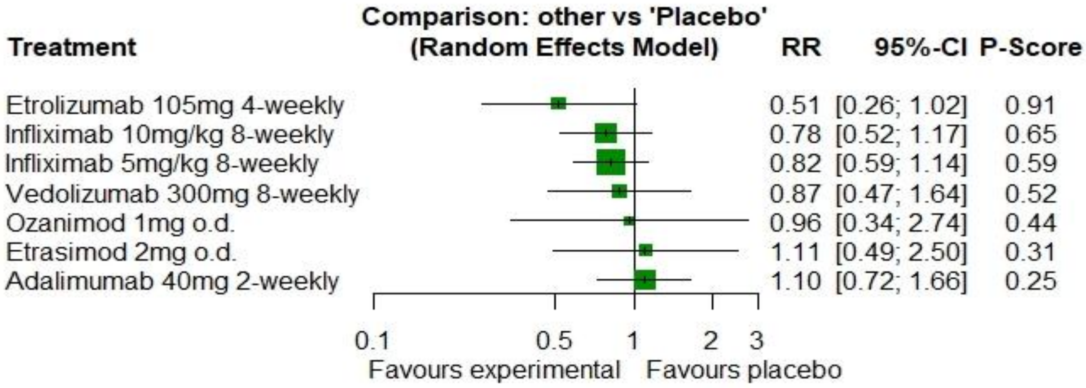

Note: The P-score is the probability of each intervention being ranked as best in the network.

Supplementary Figure 40. Forest Plot for Serious Infections in Trials Treating Patients with UC Through.

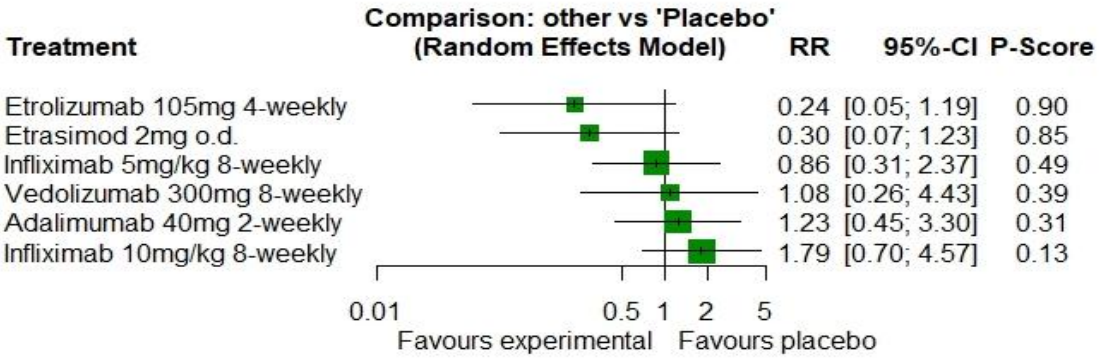

Note: The P-score is the probability of each intervention being ranked as best in the network.

Supplementary Figure 41. Forest Plot for Adverse Events Leading to Withdrawal in Trials Treating Patients with UC Through.

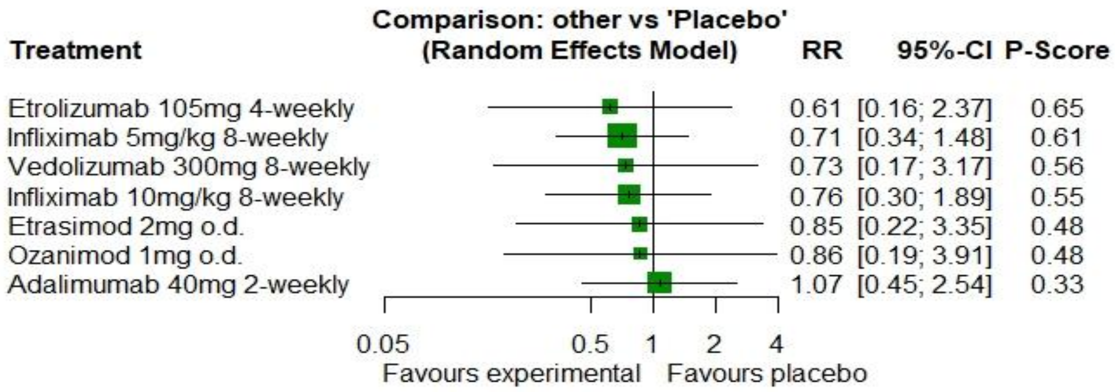

Note: The P-score is the probability of each intervention being ranked as best in the network.

REFERENCES

1. Hanauer SB, Sands BE, Schreiber S, et al. Subcutaneous infliximab (CT-P13 SC) as maintenance therapy for inflammatory bowel disease: Two randomized phase 3 trials. *Gastroenterology.* 2024;167:919-933.

2. Sandborn WJ, Feagan BG, Marano C, et al. Subcutaneous golimumab maintains clinical response in patients with moderate-to-severe ulcerative colitis. *Gastroenterology.* 2014;146:96-109.

3. Hibi T, Imai Y, Senoo A, Ohta K, Ukyo Y. Efficacy and safety of golimumab 52-week maintenance therapy in Japanese patients with moderate to severely active ulcerative colitis: A phase 3, double-blind, randomized, placebo-controlled study-(PURSUIT-J study). *J Gastroenterol.* 2017;52:1101-1111.

4. Feagan BG, Rutgeerts P, Sands BE, et al. Vedolizumab as induction and maintenance therapy for ulcerative colitis. *N Engl J Med.* 2013;369:699-710.

5. Motoya S, Watanabe K, Ogata H, et al. Vedolizumab in Japanese patients with ulcerative colitis: A Phase 3, randomized, double-blind, placebo-controlled study. *PLoS One.* 2019;14:e0212989.

6. Sandborn WJ, Baert F, Danese S, et al. Efficacy and safety of vedolizumab subcutaneous formulation in a randomized trial of patients with ulcerative colitis. *Gastroenterology.* 2020;158:562-572.e512.

7. Peyrin-Biroulet L, Hart A, Bossuyt P, et al. Etrolizumab as induction and maintenance therapy for ulcerative colitis in patients previously treated with tumour necrosis factor inhibitors (HICKORY): A phase 3, randomised, controlled trial. *The lancet Gastroenterology & hepatology*. 2022;7:128-140.

8. Vermeire S, Lakatos PL, Ritter T, et al. Etrolizumab for maintenance therapy in patients with moderately to severely active ulcerative colitis (LAUREL): A randomised, placebo-controlled, double-blind, phase 3 study. *The lancet Gastroenterology & hepatology*. 2022;7:28-37.

9. Sands BE, Sandborn WJ, Panaccione R, et al. Ustekinumab as induction and maintenance therapy for ulcerative colitis. *N Engl J Med*. 2019;381:1201-1214.

10. D'Haens G, Dubinsky M, Kobayashi T, et al. Mirikizumab as induction and maintenance therapy for ulcerative colitis. *N Engl J Med*. 2023;388:2444-2455.

11. Louis E, Schreiber S, Panaccione R, et al. Risankizumab for ulcerative colitis: Two randomized clinical trials. *JAMA*. 2024;332:881-897.

12. Rubin DT, Allegretti JR, Panés J, et al. Guselkumab in patients with moderately to severely active ulcerative colitis (QUASAR): Phase 3 double-blind, randomised, placebo-controlled induction and maintenance studies. *Lancet*. 2025;405:33-49.

13. Sandborn WJ, Su C, Sands BE, et al. Tofacitinib as induction and maintenance therapy for ulcerative colitis. *N Engl J Med*. 2017;376:1723-1736.

14. Feagan BG, Danese S, Loftus EV, Jr., et al. Filgotinib as induction and maintenance therapy for ulcerative colitis (SELECTION): A phase 2b/3 double-blind, randomised, placebo-controlled trial. *Lancet.* 2021;397:2372-2384.

15. Vermeire S, Danese S, Zhou W, et al. Efficacy and safety of upadacitinib maintenance therapy for moderately to severely active ulcerative colitis in patients responding to 8 week induction therapy (U-ACHIEVE Maintenance): Overall results from the randomised, placebo-controlled, double-blind, phase 3 maintenance study. *The lancet Gastroenterology & hepatology.* 2023;8:976-989.

16. Sandborn WJ, Feagan BG, D'Haens G, et al. Ozanimod as induction and maintenance therapy for ulcerative colitis. *N Engl J Med.* 2021;385:1280-1291.

17. Rutgeerts P, Sandborn WJ, Feagan BG, et al. Infliximab for induction and maintenance therapy for ulcerative colitis. *N Engl J Med.* 2005;353:2462-2476.

18. A study to evaluate the effectiveness and safety of infliximab in Chinese patients With active ulcerative colitis. *https://clinicaltrialsgov/study/NCT01551290*. 2014.

19. Sandborn WJ, van Assche G, Reinisch W, et al. Adalimumab induces and maintains clinical remission in patients with moderate-to-severe ulcerative colitis. *Gastroenterology.* 2012;142:257-265.

20. Suzuki Y, Motoya S, Hanai H, et al. Efficacy and safety of adalimumab in Japanese patients with moderately to severely active ulcerative colitis. *J Gastroenterol.* 2014;49:283-294.

21. Jiang XL, Cui HF, Gao J, Fan H. Low-dose infliximab for induction and maintenance treatment in Chinese patients with moderate to severe active ulcerative colitis. *J Clin Gastroenterol.* 2015;49:582-588.

22. Kobayashi T, Suzuki Y, Motoya S, et al. First trough level of infliximab at week 2 predicts future outcomes of induction therapy in ulcerative colitis-results from a multicenter prospective randomized controlled trial and its post hoc analysis. *J Gastroenterol.* 2016;51:241-251.

23. Sandborn WJ, Feagan BG, Wolf DC, et al. Ozanimod induction and maintenance treatment for ulcerative colitis. *N Engl J Med.* 2016;374:1754-1762.

24. Sands BE, Peyrin-Biroulet L, Loftus EV, Jr., et al. Vedolizumab versus adalimumab for moderate-to-severe ulcerative colitis. *N Engl J Med.* 2019;381:1215-1226.

25. Danese S, Colombel J-F, Lukas M, et al. Etrolizumab versus infliximab for the treatment of moderately to severely active ulcerative colitis (GARDENIA): A randomised, double-blind, double-dummy, phase 3 study. *The lancet Gastroenterology & hepatology.* 2022;7:118-127.

26. Sandborn WJ, Vermeire S, Peyrin-Biroulet L, et al. Etrasimod as induction and maintenance therapy for ulcerative colitis (ELEVATE): Two randomised, double-blind, placebo-controlled, phase 3 studies. *Lancet.* 2023;401:1159-1171.

27. Nakase H, Fujii T, Hisamatsu T, et al. Ozanimod as induction and maintenance therapy for ulcerative colitis: A randomized, double-blind, placebo-controlled study in Japan (J-TRUE NORTH) *Gastroenterology.* 2024;165 (Suppl 1):S817.
